# Network Pharmacology-Based Exploration on the Intervention of Qinghao Biejia Decoction on the Inflammation-Carcinoma Transformation Process of Chronic Liver Disease via MAPK and PI3k/AKT Pathway

**DOI:** 10.1155/2022/9202128

**Published:** 2022-10-14

**Authors:** Xin Cheng, Zhong-Xiao Han, Zi-Jie Su, Feng-Lin Zhang, Biao-Ping Li, Zhi-Rui Jiang, Ling Tang, Jia-Shun Yang

**Affiliations:** ^1^School of Traditional Chinese Medicine, Southern Medical University, Guangzhou 510515, China; ^2^Guangdong Provincial Key Laboratory of Chinese Medicine Pharmaceutics, Guangzhou 510515, China; ^3^Guangdong Provincial Engineering Laboratory of Chinese Medicine Preparation Technology, Guangzhou 510515, China; ^4^The Third Affiliated Hospital of Southern Medical University, Guangzhou 510630, China

## Abstract

Chronic liver disease(CLD) is a slow-developing and long-term disease that can cause serious damage to the liver. Thus far, it has been associated with viral hepatitis, non-alcoholic fatty liver disease(NAFLD), alcoholic liver disease(ALD), hepatic fibrosis(HF), liver cirrhosis (LC), and liver cancer. Qinghao Biejia Decoction (QBD) is a classic ancient Chinese herbal prescription with strong immune-enhancing, anti-inflammatory, and anti-tumor effects. In this study, we used a network pharmacology approach to investigate the molecular mechanisms of QBD in the inflammation-carcinoma transformation process of chronic liver disease. Two key drug targets, MAPK1 and PIK3CA, were screened using network pharmacology and molecular docking techniques, revealing dihydroartemisinin, artesunate, 12-O-Nicotinoylisolineolone, caffeic acid, and diincarvilone A as active ingredients involved in QBD mechanisms. The main signaling pathways involved were the PI3K-AKT signaling pathway and MAPK signaling pathway. In summary, our results indicated that QBD affects the inflammatory transformation of chronic liver disease through MAPK1 and PIK3CA and signaling pathways MAPK and PI3K/AKT. These data provide research direction for investigating the mechanisms underlying the inflammation-carcinoma transformation process in QBD for chronic liver disease.

## 1. Introduction

Chronic liver disease (CLD) is a progressive morphology-based chronic disease of the liver, with a long disease course and complex medical history that can severely harm people's health. It is a slow-developing and long-term disease that can cause serious damage to the liver. CLD has been closely associated with viral hepatitis, non-alcoholic fatty liver disease (NAFLD), alcoholic liver disease (ALD), hepatic fibrosis (HF), liver cirrhosis (LC), and liver cancer [1]. It often presents with clinical manifestations, such as hypochondriac pain, abdominal distension, weakness, nausea, loss of appetite, jaundice, and similar.

According to the cancer-related data from 2015, the number of patients with primary liver cancer significantly increased in China, ranking high in incidence and mortality rate among all malignant tumors [2]. Chronic hepatitis B virus (HBV) or hepatitis C virus (HCV), and hepatic fibrosis and liver cirrhosis induced by non-viral liver diseases, such as alcoholism, diabetes, non-alcoholic fatty liver disease, have been identified as the main causes of hepatocellular carcinoma (HCC) [3, 4]. Many clinical and epidemiological studies have indicated that the high incidence of gastrointestinal cancer in China is closely related to inflammation, such as chronic, persistent hepatitis and liver cancer, *Helicobacter pylori*-associated gastritis and stomach cancer, inflammatory bowel disease or polyp and colorectal cancer [5]. The development of hepatocellular carcinoma is often accompanied by a chronic uncontrollable inflammatory process of “hepatitis-cirrhosis-hepatocellular carcinoma”, which may even have a malignant transformation. Due to the damage caused by long-term chronic liver inflammation, over-repair of liver tissue-induced loss of hepatic lobule structure and function leads to liver fibrosis. The continuous progression of hepatic fibrosis leads to liver cirrhosis, which increases the risk of liver cancer on a yearly basis [1]. The factors such as viral infection, metabolic disorder, and toxic substances in the liver can cause damage to hepatocytes and death. Also, the damaged or dead cells can release intracellular components, thus further activating immune cells and leading to hepatitis. Protracted non-controllable inflammation can aggravate damage to hepatocytes and mediate their abnormal proliferation, leading to inflammation-carcinoma transformation. Hepatic fibrosis and liver cirrhosis have an important role as a bridge in the inflammation-carcinoma transformation [6].

In China, patients with chronic liver disease are often treated with traditional Chinese medicine(TCM). The traditional Chinese medicine compounds contain various components characterized by multi-level and multi-link comprehensive regulation and conforming to the complex pathologies of chronic liver disease to a certain extent. Although there is no such disease called chronic liver disease in traditional Chinese medicine, according to the symptom, it belongs to the categories of hypochondriac pain, accumulation, jaundice, tympanites, liver fixity, and abdominal mass [7]. In this study, Qinghao Biejia Decoction (QBD) was derived from Wen Bing Tiao Bian (written by Wu Jutong), which consists of Qinghao (Artemisiae Annuae Herba, AAH), Biejia (Trionycis Carapax, TC), Shengdihuang (Rehmanniae Radix, RR), Zhimu (Anemarrhenae Rhizome, ARR) and Mudanpi (Moutan Cortex, MC).

Qinghao is the dried above-ground part of Artemisia annua L., family Asteraceae. It has been traditionally used as a treatment of mild fever, hemostasis, and skin diseases [8]. It can regulate immune function and has antimicrobial, anti-tumor, antipyretic, and anti-inflammatory effects [9]. Some studies have shown that Qinghao extract can improve liver function in patients with liver dysfunction, prevent and restore liver damage, as well as maintain liver health [10, 11]. Biejia is derived from the dorsal nail of Trionyx sinensis Wiegmann, a member of the family Trionyxidae, and is widely used in clinical anti-liver fibrosis treatment for its softening and dispersion properties [12]. It also exerts anti-tumor and immunity regulation properties [13]. Biejia extract acts as an anti-fibrotic agent by inhibiting the activation of hepatic stellate cells and promoting their apoptosis, reducing extracellular matrix production and accelerating its degradation through anti-inflammatory and anti-peroxidative damage [14]. Biejia inhibits the proliferation of connective tissue, prolongs the anti-inflammatory efficacy of antibodies and is effective in controlling chronic liver disease to some extent [15]. Shengdihuang is the tuberous root of Rehmannia glutinosa Libosch. in the genus Ginseng family. It has anti-tumor, anti-ageing and immunomodulatory properties [16]. It improves liver function in patients with viral hepatitis and reduces levels of cytokines such as TNF-*α*, allowing valuable time for liver cell regeneration and repair [17]. Zhimu is the dried rhizome of Anemarrhena asphodeloides Bge. in the lily family, with anti-inflammatory, anti-tumor and hypoglycaemic effects [18]. Zhimu extract inhibits the growth of tumor cells by inducing apoptosis in hepatocellular carcinoma HepG2 cells [19]. The extract inhibits the activation of signaling pathways by down-regulating the expression of HSP27, thus exerting an anti-fibrotic effect on the liver [20]. Mudanpi is the dried root bark of Paeonia suffruticosa Andr. from the buttercup family, with antibacterial, anti-inflammatory and anti-tumor effects [21], which can protect the liver and activate the immune system, and is effective in combating liver damage and liver fibrosis [22]. Mudanpi extract can inhibit PI3K/AKT signaling pathway to affect the growth and migration of liver cancer cells [23]. In summary, QBD has immune-enhancing, anti-tumor and anti-inflammatory properties.

Network pharmacology analyzes the interaction between drugs, diseases, and their targets through biological information database. It can be used to preliminarily predict the pathways of drug action on diseases in an integrated and systematic pattern, based on which new methods for the disease treatment are found. The study methods include network construction, network analysis, and network verification [24–27]. Chinese medicine prescriptions have complex compositions and exert their healing properties through multiple components, targets, and approaches. The previous single-component and single-target research methods are incompatible with the characteristics of TCM. Network pharmacology considers multi-component, multi-target, and multi-channel characteristics, which is consistent with traditional Chinese medicine prescriptions, thus elaborating the theory, rationality and scientificity, clarifying the effectiveness, and providing a new scientific basis for the use of traditional Chinese medicine prescriptions. Molecular docking is a computational procedure that analyses the drug's conformation and orientation (referred together as the “pose”) into the binding site of a macromolecular target. It was first developed to investigate molecular recognition between large and small molecules [25, 28]. Today is used to assist different tasks of drug discovery programs, reveal the pharmacodynamic material basis of the complex chemical substance system, and improve the efficiency and pertinence of the TCM screening process [29].

**Figure 1:**
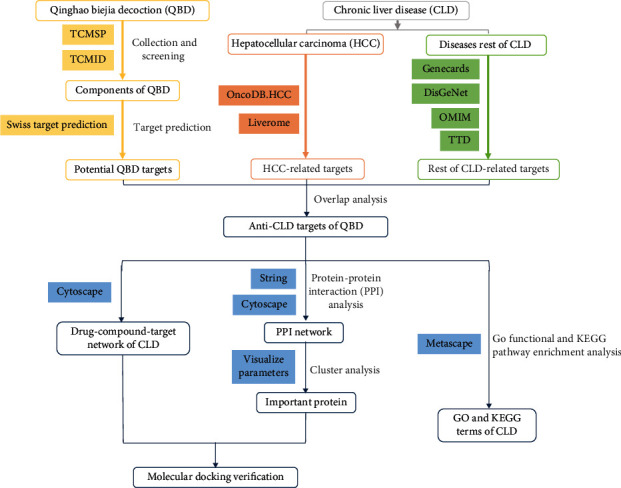
Workflow diagram of the network pharmacology study of Qinghao Biejia decoction intervention in Chronic liver disease.

Non-controllable inflammation has been identified as an early event in multiple cancers. Systematically studying the transformation process of non-controllable inflammation into cancer, exploring important nodes of inflammation-carcinoma transformation, and analyzing the key nodes of complex networks, are the critical steps for achieving early prevention, diagnosis, and treatment of cancer. In this study, we systematically studied the mechanism of Qinghao Biejia Decoction in treating inflammation-carcinoma transformation of chronic liver disease, including ALD, NAFLD, hepatitis B(HB), hepatitis C(HC), HF, LC, and HCC, using network pharmacology and molecular docking technology. The study workflow is shown in Figure 1. To the best of our knowledge, this is the first study that reported on the molecular mechanism of QBD in the inflammation-carcinoma transformation process of chronic liver disease. Our results revealed the molecular mechanism of QBD on the malignant transformation of non-controllable inflammation, thus laying a foundation for the key nodes in the transformation that could be used as cancer treatment and drug targets.

## 2. Materials and Methods

### 2.1. Active Ingredient Screening in QBD

Traditional Chinese Medicine Systems Pharmacology Database and Analysis Platform (TCMSP, http://tcmspw.com/) and comprehensive models, including oral bioavailability (OB) and drug-likeness (DL), were used to screen potential active compounds and predict ADME (absorption, distribution, metabolism, excretion) properties. According to previous research literature, most network pharmacology articles only used OB ≥30% and DL ≥0.18 as criteria for compound screening, so this method was cited in our study and the rest of ADME attributes were accepted by default. AAH, ARR, MC, RR, and TC were used as keywords for retrieval; OB ≥30% and DL ≥0.18 of components in AAH, ARR, and MC were applied for component candidate assessment. No relevant information on RR and TC was found in TCMSP, so RR and TC were used as keywords for retrieval in the Traditional Chinese Medicines Integrated Database (TCMID, http://www.tcmip.cn/). Since the results of TC in TCMID did not comply with the research range, the relevant chemical component from other literature was supplemented. The molecular structures of chemical components in RR and TC were obtained via PubChem database (https://pubchem.ncbi.nlm.nih.gov/), and the 3D structures were saved as sdf files. Next, the active compounds were screened by SwissADME database (http://www.swissadme.ch/), and more than two items from Lipinski, Ghose, Veber, Egan, and Muegge in gastrointestinal absorption were set as High, and Druglikeness was selected as YES. In the Supplement 1.

### 2.2. Target Prediction of QBD

The target protein of each active ingredient was predicted using Swiss Target Prediction (http://www.swisstargetprediction.ch/); all target proteins of compounds were downloaded, and the repeated ones were deleted. Then, assisted by the UniProt database (https://www.uniprot.org/), all the target protein names were transferred as corresponding gene names. In the supplement 1.

### 2.3. Acquisition of Therapeutic Targets for Disease

#### 2.3.1. Acquisition of Hepatocellular Carcinoma Target Genes

HCC-related human genes were collected from two databases, OncoDB.HCC database (http://oncodb.hcc.ibms.sinica.edu.tw/index.htm) and Liverome database (http://liverome.kobic.re.kr/index.php). A gene was selected if it satisfied at least one of the following criteria: (i) experimentally validated in OncoDB.HCC; (ii) occurrence frequency>7 among the various collected gene signatures in Liverome. Duplicate genes were removed from both databases. The genes were normalized using the UniProt database. A total of 566 genes were obtained. In the supplement 2.

#### 2.3.2. Acquisition of Target of Chronic Liver Diseases

The remaining six chronic liver diseases were ALD, NAFLD, HB, HC, HF, and LC. The target genes of each disease were obtained by searching the Therapeutic Target database (http://db.idrblab.net/ttd/), GeneCards database (https://www.genecards.org/), DisGeNet database (https://www.disgenet.org/), and OMIM database (https://omim.org/). The target genes obtained from the GeneCards database were screened using a median Relevance Score to obtain the final target genes. The target genes obtained from each gene database were searched one by one through the UniProtKB search function target in the UniProt database, and the species were defined as “human” and “Reviewed” in the retrieval results. After summarizing target genes obtained from each database, duplicate values were deleted, and the total number of target genes was recorded. In the supplement 3–8.

### 2.4. Network Construction

#### 2.4.1. Construction of Drug-Disease Intersection Target and Venn Diagram

The relevant targets of active components in QBD and the target of each chronic liver disease were introduced into Venny2.1.0 (http://bioinfogp.cnb.csic.es/tools/venny/index.html) to obtain drug-disease intersection targets and Venn diagrams.

#### 2.4.2. Construction of a Compound-Therapeutic Target Network

Taking the compound corresponding to a single traditional Chinese medicine as a set, there were 5 plants in the QBD in 5 sets. The treatment targets of different diseases are different sets. Cytoscape 3.7.2 was used to draw the drug-compound-therapeutic target network diagram.

#### 2.4.3. Protein-Protein Interaction Network (PPI)

Common targets of each disease were imported into the STRING database (https://string-db.org/), respectively. They were then searched with Multiple Proteins; the protein type was set as “Homo sapiens” at confidence ≥0.9, network display mode was: interactive svg, and free nodes were hidden. The tsv data files were downloaded and imported into Cytoscape 3.7.2 software for visualization analysis, and the degree centrality (DC) was calculated. Using the Visualize Parameters of Cytoscape to visualize the important nodes of the PPI network, the top 10 targets of chronic liver diseases were screened out to construct the sub-network.

#### 2.4.4. GO and KEGG Pathway Enrichment Analysis

The Gene Ontology (GO) biological function and Kyoto Encyclopedia of Genes and Genomes (KEGG) signal pathway enrichment of drug-disease intersection targets were analyzed using the Metascape database (http://metascape.org/gp/index.html#/main/step1), with the *P* value < 0.01. Combined with relevant literature, the important enrichment results were visually analyzed using Bioinformatics (http://www.bioinformatics.com.cn/) and ImageGP (http://www.ehbio.com/ImageGP/index.php/Home/Index/index.html) online mapping websites.

#### 2.4.5. Molecular Docking Verification

Molecular docking can predict the conformation of small-molecule ligand in appropriate binding sites within a relatively wide accurate range. The molecular docking algorithm was used to quantitatively predict the binding energy, and the total score of the docked compounds was ranked according to the ligand-compound binding capability. The molecular docking of compounds in QBD, important disease targets in the inflammation-carcinoma transformation process of chronic liver disease, and their interaction activity, were verified using Sybyl-X 2.0.0 software.

*(1) Small Molecule Processing*. The key active components involved in therapeutic targets of seven chronic liver diseases were selected according to the construction result of the drug-compound-therapeutic target network and PPI network. The molecular structure diagram was obtained through PubChem, and its 3D structure was saved in sdf format. Next, the file was imported into Chem3D at a minimum Root-mean-squared gradient: 0.0100 for molecular energy minimization. The small molecule compound file was exported in mol2 format.

*(2) Preparation and Treatment of Protein*. According to the construction and analysis of the PPI sub-network, the same targets from the top 10 targets of seven chronic liver diseases were screened and researched in the UniProt database to download key target proteins. The species in Popular organisms was Human, and the protein structure with Reviewed (Swiss-Prot) was selected. In the Structure, the appropriate protein structure was selected. After entering the Protein Data Bank database (PDB), the PDB file was downloaded by selecting the secondary protein structure with high structural similarity and high resolution between the original protein-ligand and the docking chemical composition. The file was imported to Sybyl-X 2.0.0, after which irrelevant original ligands and water molecules were removed from the protein through the Surflex-Dock(SFXC). The selected structure was analyzed, and the result was hydrogenated to calculate and distribute charges. The Automatic protomol docking method was selected to generate the molecular docking file in SFXC format.

*(3) Molecular Docking*. SFXC in the Sybyl-X 2.0.0 was used. Standard and fully automatic SURFLEX-dock Geom software, with an original threshold of 0.50, and the original expansion of 0, the small molecule was docked with the therapeutic target. SFXC calculated the result to determine the affinity between the small molecular compound and the therapeutic target.

## 3. Results

### 3.1. Common Target Screening of QBD for Chronic Liver Diseases

Among 177 chemical components that were obtained in QBD, 76 were AAH, 32 were ARR, 27 were MC, 20 were RR, and 22 were TC. There were 881 target genes in QBD. The search terms for chronic liver disease and the number of target genes in each database are shown in Table 1.

**Figure 2:**
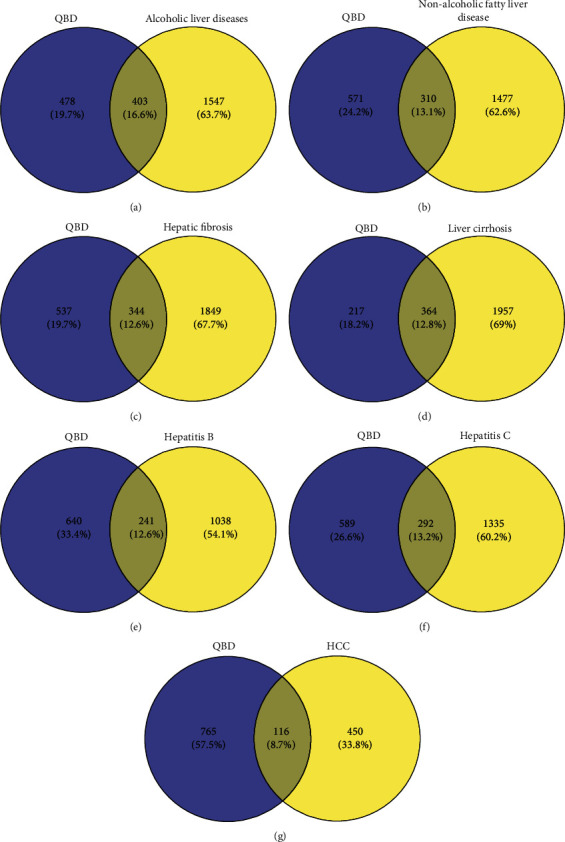
Venn diagram of QBD intervention in CLD. (a) ALD with QBD, (b) NAFLD with QBD, (c)HF with QBD, (d)LC with QBD, (e)HB with QBD, (f) HC with QBD, and (g) HCC with QBD. Yellow for disease and purple for Qinghao Biejia decoction. The overlap is the intersection target of the QBD intervention CLD.

The intersection of disease targets of seven chronic liver diseases and QBD were 310, 403, 344, 364, 241, 292, and 116, respectively, as shown in Figure 2.

### 3.2. Construction of Component-Target Network

**Figure 3:**
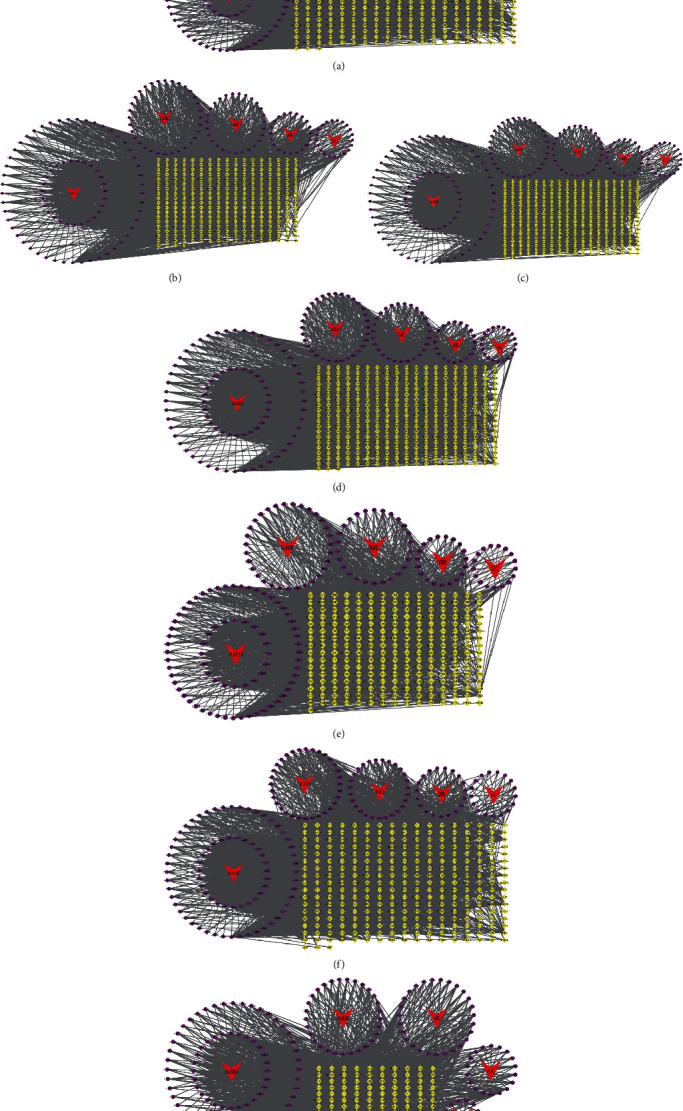
Drug-compound-target network for QBD intervention in CLD. (a) QBD intervention ALD (b) QBD intervention NAFLD (c) QBD intervention HB (d) QBD intervention HC (e) QBD intervention HF (f) QBD intervention LC, and (g) QBD intervention HCC. The red arrow is the drug in QBD, the purple circle is the active component, and the yellow diamond is the intersection target.

The therapeutic targets of seven chronic liver diseases and the drugs and active ingredients of QBD were imported into Cytoscape 3.7.2 software to construct a drug-compound-therapeutic target network of QBD. The network was analyzed and visually processed. As shown in Figure 3, the active drug components are arranged according to the size of DC.

### 3.3. Network Analysis of Disease Common Target Proteins

**Figure 4:**
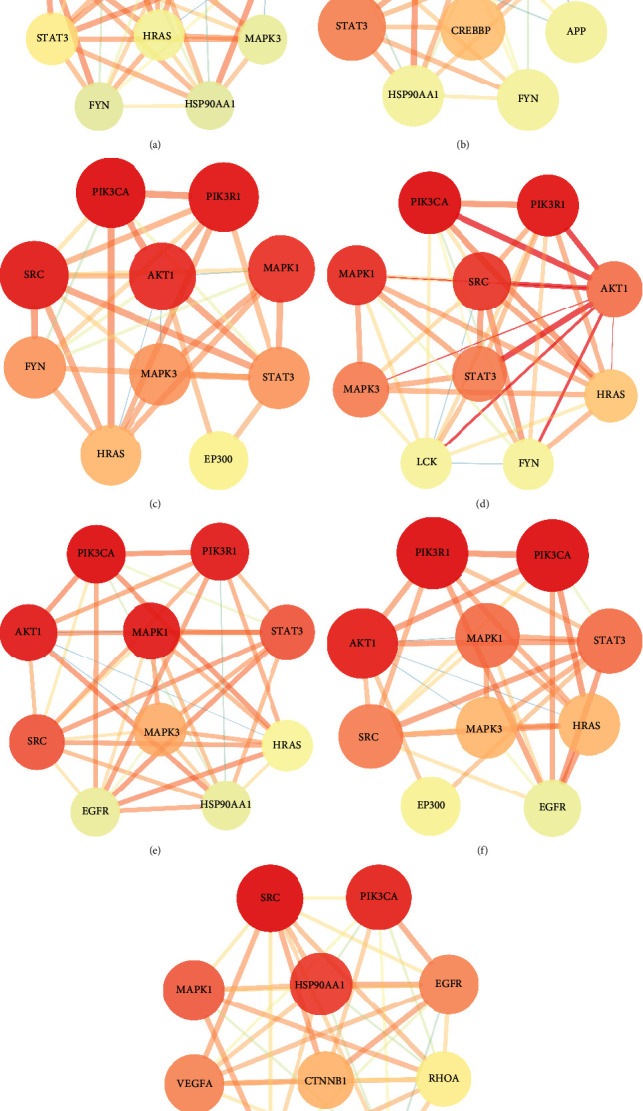
Key targets for QBD intervention in CLD. (a) QBD intervention ALD (b) QBD intervention NAFLD (c) QBD intervention HB (d) QBD intervention HC (e) QBD intervention HF (f) QBD intervention LC, and (g) QBD intervention HCC. The color changed from red to yellow, representing the DC from large to small. This means that the redder the color, the larger the DC value, the more important the target is in the network.

Target of each disease were imported into the STRING database, respectively, and the obtained PPI network was imported into Cytoscape 3.7.2 software. The top 10 key target proteins of DC were screened using the software plug-in. The highest node of DC was the central node in the pathway, in an important position for the targets' interaction. The obtained key target proteins were used to construct the sub-network, as shown in Figure 4. Among them, the key targets of seven chronic liver diseases included PIK3CA and MAPK1, and the key targets of six diseases included PIK3R1, SRC, AKT1, STAT3, and MAPK3, indicating that these seven targets had an essential role in the inflammation-carcinoma transformation of QBD in the treatment of chronic liver diseases. PIK3CA and MAPK1 showed the most critical target effects.

### 3.4. GO Biological Function and KEGG Signal Pathway Enrichment Analysis

**Figure 5:**
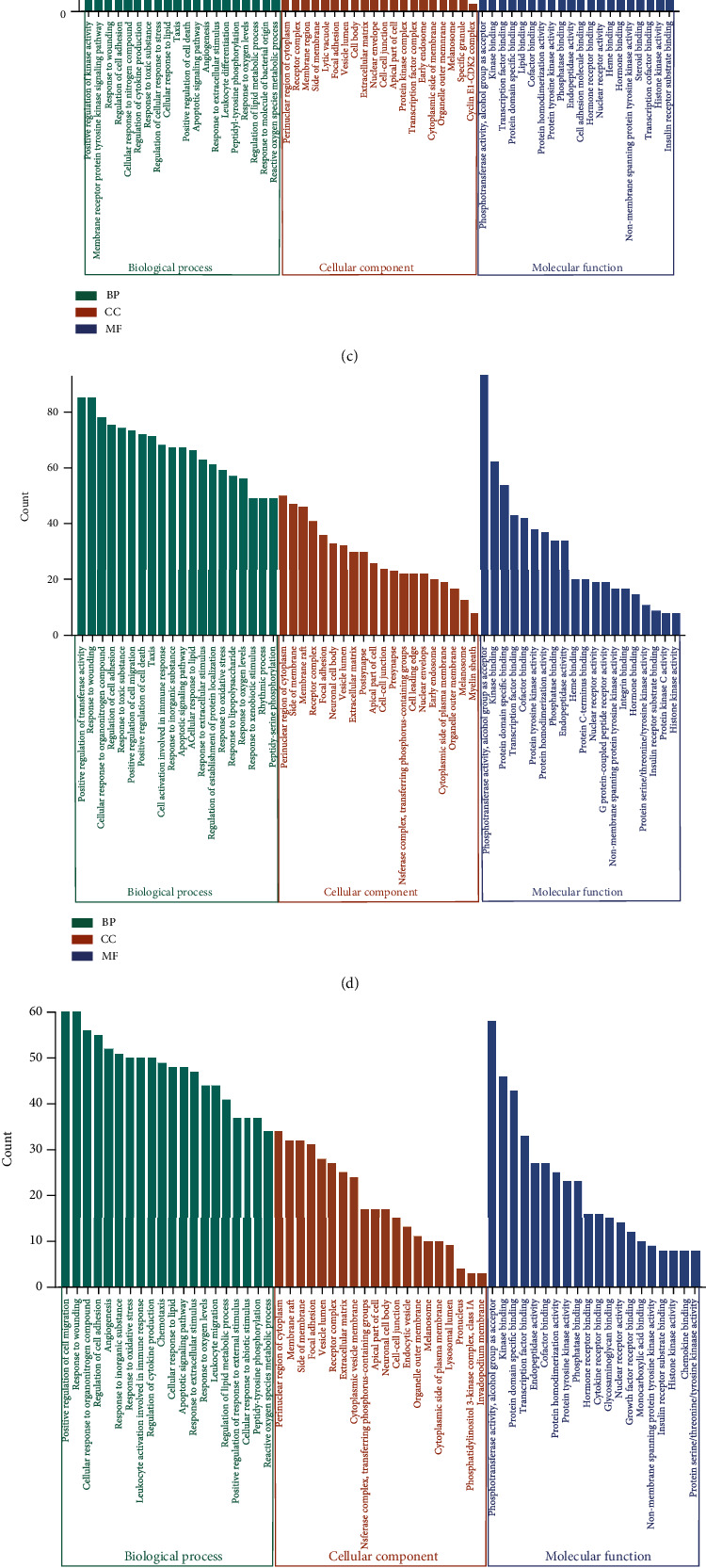
GO enrichment analysis graph for QBD intervention in CLD. (a) QBD intervention ALD (b) QBD intervention NAFLD (c) QBD intervention HB (d) QBD intervention HC (e) QBD intervention HF (f) QBD intervention LC and (g) QBD intervention HCC. Green for biological processes, orange for cell composition, purple for molecular functions (*P* value <0.01).

GO and KEGG enrichment of treatment targets was performed via Metascape, with Homo sapiens as the biological species and the threshold of *P* <0.01. Bioinformatics was used to visualize the first 20 pathways of enrichment results. The output GO enrichment bar graph showing the top 20 enrichment results of biological process (BP), cell composition (CC), and molecular function (MF) is represented in Figure 5.

**Figure 6:**
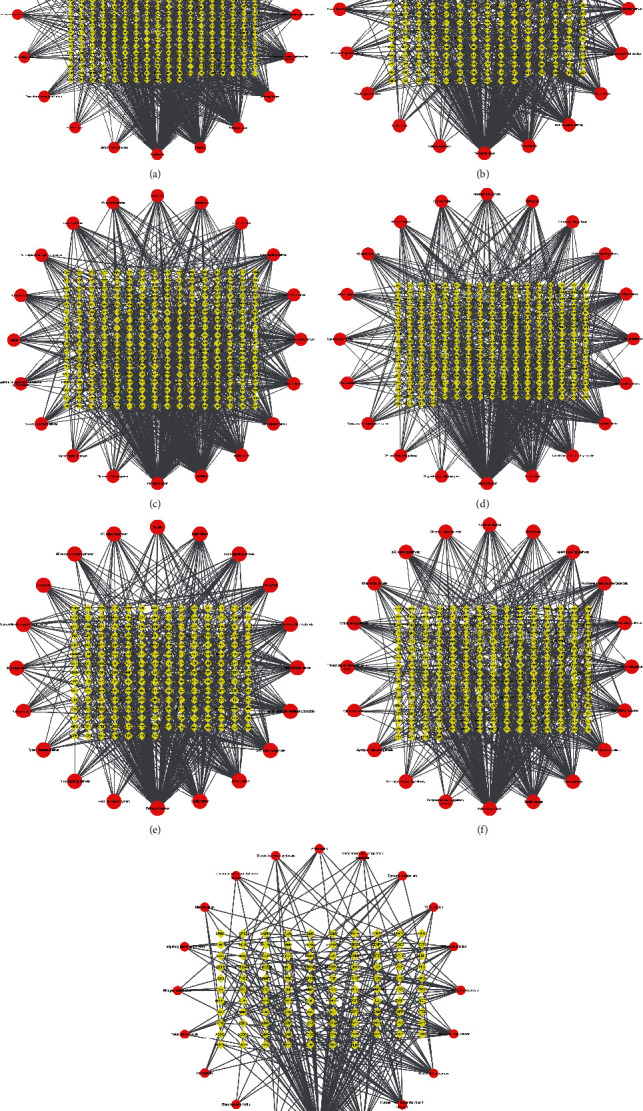
KEGG pathway-target network of QBD intervention in CLD. (a) QBD intervention ALD (b) QBD intervention NAFLD (c) QBD intervention HB (d) QBD intervention HC (e) QBD intervention HF (f) QBD intervention LC, and (g) QBD intervention HCC. The red circle represents the enriched KEGG pathway and the yellow diamond indicates the intersection target.

KEGG enrichment analysis was performed on the disease targets of seven chronic liver diseases, after which the DC of each enrichment pathway of the target set was calculated by Cytoscape 3.7.2. The first 20 pathways of the enrichment results of each disease were visualized to obtain the pathway target diagram, as shown in Figure 6.

**Figure 7:**
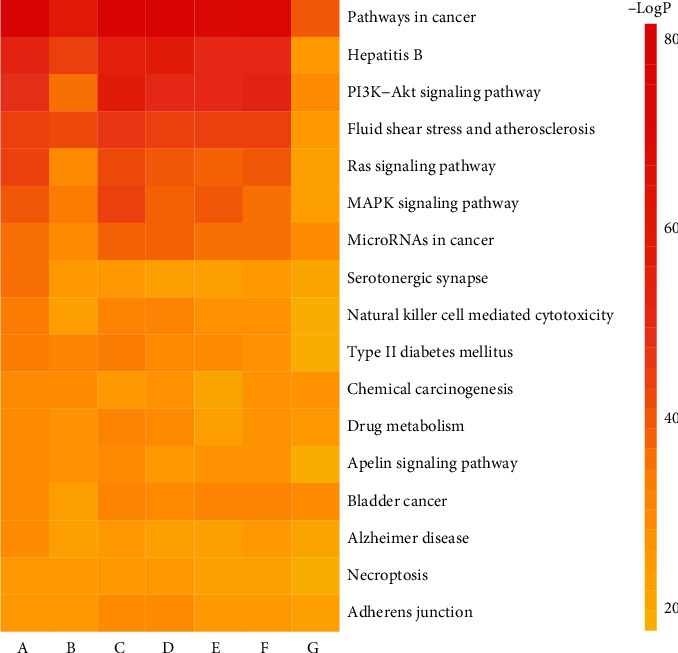
KEGG clustering heat map of QBD intervention CLD. A-G stands for ALD, NAFLD, HB, HC, HF, LC, and HCC, respectively.

Fourteen pathways, including pathways in cancer, microRNAs in cancer, bladder cancer, and other pathways, which ranked the highest among seven chronic liver diseases, were selected to output KEGG clustering heat maps (Figure 7). The corresponding pathway color changed from red to yellow with the increase of the *P* value, illustrating that the pathway types of the seven chronic liver diseases were similar. However, with the development of the inflammation-carcinoma transformation of liver diseases, the intervention intensity of QBD on pathways of chronic liver diseases was different in different periods.

### 3.5. Molecular Docking Network Analysis

According to the PPI network, there are two common key therapeutic targets, PIK3CA and MAPK1, in 7 chronic liver diseases (Figure 4). Two key targets from the drug-compound-target network **(**Figure 3**)** corresponded to the key active ingredients involved in QBD as dihydroartemisinin (DHA), artesunate (ART), 12-o-nicotinoylisolinolone, caffeic acid (CA), and diincarvilone A (Table 2). The secondary structure of the treatment target was selected through the UniProt database and PDB database. The secondary structure sequence numbers selected by PIK3CA and MAPK1 were 4JPS and 2OJG, respectively. Small-molecule compounds were docked with therapeutic targets, and the affinity between them was judged by calculating the total score of the result of the SURFLEX-dock module in Sybyl-X 2.0.0.

**Figure 8:**
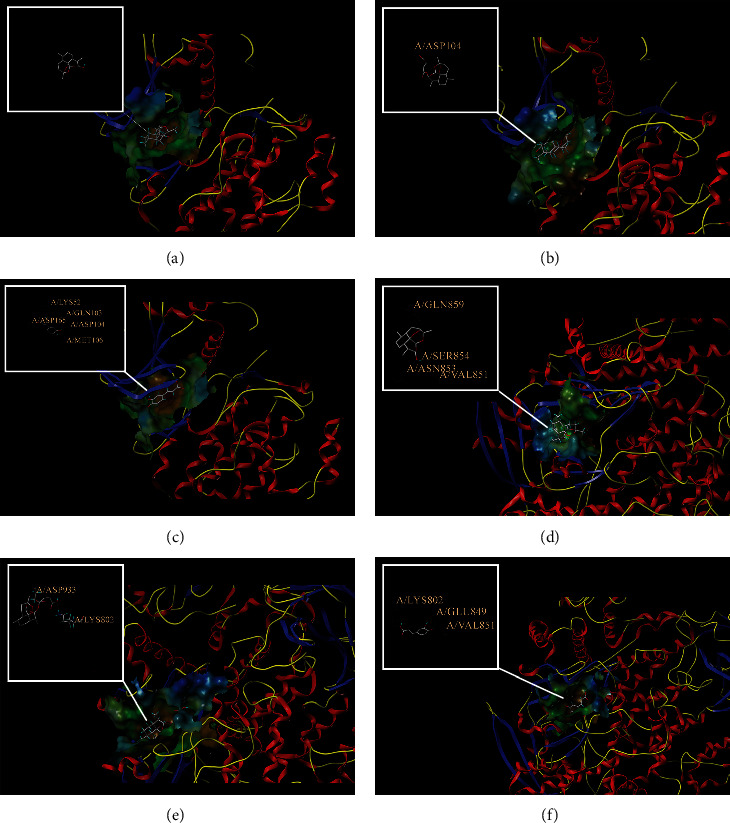
Analysis of drug-target protein interaction patterns. (a)-(c) Molecular docking of dihydroartemisinin, artesunate, caffeic acid, and MAPK1 protein. (d)-(f) Molecular docking of dihydroartemisinin, artesunate, caffeic acid, and PIK3CA protein.

The docking results of key targets and active components are shown in Table 3. Generally, it is believed that the Dock system docking fraction >4.25 indicates the existence of binding activity between the molecule and the target; docking fraction >5.0 indicates high binding activity, and docking fraction >7.0 indicates strong binding activity. The docking study showed that except for 12-O-nicotinoylisolinolone and diincarvilone A, the results of other compounds and targets were good. Small molecules enter the active center of the target protein. Molecular docking was carried out through the SURFLEX-dock. The hydrogen bond and ribbon diagram were drawn using the Define Protein view function, and the docking results were visualized. The secondary processing of molecular pockets and small-molecule compounds was performed with Adobe Photoshop CC 2018, as shown in Figure 8.

## 4. Discussion

This study aimed to clarify the molecular mechanism of QBD in the treatment of chronic liver diseases, find the important nodes in the inflammation-carcinoma transformation process of CLD, and analyze the relationship between key drugs and key targets in complex networks. Two key targets, MAPK1 and PIK3CA, were selected from seven chronic liver diseases treated by QBD. Both targets had important roles at the stage of the inflammation-carcinoma transformation process in chronic liver disease. The active components of MAPK1 and PIK3CA in QBD included DHA from AAH, ART from AAH, 12-O-nicotinoylisolineolone from ARR, CA from MC, and diincarvilone A from RR. The pathways involved in the seven chronic liver diseases were mainly pathway in cancer, hepatitis B, PI3K-AKT signaling pathway, Ras signaling pathway, and MAPK signaling pathway.

Previous studies have indicated that DHA, ART, and CA have an effect on chronic liver diseases. DHA and ART are two artemisinin derivatives isolated from *Artemisia annua*, a traditional Chinese herbal medicine that exerts strong anti-tumor activity in many tumor cell lines and has synergistic anti-tumor effects with other drugs [30]. DHA treats malaria by killing the parasites causing the disease. In addition to this prominent role, DHA can regulate different cell functions, such as angiogenesis, tumor cell growth, and immunity, as well as improve inflammatory diseases [31]. Reactive oxygen species (ROS) produced by endoperoxide bridges in DHA contribute to effective cytotoxicity in cancer cells [32]. Dr. Bai reported that oral DHA inhibited the progression of colitis-associated colorectal cancer by inhibiting macrophage-related inflammatory response in the early colitis-associated colorectal cancer and inhibiting late tumor cell growth. Moreover, early DHA treatment prevents carcinogenesis by inhibiting inflammatory response rather than its anti-tumor effect [33]. DHA exerts anti-inflammatory effect in hepatic fibrosis by inhibiting inflammation and the expression of pro-inflammatory cytokines in activated hepatic stellate cells (HSC) [34]. It has also been demonstrated that DHA has an important role in controlling excessive inflammation. DHA can also significantly improve alcoholic liver damage by inhibiting hepatic steatosis and improving liver histological lesions, inflammatory gene expression, and inflammatory cell infiltration induced by alcohol [35]. In addition, no serious side effects on human health have been reported, and DHA has been proven effective against various human cancers, including lung cancer, gastric cancer, and pancreatic cancer. DHA effectively inhibits the proliferation and migration of HepG2.2.15 cells *in vitro* and the growth of tumors *in vivo*. Previous studies have also shown that DHA may be effective in HBV-positive HCC patients and other types of HCC patients [36], leading to G2/M cell cycle arrest and apoptosis and significantly preventing the growth of HCC xenograft [37]. These studies suggested that DHA may be a promising therapeutic agent for the inflammation-carcinoma transformation process of chronic liver disease.

With other non-antimalarial properties, ART can regulate intestinal microbiota and reduce inflammation, thus lessening the liver and intestinal damage caused by CCl_4_, alcohol, and high fat diet [38]. Lee found that ART reduces Lipopolysaccharide (LPS)-induced inflammatory response in BV2 cells [39], protects mice from autoimmune hepatitis induced by concanavalin A by inhibiting the inflammatory response [40], and reduces hepatic fibrosis caused by various pathogenic factors and inflammation [41]. Moreover, ART nanoliposomes have anti-tumor effects on human hepatoma carcinoma cells *in vivo* and *in vitro* [42] and can promote cell apoptosis *in vitro* with selective cytotoxicity to tumor cells thus suggesting that ART may be an effective blocker of the inflammation-carcinoma transformation.

CA is a phenolic compound synthesized by plants in popular drugs such as coffee, wine, tea, and propolis, with anti-oxidant, anti-viral, anti-inflammatory, liver-protecting, and anti-tumor activities. *In vitro* and *in vivo* studies have shown that CA has anti-tumor activity against HCC. It has also been reported that the anti-tumor characteristics are related to the anti-oxidant and pro-antioxidant effects. The anti-HCC effect is related to preventing ROS production, inducing DNA oxidation of cancer cells, reducing angiogenesis of tumor cells, blocking stats, and inhibiting MMP2 and MMP9 [43]. Furthermore, CA inhibits LPS-stimulated inflammatory responses by blocking NF-*κ*B and MAPK activation in macrophages without causing hepatotoxicity at concentrations with strong anti-inflammatory potential [44]. Dr. Pang found that inhibition of ERK1/2-mediated Egr1 transcriptional activation by CA reduces hepatotoxicity [45] and activates the anti-oxidant defense system to prevent acetaminophen-induced liver injury [46], which suggests that CA could be used as a natural source of the safe liver-protecting drug.

MAPK1 (Mitogen-activated protein kinase 1) also known as ERK2(Extracellular signal-regulated kinase 2), is a member of the MAPK family and the core signal transductor of the MAPK/ERK pathway [47], whose cascade is controlled by RAF, MEK1/2 and ERK1/2 protein. MAPK signaling pathway is a process of cascade phosphorylation, the core members of which include: MAP kinase kinase kinase (MAPKKK), MAP kinase kinase (MAPKK) and MAP kinase (MAPK). The MAPK/ERK pathway has a central role in many cell physiological processes, including cell cycle, proliferation, growth, and apoptosis. There is sufficient evidence indicating that this pathway is over-activated in various types of cancer, including HCC that is usually related to overexpression and over-activation of ERK1/2 and results in high proliferation, growth, and invasion of tumor cells. Consequently, ERK is considered as the strategic target for hepatocellular carcinoma [48]. Previous studies have also proven that the activation (phosphorylation) of ERK and the expression of fibrosis markers are inhibited by inhibiting IL-11, which reduces hepatocyte death and liver fibrosis, inflammation, and steatosis in non-alcoholic steatohepatitis models [49]. Moreover, it was found that down-regulation of RAF kinase inhibitor protein promotes ERK signal transduction, and ERK phosphorylation directly regulates downstream gene expression, thus leading to severe hepatic fibrosis. Alteration of the ERK signaling pathway via knockout of ERK2 can reduce hepatic fibrosis and inflammatory response [50]. Moreover, in patients with liver cirrhosis and HCC, the overexpression of Raf, MEK, and ERK has also been detected [51], suggesting the overactivation of ERK in hepatic fibrosis, liver cirrhosis, and HCC. It has also been found that simultaneous activation of the ERK and AKT(Serine/threonine kinase) pathways enhances the cell cycle progression of HBV replicating hepatocytes [50] and that the ERK pathway participates in HBX(Hepatitis B virus X protein)-mediated HCC cell proliferation and migration [52]. The secretions of HSCs also promote the proliferation of HCC via the ERK pathway [53]. ERK substrate, Egr1, has been found to promote angiogenesis, fibrillogenesis, and tumorigenesis in HCC. Inhibition of the MAPK signaling pathway reduces cell proliferation and the expression of tumor stem cells and increases apoptosis of hepatoma carcinoma cells [50]. All these studies indicate that ERK2 has a very important role in the development and progression of the inflammation-carcinoma transformation process of chronic liver disease, which can be inhibited by impeding the activity of MAPK1.

PI3K (phosphatidylinositol 3-kinase) is a phospholipid kinase family, classified in Type IA, IB, II, and III based on structural characteristics. Class IA PI3K is a catalytic subunit (p110*α*, p110*β*, or p110*δ*, encoded by PIK3CA, PIK3CB, and PIK3CD genes, respectively) and a regulatory subunit (p85*α*, p50*α*, p55*α*, p85*β* or P55*γ* encoded by PIK3R1, PIK3R2, and PIK3R3, respectively). The key genes involved in the PI3K/AKT signaling pathway are PI3K and AKT, so this pathway is directly named after these two genes. Class IA PI3K can activate downstream signals, such as AKT, having an effect on important cellular signal transduction pathways, including growth, metabolism, migration, proliferation, and survival. The genome changes of the PI3K signaling pathway have been associated with many different tumors. The most common cancer-related alterations in the PI3K pathway are mutations activating PIK3CA-encoding p110*α*. Besides, the PIK3CA mutations have also been found in some non-malignant diseases [54]. The PI3K/AKT signaling pathway has a very important role in coordinating pro- and anti-inflammatory pathways, with effective immunity when protecting host tissues [55]. Dr. Liu found that scoparone improves liver inflammation and autophagy by inhibiting the PI3K/AKT/mTOR pathway in mice with non-alcoholic steatohepatitis [56]. Other studies have reported that salvianolic acid A inhibits liver function, hepatic fibrosis index, and collagen deposition by inhibiting the PI3K/AKT/mTOR signal cascade and alleviates the hepatic fibrosis by improving hepatic fibrosis degree [57]. Currently, it is believed that PIK3CA hotspot mutation is helpful in the PIK3CA pathway disorder in some hepatobiliary carcinomas [58]. HBV core protein promotes tumorigenesis by activating the Src/PI3K/AKT signaling pathway [59]. All the above studies indicate that PIK3CA may participate in the inflammation-carcinoma transformation process of chronic liver disease.

The interaction between MAPK and the PI3K/AKT pathways has been found in skin melanoma. Interestingly, all these studies indicate that the two pathways are activated in parallel and describe the evidence of a correlation between PI3K/AKT and MAPK/ERK1/2 cascades. There are multiple intersections between these two pathways, and their synergies determine the outcomes of cells. The PI3K/AKT and MAPK pathways interact at different stages of signal transmission [60]. Zhuang found that PI3K inhibition blocks capsaicin-induced ERK activation. PI3K is involved in the early events (beginning) of inflammatory hyperalgesia, while ERK participates in the late events (maintenance) [61]. PI3K was found to promote Rac activation and further stimulate PAK that can phosphorylate MEK and may stabilize MEK interaction with Raf [62]. A few studies indicated that the PI3K/AKT/mTOR pathway was activated in about 50% of HCC patients. Long-term use of ERK/MAPK inhibitor treating HCC often leads to reverse activation of the PI3K/AKT pathway, resulting in drug resistance. For example, sorafenib has been proven to activate the PI3K/Akt pathway in HCC cells, and the over-activation has been identified as one of the resistance mechanisms to sorafenib [63]. Thus, inhibition of the PI3K/AKT/mTOR pathway may be a promising therapeutic option for overcoming resistance to inhibition of the MAPK pathway [64].

**Figure 9:**
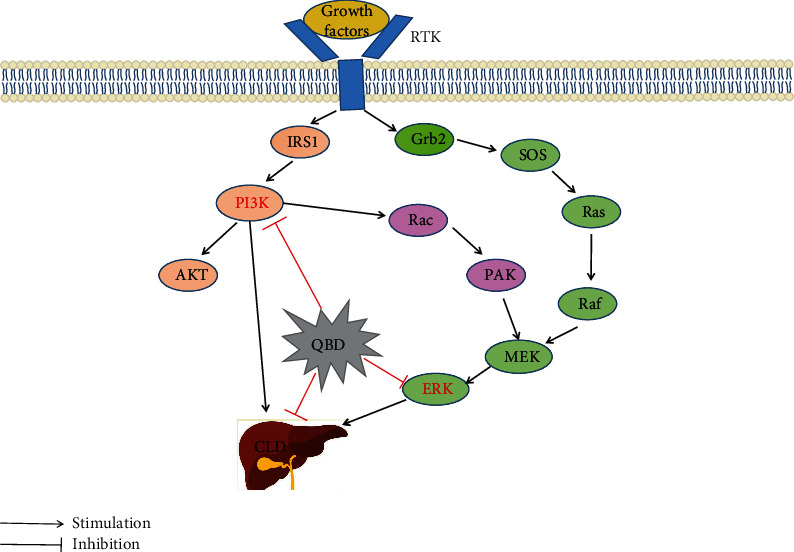
The possible mechanism of QBD in the treatment of CLD.

All of the above evidence indicate that MAPK1 and PIK3CA may participate in the inflammation-carcinoma transformation process of chronic liver disease (Figure 9). The development and progression of liver cancer involve multiple procedures, many of which have a synergistic effect. Through a series of phosphorylation events in the MAPK and PI3K/Akt pathways, downstream signals may activate transcription factors to change the expression of proteins in important cellular processes (such as proliferation and differentiation), apoptosis, cell cycle process, tumorigenesis, tumor growth and angiogenesis [65]. MAPK and PI3K/Akt pathways may represent a promising therapeutic target or otherwise cause destructive diseases. They may be activated in parallel in some diseases and interact with each other. Inhibiting one pathway may activate the other; thus, it is necessary to treat diseases by strategically targeting the two pathways. Some active components in TCM may have a role in HCC by strategically regulating the PI3K/AKT/MAPK pathway. For example, Paris saponin VII reverses the drug resistance of HCC HepG-2/ADR cells by inhibiting the PI3K/AKT/MAPK signaling pathway [66]. Verbasine inhibits proliferation, migration, invasion, and apoptosis of HCC cells through down-regulation of PI3K/AKT and p38 and ERK/MAPK pathways [67]. In the present study, we explored the mechanism of QBD intervention on the inflammation-carcinoma transformation process of chronic liver disease via MAPK and PI3K/AKT pathway based on network pharmacology and molecular docking.

## 5. Conclusion

By using network pharmacology and molecular docking technique, we revealed the effect of QBD with DHA, ART, and CA as the active ingredients, MAPK1 and PI3K as the important targets, and the important pathways of MAPK and PI3K/AKT on the intervention on the inflammation-carcinoma transformation process of chronic liver disease. These findings provide a research direction for investigating the mechanism underlying the inflammation-carcinoma transformation process in QBD for chronic liver disease, identifying potential therapeutic targets, and preventing the transformation from non-controllable inflammation to cancer. However, our conclusions are based on theoretical simulations, so more experiments are needed to further verify our findings.

## Figures and Tables

**Table 1:** CLD search terms and number of target genes in each database.

Search term	Genecards	OMIM	DisGeNet	TTD	Total number of target
Alcoholic liver diseases	1848	146	182	0	2176
Non-alcoholic fatty liver disease	1209	149	959	3	2320
Hepatitis B	1335	196	1249	7	2787
Hepatitis C	1162	190	1591	10	2953
Hepatic fibrosis	1272	27	58	0	1357
Liver cirrhosis	958	1182	1045	1	2039

**Table 2:** Parameters related to molecular docking compounds.

Number	PubChem CID	Compound name	Related herb	OB(%)	DL	Structure
1	3000518	Dihydroartemisinin	AAH	50.75	0.30	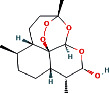
2	6917864	Artesunate	AAH	15.89	0.65	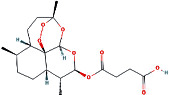
3	5320138	12-O-Nicotinoylisolineolone	ARR	20.70	0.83	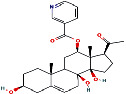
4	689043	Caffeic acid	MC	25.76	0.05	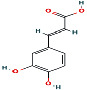
5	60155233	Diincarvilone A	RR	3.14	0.38	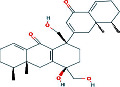

**Table 3:** Score for molecular docking of key targets and key active components.

Number	Mol id	Compound name	Target name	Total score
1	MOL007425	Dihydroartemisinin	MAPK1	5.0833
2	MOL007434	Artesunate	MAPK1	7.0549
3	MOL004498	12-O-Nicotinoylisolineolone	MAPK1	0.2795
4	MOL000223	Caffeic acid	MAPK1	5.095
5	MOL003734	Diincarvilone A	MAPK1	-1.0452
6	MOL007425	Dihydroartemisinin	PIK3CA	3.6355
7	MOL007434	Artesunate	PIK3CA	5.5662
8	MOL004498	12-O-Nicotinoylisolineolone	PIK3CA	-1.5526
9	MOL000223	Caffeic acid	PIK3CA	5.2177
10	MOL003734	Diincarvilone A	PIK3CA	-7.891

## Data Availability

The data of our work can be acquired from the Supplementary Materials uploaded with this article.

## Abbreviations

AAH: Qinghao
ADME: Absorption/distribution/metabolism/excretion
AKT: Serine/threonine kinase
ALD: Alcoholic liver disease
ARR: Zhimu
ART: Artesunate
BP: Biological process
CA: Caffeic acid
CC: Cell composition
CLD: Chronic liver disease
DC: Degree centrality
DHA: Dihydroartemisinin
DL: Drug-likeness
ERK: Extracellular signal-regulated kinase
GO: Gene ontology
HBV: Hepatitis B virus
HBX: HepatitisB virus X protein
HCC: Hepatocellular carcinoma
HCV: Hepatitis B virus
HF: Hepatic fibrosis
HSC: Hepatic stellate cells
KEGG: Kyoto encyclopedia of genes and genomes
LC: Liver cirrhosis
LPS: Lipopolysaccharide
MAPK: Mitogen-activated protein kinase
MC: Mudanpi
MF: Molecular function
NAFLD: Non-alcoholic fatty liver disease
OB: Oral bioavailability
PDB: Protein data bank
PI3K: Phosphoinositide-3 kinase
PPI: Protein-protein interaction
QBD: Qinghao Biejia decoction
ROS: Reactive oxygen species
RR: Shengdihuang
SFXC: SURFLEX-dock
TC: Biejia
TCM: Traditional Chinese medicine
TCMID: Traditional Chinese medicines integrated database
TCMSP: Traditional Chinese medicine systems pharmacology database and analysis platform.

## Supplementary Materials

Supplementary Materials Supplementary data associated with this article can be found in the appendix. Supplementary file 1 shows the compounds and their associated targets for QBD and Supplementary files 2-8 show the associated targets for CLD.
