# Supplementary material for: Network Pharmacology-Based Exploration on the Intervention of Qinghao Biejia Decoction on the Inflammation-Carcinoma Transformation Process of Chronic Liver Disease via MAPK and PI3k/AKT Pathway
Source: Biomed Res Int. 2022 Oct 14;2022:9202128. doi: 10.1155/2022/9202128 (PMC9586778; doi:10.1155/2022/9202128)
Supplement: Supplementary Materials — Supplementary data associated with this article can be found in the appendix. Supplementary file 1 shows the compounds and their associated targets for QBD and Supplementary files 2-8 show the associated targets for CLD. [file 9202128.f1.zip › ALD target.pdf]

after deleting duplicate targets）
TNF
IL6
IL1B
INS
GGT1
HFE
SLC17A5
IL10
TP53
TLR4
F2
PPARG
TGFB1
ALDH2
PKHD1
PKD1
APOE
PNPLA3
AKT1
FAS
ALB
NR1H4
LEP
APOB
CD40LG
STAT3
ADH1C
BDNF
AFP
MTHFR
GPT
IFNG
CCR6
SNCA
SERPINA1
ADH7
VEGFA
TLR2
ACE
CAT
TF
CTNNB1
IGF1
DRD4
CCL2
KRAS
INSR
HLA-DRB1
HGF
CFTR
APOA1
APC
CYP2E1
SEC63
CTLA4
ABCB11
COMT

IRS1
PKD2
EGFR
G6PC
PIK3CA
CRP
SLC6A3
LEPR
PTEN
CDKN2A
SOD2
GBA
SLC6A4
LRP5
MTOR
HRAS
HMOX1
STAT1
FASLG
TTR
MYC
RETN
CP
SERPINE1
HNF4A
RBP4
IGF2
ATP7B
ICAM1
NPC1
LPL
IL1RN
ABCB4
JAG1
APP
CXCL8
NOTCH1
MARS1
TNFRSF1A
POLG
CCND1
NOS3
PRKCSH
EPO
MPO
RELA
MMP2
GFAP
GRIN2B
HLA-B
ABCB7
MTTP
DDIT3
CDH1
ABCB1
EGF
HAMP
MAPK1

HNF1A
FOXP3
CASP8
SQSTM1
TERT
ERBB2
MLXIPL
NR1H3
GJB1
SMAD4
KIT
IRS2
POMC
VWF
ABCA1
PPARGC1A
HADHA
HTR2A
LMNA
NOD2
SMPD1
ESR1
MAPT
TLR3
PON1
PRKN
ADIPOQ
SLC2A1
ABCC2
CRH
SERPINC1
MET
NFKB1
KRT18
MMP1
COG2
AGL
AHSG
GATA4
TYMP
GAA
GNG13
JAK2
PTPN11
BRAF
REN
PRNP
MUC1
SPP1
TH
BRCA1
IGF2R
IGF1R
CAV1
ELANE
CXCR4
NDUFS1
HSPB1

PIK3R1
FBN1
NOS2
F5
CASP3
ERCC6
IL2RA
PSEN1
HNF1B
NGF
LDLR
TSC2
HLA-DQB1
CYP3A4
MFN2
AR
VIM
EDN1
ENG
TGFBR2
AKR1A1
ATP8B1
BAX
TMEM67
LRRK2
PSAP
GANAB
MAOA
GRIN2A
B2M
CXCL12
SHH
FGFR1
RAF1
SERPINA3
CD36
MME
BMP6
MPZ
GABRA2
CPT2
HLA-A
FAH
CCN2
TNFRSF1B
CDKN1A
PRODH
NFE2L2
GHRL
PPARA
OTC
SRC
PTPRC
BRCA2
LCAT
CYP2D6
ELN
SOCS3

GBE1
NRAS
UCHL1
XIAP
MDM2
IL13
CDKN1B
SLC25A13
PDGFRB
TRPV4
CCR5
UGT1A1
MSH2
NPC2
GDNF
FLT1
FGFR2
TCF4
RET
DNMT3B
THBD
IKBKB
TGFB2
ACTB
TSC1
IL12RB1
NDUFS8
IL1A
MMP9
PROM1
GLA
BCL2
CD4
SMAD3
MBL2
CETP
SEPSECS
PLAU
CDK4
VCP
NFKBIA
MT-CO1
RB1
HBB
PRTN3
FN1
ADAM17
FECH
TNFRSF11B
TNFSF11
SYP
RARB
IL2
STK11
CDKN2B
GABRG2
PDGFRA
CDKN3

NPM1
ACP5
RYR1
GPC3
MAP2K1
G6PD
DGUOK
TGFBR1
THPO
TFR2
GAL
MMP3
ERBB3
CST3
HJV
EPCAM
APOC3
UROD
ACADM
AGT
PLG
IL4
MT-CYB
EZH2
HLA-DQA1
ADAMTS13
F8
MAPK8
AGTR1
AKT2
SLC40A1
ARG1
IL18
DMD
TGFB3
CTSB
ASS1
NLRP3
ADA
MECP2
SPINK1
ATM
CYBB
CRYAA
CFH
DKK1
ALG8
ARSA
CD40
MT-ATP6
MPV17
ABCG2
EIF2AK3
CTSD
GCK
DNAJB11
HTT
PTCH1

WT1
NPPA
MLH1
YAP1
CREBBP
KRT8
AIRE
AVP
TGFA
SLC2A4
UMOD
PYGM
MT-ND1
TWNK
CCL3
LTA
NPHP1
ITGAM
PTH
MT-ND4
AKR1D1
PDCD1
NF1
HMBS
BMP2
CASR
SMARCA4
VEGFC
ATXN2
CC2D2A
MMP14
SIRT1
LPA
ENPP4
CEBPA
CD81
U2AF1
CALR
JUN
PTGS2
PSEN2
SLC11A2
NR3C1
NOTCH2
TJP2
SLC1A2
HCRT
MGMT
SREBF1
SOS1
BCS1L
CDK6
INVS
TIMP3
SLC18A2
ALAD
CHAT
HSD3B7

GSTM1
TNFRSF11A
IL2RB
CPOX
IL17A
AKT3
CYCS
LIPC
AXIN1
FIG4
PINK1
TARDBP
DPYD
CYP1A1
TRMU
ALAS2
IDUA
TWIST1
MEFV
CYP1A2
CDKN1C
ALPL
IDH2
PKLR
SMARCB1
GSR
PLA2G6
GRN
TIMP1
CDH2
STEAP3
TM7SF2
WRAP53
TBK1
F9
CREB1
KITLG
MKS1
MVK
PARK7
ASAH1
IFIH1
CYP27A1
SLC10A2
FOS
CFI
CHRNA3
ACADVL
FARSB
GAPDH
FGF23
PANK2
CHUK
TYR
KRT7
SPARC
ZEB2
POLG2

FGFR3
RRM2B
ENPP1
HP
CACNA1A
NTRK1
TP73
ERBB4
UROS
PMM2
LOX
LRP6
GARS1
SST
GJA1
PYGL
STAT5B
SBF2
BCHE
ALDOB
SMARCA2
DLD
PHKA2
RAC1
CYP19A1
CYP2C9
MAT1A
ACTG1
WNT1
FBP1
EDNRB
CYP2C19
PRKAR1A
MIF
XDH
SNAI2
COL11A1
FLNA
AARS1
TTN
PRSS1
ABCC8
TMPRSS6
L1CAM
GH1
ASL
ACTC1
TCF7L2
KRT19
PLP1
ZEB1
PIK3R2
LIPA
NHLRC1
F7
GNMT
PRKCD
SDHB

FXN
FGFR4
EPM2A
GLUL
SMAD7
KIF1B
AURKA
ARID1A
HIF1A
EDNRA
GYS2
PDGFB
OPRM1
XBP1
SI
PAH
APOA5
SOX2
ESR2
EPAS1
RPGRIP1L
SOX9
PRX
RAD51
CAV3
PEX6
UCP2
NOTCH3
FTL
ALDH9A1
DVL1
HMGCR
EP300
ETFDH
F3
ADRB2
HSPD1
TNFRSF10B
MEN1
GATA1
KNG1
DNM2
HLA-C
IKBKG
TFRC
NTRK2
CYP2A6
GSTP1
CCL5
CA2
EZR
PIP5K1C
HEXA
XRCC1
PIK3C2A
SLC30A10
FGA
HADHB

MSR1
DCTN1
DBH
SCN5A
FGF2
ARID1B
MT-CO2
PMP22
VDR
NAGA
KCNQ1
KRT5
DARS2
MTMR2
IFT88
MECOM
FASN
PIK3CD
COX5A
PC
HSD17B10
RUNX2
KCNJ11
PAX2
IFNL3
WNT3A
PNKD
SETX
SLC25A4
NEFH
IFNA2
FGF21
SERPINF2
ALMS1
OGG1
ALOX5
GSK3B
FABP1
SOX10
TREX1
WNT4
NPY
ETS1
GALNS
ACADS
KL
CCL11
DNM1L
TM6SF2
AXIN2
NR3C2
SLC6A2
TNFSF12
TLR9
CDC42
ATF4
CSF1R
MTR

CACNA1C
GABBR2
SFTPC
C4A
ABCA4
AQP2
CXCR1
ABCG8
CYP17A1
[illegible]NRNPA2B1
SPG11
FMR1
BSCL2
BIRC3
TP63
HSPG2
NRG1
IL7
WNT5A
CALCA
PRL
CHRM3
ATP13A2
RAB7A
GDAP1
NAGS
CHI3L1
IFNB1
MAP3K5
ATRX
TNFSF10
COX6B1
NAGLU
OGDH
MAPK3
ATF6
INPP5E
HSPA4
FADD
MT-ND6
DHFR
SH3TC2
OPTN
PODXL
MMP13
NPPB
PON2
S100B
BTD
OFD1
MT-ND2
BMP4
GLI1
CLDN1
HK1
IL5
DDX58
TNNI3

CYP27B1
IL6R
CD27
CXCL10
WNT3
MAPK10
RAG2
ADH1A
TNFRSF13B
GUSB
AHCY
ITGB2
CLCN3
CYP7B1
FUS
SCP2
MMP8
MAG
KDR
TNNT2
SCN1A
KMT2A
MSH6
BGLAP
DIABLO
CYP1B1
AGPAT2
SLC4A1
ATP7A
BMPR2
INF2
PMS2
PITX2
ERCC1
CD44
PSMB8
SLC39A8
ERCC2
CYP7A1
RNASEH2C
GALC
PCSK9
CEL
ADAR
FLT3
ADIPOR1
RHO
HTR1A
REST
GATA3
GATA6
ABL1
CTCF
PHKG2
NPHP3
HTR3A
MAPK14
CSF3

NEFL
VHL
SAA1
KDM4C
NKX2-1
PNLIP
EDN3
GNRH1
ABCG5
DMPK
FGF3
CTSA
HSPA5
CNR1
CPS1
ERCC4
SCT
HSPB8
EIF2S1
PAX6
CHEK2
CHD7
ALPP
ATXN3
CD34
PDGFRL
PRKAG2
NKX2-5
MTUS1
DLC1
PIEZO1
SGCE
PTHLH
TYK2
DES
ADIPOR2
AVPR2
GCG
SLC39A14
PRKAA1
MRAP
PRRT2
CEP290
TOR1A
ACSL4
KLF6
GLS
ALK
CEACAM5
SLC25A15
CAMTA1
LRP1
PCCA
PDX1
DICER1
HPRT1
NPHP4
CNTNAP2

GATA2
KRT14
GSN
NQO1
ETFA
EPOR
SETD2
SLPI
SCD
SCN8A
CASP9
IGFBP3
SURF1
XK
HSP90AA1
IL18BP
MYH7
HEPACAM
FRZB
GLUD1
MYH9
CD8A
CCK
BCL6
WNT7A
TALDO1
HUWE1
IL1R1
SUOX
GM2A
ELOVL4
IL10RB
DDX3X
DKC1
ACHE
OCLN
SREBF2
MYCN
RARA
HOXA13
SET
NGLY1
TBP
DEPDC5
PPOX
SGSH
DPP4
LIG4
APOH
CDK1
IGFBP1
CHIT1
CD19
LAMP2
DACT1
CR1
ACACA
MTM1

CD28
BCL2L1
HSD17B4
ATXN1
FABP4
PRKAB1
KRT17
JAK1
BMPR1A
APOA2
BLK
APPL1
FTH1
NAT2
ALS2
SPRTN
CD274
C3
ITGB3
DYNC1H1
PLAT
ARID2
TMEM216
YY1AP1
MYD88
SELE
SOST
SAMHD1
SUCLG1
FLT4
SUMF1
FH
F10
IL17F
RPS6KB1
CRYAB
CDK2
DVL3
PON3
MAP2K2
EGR1
MITF
TEK
TTC21B
LIN28B
SELP
SRA1
HDAC6
HTRA2
IL7R
PML
GCKR
SLC22A5
CSF2
IRF1
GABRB2
NUS1
PRF1

BIRC5
DCDC2
LCN2
HAVCR2
HLA-DPB1
BCKDHB
CPT1A
NBN
ODC1
IREB2
RARS1
ECE1
DPP6
PRPS1
WNT7B
HSD11B1
VCAM1
ANO5
ARSB
ANXA5
WDR19
GNE
TIMP2
XRCC4
NR1H2
SLC2A2
HEXB
AMH
CASP1
FTO
NBAS
HARS1
IGF2BP2
VPS37A
KRT13
TTPA
GALE
LARS1
MKI67
ICOSLG
CD80
SLC26A4
CAPN3
TPH1
HADH
BCL11A
EPHX1
PLD3
POMGNT1
PDYN
GRM5
CRHR1
ETV6
ENO2
NR1I3
FAR1
TG
DLL4

PVALB
SLCO1B3
MAP3K20
VAPB
ADH5
CD79A
PIK3CG
TSPO
ETFB
PLA2G7
RUNX1
RPGRIP1
FLNC
DAG1
SHBG
PAPOLG
AKR1B1
CYBA
RXRA
SERPINI1
GGT2
FKRP
APTX
TINF2
TKT
GDF2
STIM1
CLU
ALDH1A1
SHC1
SFTPB
TUBB
NEK8
CCNA2
LRP2
PTPN3
GRIN1
TLR5
SLC7A7
PRDM16
YARS1
GOLM1
SLC19A2
AHR
NR1I2
CTSC
PLAUR
VPS13A
PLA2G2A
CALB2
NTRK3
COG7
SP1
DYNC2H1
NF2
SCN2A
ABCC3
MBP

RNASEH2A
CSF1
PRMT7
HTRA1
SLCO1B1
PITX3
HTR2C
GLB1
PEX5
RPS27A
SLC33A1
FGF8
SLC11A1
OPA3
CBL
TLR1
GAB1
TK2
SAMD9L
IL17RA
LRAT
AARS2
MSTN
CSTB
CBS
FZD2
SLC46A1
NR5A1
RHOA
NPHS2
IGHMBP2
FOXO1
DOCK8
CTSK
MCL1
ITGB1
ADRB3
FCGR2A
MYLK
KARS1
NEUROD1
WFS1
HGD
RNASEH2B
TBX5
CASP10
LITAF
GFER
SLC22A4
ADH6
SLC12A6
FIP1L1
PALB2
AGRN
RAB27A
VMA21
KIF1A
NR4A2

PGM1
BCOR
VIP
BTK
WDR45
SLC10A1
LIPE
ISG15
NPHS1
TBX1
MMP7
BBS4
ACOX1
TFAM
PEX1
SEMA3A
IL12B
ACTA1
NR0B2
TICAM1
GRB2
CYP11B2
CLDN10
HNRNPA1
PEX14
ACTN4
PECAM1
BAP1
ABCD1
MPL
HBG2
DBT
ADA2
ZAP70
SDHC
DPAGT1
NRXN1
EGR2
IFNGR1
GCH1
PEX19
PHEX
GSTT1
CASP7
GPC1
FANCD2
AMACR
SDCCAG8
SLC35A2
PTK2
COG5
DCN
CHRNA4
POMT1
HSPA8
C19orf12
PLA2G4A
SLC1A1

COX15
NCF2
HTR1B
NCAM1
CLPB
DNAJC13
GABRB3
GRM1
UBE3A
SNAI1
GABRA1
GAST
ABCA3
FAN1
ALDH3A2
PCK1
USH2A
GLI3
ACVRL1
CTNNA1
BBS2
PPIG
ANGPT2
PRKACA
GGTLC3
PRKCA
FLVCR1
FGD4
SOCS1
CDH23
VKORC1
TCTN2
ABCC1
IRF9
CD55
NAMPT
BBS1
FYN
CD14
TLR6
SLC52A3
IL23R
STAT6
B9D1
MRE11
CX3CR1
CLPX
SH2D1A
HMGB1
SLC27A5
ANKK1
GPX1
SMAD2
NTF4
TFAP2A
ENO1
ITGB4
PCNA

HDAC1
IL3
NOS1
LCT
PAX3
OPA1
CDKL5
DCAF8
SBDS
FGF10
NFKB2
DDC
JUP
CR2
GRIA3
GABBR1
TET2
ENTPD1
BEST1
CHCHD2
SLC39A4
RASSF1
ABCC6
ACAN
PNPLA2
PAX4
CDH3
ITGAL
CIITA
STXBP1
OLR1
OTX2
ADD1
IQCB1
SLC25A19
IFT80
MB
STS
CCNB1
TPM2
COG8
SELL
WWOX
TNFAIP3
COL9A3
GJC2
F13A1
PTRH2
MST1
ADM
ANGPT1
UBB
ALDH3A1
APOC2
CLCN1
C2CD3
CSF3R
TMEM231

DCLRE1C
ALDH18A1
BECN1
AHI1
CD46
GHR
GAD1
MUSK
MUC5AC
NDRG1
DAO
EPX
JAK3
CDK5RAP2
DNASE1
AMPD1
PEX16
POU5F1
DLAT
CNBP
PTH1R
SMAD6
C5
SCARB2
TPH2
PIK3CB
COX4I1
CALM1
GLP1R
PHKB
BIN1
AREG
SPTAN1
ERN1
AGTR2
PCK2
NSD1
DHCR7
SACS
CBLIF
FKTN
FANCA
RAD51C
SLC19A3
ITGA6
NEU1
GABRD
STAT4
AKR1C4
SCARB1
S100A9
NUP107
PTF1A
DOK7
FLNB
MPI
USP18
FOXL2

ACAD9
SLC34A3
MOGS
FOXF1
PGR
TRMT10C
CCL20
TPMT
NR0B1
CAPN1
RBPJ
PTGS1
NTF3
FGF19
CCL4
IGHE
ATCAY
PERCC1
TNFRSF10A
IFT122
EPHB4
COG6
CACNA1S
CASK
IL2RG
HLA-DPA1
ATG5
ADNP
PSENEN
KLF11
SLC20A2
EYA1
HSP90B1
IRF8
DDX41
PCDH15
LIMK1
SYNJ1
ACO2
KCNQ2
TRAF3
MED12
MT-CO3
FUCA1
CEP164
TSHR
P4HB
ATP5F1A
CLCN2
PLCB1
ABCB6
CDH17
OXT
AKR1C2
NOTCH4
GDF15
TPM3
PRKCB

DPM1
HLA-G
FOXC1
CHEK1
GLI2
SERPINA6
SLC25A22
CTC1
MGAM
GYG1
TAT
SNX14
FANCC
P2RX7
HSD11B2
SLC13A5
GJB2
RELN
NCSTN
AIP
SLC9A3R1
KCNJ10
ADK
CFHR5
OGT
SPTBN2
SERPINB3
ARVCF
RAPSN
ITCH
KLRK1
MTRR
BHMT
DNAH8
POLR1D
M6PR
EIF2AK2
PLCG1
FANCG
IFNAR2
TPM1
SIL1
NTS
BAD
ATR
PHB
PRKAA2
ABCD3
FANCF
MGP
IFI27
PAFAH1B1
RLBP1
ALDOA
PICALM
LRPPRC
VARS2
CXCL9

NUP133
VCL
FHL1
MYOC
BSG
RNASE3
RAP1A
RFXANK
CFHR1
GUCY2D
CYP3A5
MT-ND3
PKD1L1
MSX1
B9D2
EPRS1
UQCRC2
PNPO
GC
MRPS7
CYC1
IKZF1
MYBPC3
BAK1
HAX1
TUBB2A
AGER
ATP6V0A2
UBD
NR5A2
ITGA3
ARSH
ERCC5
TOP2A
TYMS
C9orf72
HDAC9
TBC1D24
ANXA2
MATN3
TBX2
PEPD
TBCD
MT-ND5
KEAP1
MYPN
MASP2
CFLAR
FOXM1
PCDH19
SORL1
PHYH
LGALS3
C1S
OSTM1
BOLA3
CLN3
CXCL1

PHOX2B
NCF4
PF4
GAN
PPA2
MYBPC1
CPQ
PIK3R3
PRLR
FOXG1
PER2
THRA
LZTR1
NPRL3
NCF1
MMUT
FAT4
IL11
ITPR1
NME1
KMT2D
RUNX3
KRT20
IGHM
PKM
TUBB1
CDKN2C
MAN1B1
ITM2B
EXT2
F11
NUBPL
ANKS6
CEP152
TDO2
KCNA1
PRKG1
NDE1
E2F1
SLC22A12
ZMPSTE24
SLC25A12
FGF14
TTBK2
XRCC2
RETREG1
ACO1
MDK
MFF
CRAT
CTNND1
TRPV1
UGT1A7
TPO
ASCL1
LBP
NQO2
KIF23

TPI1
DUOX2
CHRNA7
ATP1A3
ERCC3
TANGO2
TMEM165
MAP2
PLAGL1
GP1BA
VPS11
KDM1A
NANOG
KCNMA1
IL22
ITPR2
CLN5
ISL1
CDX2
HDAC4
LEF1
SAG
INPPL1
ARL13B
PLIN1
IL15
SERPINA7
TGM2
PCSK1
RNASET2
LTF
CEBPB
IDO1
THBS1
RYR2
GALK1
EEF1A2
NOG
MSX2
APRT
ASCC1
ENPP2
AKR1B10
UNC13A
KCNH2
ALDH1B1
NGFR
CYP2B6
FAM20C
UBA5
GPX3
TAC1
RAC2
SLC2A3
MX1
CCKBR
HPX
MIP

CDK5
IRF3
KISS1
POLR3A
HK2
CHGA
ITPR3
CHKA
ADAM10
SLX4
EFTUD2
LGI1
EPHA2
BACE1
ARX
NSMCE2
LIFR
TRIP13
APEX1
XRCC3
PRKD1
UNC13D
CEACAM1
LAMA2
EIF4EBP1
DGAT1
PEX11B
GALT
FBN2
DSP
CHKB
FLG
AFF2
BBS5
S100A8
APOL1
MSH3
SCO1
DCX
LHCGR
ANPEP
BRIP1
PCCB
CTTN
SLC22A18
FAT2
CES1
CDC6
UQCRB
PRDM10
DDR2
SEC24C
AP4M1
FXYD2
PNOC
GSTO1
QDPR
BMP1

SPTLC1
CNTN2
TBX20
H6PD
AQP1
SCN4A
PLIN2
SLC2A9
AUTS2
VDAC1
DNA2
GLS2
PNPLA6
MYH6
EWSR1
CDC25A
SC5D
GPD1
COASY
ADORA2A
TYRP1
AQP4
GSTM3
TXNRD2
ZBTB16
CTSG
BRD4
ABCC4
CS
RECK
PHKA1
ASPA
KLK3
BLM
AXL
BGN
SUMO1
BCR
FANCL
COX8A
LYZ
BBS10
PTK2B
TTC12
GCLC
RAD50
EIF4E
CXCR3
PEMT
LTBP3
SLC1A3
PGAM2
SLC4A2
ATP12A
CCNE1
DYRK1A
FLAD1
AIFM1

GABRB1
WASF2
EXT1
PTPA
MAPK8IP1
RHCE
FLI1
ANXA1
HPSE
CYP21A2
PDE5A
PRKCG
MAPK9
BMP7
STAT5A
CSNK1D
CPA1
USF1
SULT2B1
BBS7
EPHB2
SIRT3
RTN4R
COQ8B
FUT1
HPGD
SNCAIP
RASGRP1
GAD2
EIF4G1
WASF1
SSTR2
RPE65
MYF5
RBBP8
PXN
CENPJ
NLRC4
IBA57
NIN
SOX4
IRAK4
MCM2
UNC93B1
CSNK1A1
SCN9A
WDPCP
SLC9A1
UBE2O
SP7
HRH2
AAAS
STMN1
PALLD
ALG13
AGK
UFM1
UBR1

GSTA1
GZMB
BLVRA
KMT2B
PDSS2
GNRHR
HAVCR1
ANGPTL8
TGIF1
VARS1
SNAP25
SULT1A3
BBIP1
LMBRD1
ITGA2B
SCYL1
KCNA2
GLYCTK
CYP11A1
CXCL2
CIB1
KIF7
ASGR2
SYNE2
AURKB
PNKP
ATOH7
PROC
PLCG2
CD82
TSFM
CXCR2
CASP2
MTA1
PIGR
TUBG1
CTH
LECT2
USP9X
MPDZ
ASPH
CIDEC
PEX7
PAX5
CCKAR
PPBP
PRSS2
APOD
UGT1A6
MVP
GFPT1
PPARD
TBXAS1
CXCL6
SLC39A13
CD59
PSMD14
UCHL5

HPGDS
RIPK3
HSD17B13
GABPA
ST8SIA4
MPRIP
NOX4
MYOM2
XPR1
DERL2
CHST3
SPHK2
PIWIL2
PSMC5
ROS1
LIN28A
MBOAT7
VLDLR
HDAC11
PPP1R2C
S100A4
SAA2
DEPTOR
SKP2
SFRP5
IL33
NXN
ST6GAL1
SF3B3
ALOX15
CERS6
NCAN
FST
YME1L1
TNFRSF13C
REG3G
SLCO6A1
DOCK11
CLEC4E
ARNTL
ARRB2
MUC2
NFE2
GSTK1
IL37
GSTA4
IL19
HLA-DOA
HCAR2
FFAR4
IL6ST
IL16
PLEKHG5
FOXD3
DNAJC6
TNNI3K
ATP1A1
OTCH2NLC

ECM1
ZNF687
COPA
FMO2
INAVA
HOXD10
PDE11A
SP110
GIGYF2
ATG16L1
AGXT
POGLUT1
SEC61A1
ATP2C1
DZIP1L
GNB4
PPM1K
TRIM2
PDE8B
LEAP2
SAR1B
IRGM
#N/A
DHX16
COX7A2
SFRP4
ARPC1B
RINT1
IRF5
JPH1
PMP2
ZFAT
SLURP1
CRB2
LRSAM1
GLE1
DHTKD1
