# Supplementary material for: Network Pharmacology-Based Exploration on the Intervention of Qinghao Biejia Decoction on the Inflammation-Carcinoma Transformation Process of Chronic Liver Disease via MAPK and PI3k/AKT Pathway
Source: Biomed Res Int. 2022 Oct 14;2022:9202128. doi: 10.1155/2022/9202128 (PMC9586778; doi:10.1155/2022/9202128)
Supplement: Supplementary Materials — Supplementary data associated with this article can be found in the appendix. Supplementary file 1 shows the compounds and their associated targets for QBD and Supplementary files 2-8 show the associated targets for CLD. [file 9202128.f1.zip › HC target.pdf]

HC target（Results after deleting duplicate targets）
TNF
IL6
IFNG
F5
IL10
F2
HFE
TP53
FAS
PROC
CCR5
CD81
TLR3
LDLR
INS
STAT1
MYC
CLDN1
DDX58
GPT
LIPC
CD40LG
FLNC
TYMP
APOE
ALB
RAF1
HLA-DRB1
PIK3CA
SERPINC1
MET
IFNL3
TBK1
LMNA
CRP
FOS
FASLG
EGFR
MAPK8
IFNA2
MAPK1
HNF1A
KRT18
HNF4A
TYK2
STAT3
KRT8
CTNNB1
CTLA4
EGF
SCO2
KRAS
AFP
NPC1
NR1H4
TNFRSF1A
APOA1

IGF1
CASP8
GGT1
CYCS
CXCL8
LPL
HLA-B
HLA-C
CXCL10
SCARB1
JAK2
HLA-A
CCND1
POLG
AKT1
SERPINA1
PPARG
JUN
MT-CO1
HAMP
IL1B
SURF1
ERBB2
HRAS
IFNL4
PSAP
ICAM1
COX15
GRB2
EIF2AK2
FOXP3
TGFB1
FANCC
NFKB1
CHUK
CLEC4M
CST3
PTPRC
COX5A
DDX3X
POLG2
INSR
VEGFA
XPC
APOB
HMOX1
TERT
CFTR
COX10
SLC17A5
SRC
PTEN
CASP3
CYP2D6
SMAD4
BRAF
HGSNAT
SMPD1

BCL2
BRCA1
HADHA
HLA-DQB1
IFI27
IFNB1
TLR4
APOC3
XIAP
ALAD
UQCRC2
CDKN2A
IRF3
MT-CO3
JAK1
MAT1A
CP
IL4
ABCB11
CAV1
PIK3R1
CYP3A4
ABCB4
HGF
PRKCA
MPO
IGF1R
APOC2
COA8
MMP9
MX1
IGF2R
SLC2A1
AKT2
SCO1
APC
CPT2
TGFBR1
APP
NPC2
COX8A
MAVS
COA6
NPHP3
NOS2
IL2RA
IL2
G6PC
G6PD
CLDN6
CDK4
CCL2
MT-CO2
RSAD2
CLDN9
COX6B1
CD79A
RRM2B

PRKCD
CYP2E1
LRPPRC
HBB
KIT
IFNAR2
IFIH1
TICAM1
MBL2
PTPN11
TWNK
BAX
FGFR1
IL1RN
HJV
DGUOK
HADH
ABCA1
SLC25A13
CDKN3
COX4I1
PKHD1
CREBBP
PDGFRA
MAP2K1
NRAS
TACO1
SOCS3
FLT1
COA5
MPV17
NPM1
PKD1
CCL5
IRF7
CDKN1A
NOTCH1
EP300
ABL1
RB1
ACADM
HM13
UGT1A1
THBD
MAPK3
TLR2
CYP1A2
IL18BP
CYC1
IFNAR1
ENG
LEP
IFT140
CCR6
MT-CYB
FADD
CASP9
BCL2L1

HAVCR2
JAG1
COX14
MTTP
NFKBIA
FAH
FECH
CD4
ABCB7
IFIT1
IRF9
FGF2
ABCB1
CXCR4
ASS1
HLA-DQA1
EPO
MMP2
VIPR1
CPOX
CTSC
PTGS2
COX20
HADHB
SP110
RBCK1
PRNP
GCK
TRMU
PRKCB
SLC25A4
NFE2L2
MTHFR
ATM
TYR
CDH1
PLCG1
CXCL12
UROD
IKBKB
ISG20
F3
TF
CDK1
PNPLA3
SLC40A1
SERPINE1
SLCO1B3
IL12RB1
CYP1A1
ADIPOQ
NDUFS8
MAPK14
AXIN1
TFR2
CYP2C9
IL18
VEGFC

NOS3
HIF1A
LIPA
IL1A
PRTN3
PSMA7
BRCA2
ERCC6
ESR1
ABCC2
AOPEP
CSF3
PLAU
ABCC8
HNF1B
BTK
HLA-DPB1
TCF4
LAMTOR5
E2F1
ATP8B1
TSC2
PET100
SLCO1B1
GBA
CREB1
RNF31
HMBS
GYS2
TMEM67
EIF2AK3
ASGR2
IKBKG
MPL
BIRC5
AIRE
PPARA
MDM2
OFD1
ATP7B
SQSTM1
CAT
PTCH1
UQCRQ
MRPL44
TRPV4
RSF1
CETP
KDR
MT-ATP6
KRT19
SOS1
PCSK9
MTOR
ALAS2
DDIT3
MMP1
HSD3B7

ABCG5
CCL3
PDGFRB
PKD2
IL2RB
CXCR3
ACADVL
PTPN22
BSCL2
RAC1
NTRK1
GBE1
TNFSF10
HAVCR1
RPGRIP1L
RET
SPP1
CD44
ERCC1
ETFDH
TLR9
WDR19
CD19
STK11
ASGR1
MIF
MEFV
APOH
ALDOB
AKR1D1
IL6R
SEPSECS
OTC
CFLAR
CDK2
NLRP3
VWF
ACAD9
SLC10A1
OTULIN
FASTKD2
SFTPC
RELA
FBP1
SST
HSP90AA1
HSPG2
SLC25A20
SMAD3
MTUS1
SKP2
TTR
IRS1
PTK2
TJP2
TIMP1
MCL1
PPOX

CCR7
MT-ND1
PIK3CG
ZMPSTE24
CXCR1
THPO
BCR
TSC1
CD34
FGA
PROCR
RYR1
IL17A
CD40
IFITM3
LRP5
COL11A1
HSPA4
SERPINA3
STEAP3
TGFA
MYB
DPM3
IGF2
BCS1L
AGPAT2
SH2D1A
IL13
PTK2B
BIRC3
CDKN1B
CC2D2A
CCN2
VIM
PRKCZ
KCNJ11
CAVIN1
CD247
IDH2
CCNA2
NGLY1
LOX
DPM1
PRKCSH
ANXA5
PLAT
NOD2
PET117
UROS
GSK3B
CD36
GATA1
TM7SF2
QDPR
POMC
GAPDH
GNG13
PMM2

ATG5
MRC1
TFAM
CIITA
ABCC1
ACE
BMP6
GNMT
PCNA
IRF1
CD55
CASR
FGFR2
UQCRB
SOD2
SP1
MUC1
ALPP
UQCRFS1
MARS1
PAPOLG
BAK1
IL5
IL3
TFRC
FANCA
MT-ND2
HLA-DPA1
F8
CBS
SKIV2L
CYP2C19
WRAP53
BAD
SLC30A10
PDGFRL
SHC1
IFT122
CFH
ESR2
EDN1
AR
RPS6KB1
SIRT1
VCAM1
SPINK1
PC
CEBPB
UCHL1
RAG2
MEN1
NF1
NPPC
DAG1
ALG8
DNMT3B
KLF6
TGFB2

NOTCH2
CYP19A1
SAR1B
CD96
ETS1
ABCG2
F9
FLT4
IL1R1
ITGB1
CSF2
APOBEC3G
TNFRSF1B
HMGB1
GANAB
ATP7A
CD8A
FANCI
PNKD
IFT172
FANCD2
ELANE
APOA5
GSTP1
NFKB2
CALCA
BGLAP
KITLG
CYP2B6
RTEL1
CD209
CCR3
CD27
XBP1
IGFBP3
TRAF3
SLC22A5
HSPA1A
VDR
ETFA
DMD
MAPK10
HP
SREBF1
COL8A2
RAD51
CCL20
ERCC3
HSPA5
DEPDC5
TIMP2
CCL4
MPI
RAG1
F10
MVK
GOLM1
GZMB

CHGA
FOXA2
UQCC2
PRF1
MME
CD80
CPD
JAK3
IGHM
ALG2
CXCL9
WRN
MYD88
HSPB1
PTPN3
GSTM1
CEACAM5
FOXM1
DKC1
RASSF1
DDB1
RARB
ADAR
CD274
CASP1
PTMA
TTN
YARS2
ENO1
EIF2S1
NCAPH2
IL21R
COX4I2
FASN
NQO1
PDX1
PRKCG
ACADL
CASP7
KLRK1
BMP2
TNFRSF10B
CDK6
KRT7
YY1AP1
NR5A2
NAGLU
HNRNPK
PLA2G6
RXRA
MMACHC
GLIS3
TRAPPC11
EGR1
TNFRSF10A
CHKA
CYP7A1
PRKCE

NDUFS1
ALDH2
DRD4
MT-ND4
WDR35
CCNB1
FYN
NR3C1
CD69
SHBG
E2F2
GPS2
FN1
CSF1
ACTB
ANO5
SPCS1
BLK
SFTPB
IKZF1
SLC4A1
MYBPC3
DBH
EVC2
AKT3
ABCC3
CCR4
FABP1
PDCD1
COQ2
LEF1
IFT43
HDAC1
ACADS
LTF
SREBF2
UBC
ERCC4
CYP2A6
CXCR2
SEC63
TSPO
ARG1
RETN
NBN
LMNB2
RPS27A
TP73
RHOA
IFITM1
ATF4
ELK1
PLG
STAT4
EPHA2
MKI67
PPIA
CHIT1

PIK3CB
CLPX
GFAP
TTC19
NLRC4
SNCA
SMAD2
CDC25A
MLXIPL
SOCS1
TRAF6
CCR1
PTF1A
PI4KA
GLUL
PUS1
PRKD1
LIG4
P4HB
GNPTG
SELE
SERAC1
S100B
PIK3C2A
NME1
ERCC2
NDUFAF5
IL12A
SCARB2
POMT1
BID
GDF5
DIABLO
CR2
STAT5A
PSMB8
TNFAIP3
GJB1
AGTR1
TMPRSS6
POMGNT2
IL12B
TAT
SSB
PPARGC1A
EZR
FGFR3
MBP
CXCL1
CR1
TGFB3
PLCB1
ERCC8
RIPK1
IL15
TNFRSF13B
MAP2K2
AURKB

ATRX
DGAT1
ANGPT2
PDGFB
LEPR
SPRTN
FGB
ERBB3
GH1
DLD
HMGCR
CBL
CCL11
TLR5
FTL
COG2
CD28
LCK
NR1I2
CEP290
CD86
SGSH
RRM2
CALR
PNLIP
GAST
CXCR5
BECN1
PEX6
CCL18
APOC1
PYGM
FBN1
NLRP12
GYPC
FANCF
FKTN
PLK1
TM6SF2
B2M
GUSB
NEK8
MMP14
TRIM32
NOTCH4
HEPACAM
IRS2
IL10RB
REL
PCK1
FKRP
HCCS
FARSB
SELP
HSPD1
AP1S3
PATL1
CDKN2B

SLC10A2
TGFBR2
FGFR4
GCG
CEBPA
LCAT
FGF23
FOXRED1
GYPA
CAPN3
CCL17
KLRC1
CTSD
MMP7
CYP3A5
PLAUR
SSTR2
CTTN
IGFBP1
POMGNT1
SMARCA4
NTRK2
TLR7
ADA
PRKN
ITGAL
IL7R
HLA-G
SPARC
ABCA3
DHFR
FIP1L1
TOP1
DNAJB11
DKK1
CEL
TK2
PCLAF
XDH
ACVRL1
PPIG
LYN
IL7
PXN
FGL2
HPX
MUC5AC
MMP3
ELN
KDM6A
AURKA
ITGAM
ETFB
TNC
GPC3
CPQ
PODXL
OCLN

MAP3K5
TBC1D20
AREG
KRIT1
GRIN2B
RBP4
ACKR1
KMT2D
ZIC3
GFER
SPTAN1
SHH
ALG6
PRL
PTPN1
RFX5
PML
GPC1
CLU
NHP2
CCDC85B
DLL4
DPYD
AIP
POLR2A
NCAM1
CHEK2
AKR1A1
APPL1
MAP2K7
SHARPIN
SLC6A4
YAP1
RAB7A
RHOC
PRKCQ
ENPP2
PIK3CD
TNFSF13B
TPMT
SELL
PIEZO1
FANCG
IGHE
MSH2
VIP
PALB2
TIMP3
NPHP1
DNAH8
PIK3R2
CTH
PNPLA2
TCF7L2
ANGPT1
ITK
NR1H3
UGCG

F7
MS4A1
TRIM25
NARS2
GSR
LYRM7
CCK
CASP10
SNAI1
SLC2A2
BIRC2
MICA
GHR
SMAD7
AGK
TAP2
CTSA
CCL22
STAT2
EZH2
POMT2
MAPK7
ACAN
NR1I3
CNTNAP2
UQCC3
HK2
C1S
HDAC9
TXNRD1
BRD4
ALG11
NOP10
C1QBP
LTA
EPHX1
CCR8
PLA2G12B
CDKN1C
CXCL13
SDHB
DOLK
TNFSF11
BDNF
BCL2L11
AMACR
KCNN4
LMF1
VCP
RACK1
TNFRSF11B
PLCB4
SLC11A2
CCR2
FCGR2B
NPC1L1
VAV1
ITGB3

MGMT
DLAT
FURIN
PCK2
PSEN1
TRIM22
ECE1
PLCG2
PECAM1
TH
SRD5A3
STAT5B
STN1
GRP
ZEB1
DNAJC19
SLPI
RPL18A
PON1
AGT
PSMB9
CX3CR1
FBXL2
CPA1
SMURF2
NANOG
UBB
HSP90B1
MT-ND6
ATP11A
CD14
HSPA8
VHL
PIK3R3
FRZB
PROS1
SLC23A2
PMS2
PF4
CEP55
ALDH6A1
TWIST1
KNG1
MKS1
TG
SP140
WNT5A
RAP1A
BSG
MARCKS
WT1
MAPK9
RUNX2
PAH
CTSG
SBDS
IL4R
CSK

POU2AF1
ANXA2
CYP2C8
PRSS1
ACP1
CD3D
TNFRSF13C
CPE
LAMP1
IQCB1
NPHP4
CRH
OGG1
EPCAM
MT-ND5
LMBRD1
PRKAG2
TNFRSF8
NGF
STAT6
TMEM216
PROM1
KIF1C
APAF1
CDH2
NDUFAF1
ATF6
CCL21
ACY3
SLC6A3
ADAMTS13
SULT1A3
XRCC4
ENPP4
BCL6
CYBB
MYH7
PLCE1
LCN2
LGALS3
SMARCA2
CCL26
CD22
MAD2L1
HELLS
SERPINB3
HUWE1
TTC7A
GSTO1
CD33
TFAP2A
TARS2
ICOS
ODC1
CEACAM3
NUAK2
SERPINF2
CPT1A

WNT1
AICDA
SLC23A1
FLVCR1
STMN1
STING1
POU5F1
GAA
SFTPD
MAOA
NEUROD1
FCGR2A
IL1RAPL2
CCNE1
CTC1
HTR2A
ADA2
BTC
DNAJC21
C3
SSBP1
ACOX1
RECK
PRMT7
EIF2B3
TKT
RPS6KA1
ASAH1
CXCL11
CD59
CXCL5
GAMT
UGT1A7
SCD
CFP
GDNF
SYP
TFEB
COG4
CLEC12A
COPS5
OPRM1
LIN28B
F2R
NOS1
ENTPD7
COX7B
ALAS1
ICOSLG
ZAP70
PHF20
ITPA
DDX60L
CCND2
GHRL
GSTA1
PLA2G4A
ASXL1

ACTG1
IL17F
NCF1
LOXL2
MERTK
RTN4
GPR158
CAP1
SORBS1
RBM45
CLDN7
BMPER
AGRP
FBL
ARHGAP26
REXO1L1P
KIAA1549L
GEM
CBLIF
AGO2
IFNL2
IFNL1
MIR7-3HG
ERVW-1
HSF4
KIR2DL3
KIR3DL1
ERPINA13P
LNPEP
NCAM2
OAS1
OXTR
PAEP
IL22
SERPINB6
SDK2
PKIB
PLAAT4
RIT2
BRD2
ERVK-6
ROBO3
SOAT1
UBE2B
GLT8D2
SPATA16
CAV3
ARTN
HACD4
GGTLC1
RNF7
IVNS1ABP
ISG15
NR1D2
SLCO6A1
GSTK1
TLR8
ISYNA1

PSME3
TRIM69
GABPA
IL21
GGTLC3
GGT2
EIF3A
CD38
CD68
KIR2DS2
KHDRBS1
CHP1
IFNLR1
EIF2S3
ITIH4
SMIM1
LGALS3BP
KRT20
SLC12A9
EIF2S2
PPIP5K1
USP18
CLTC
DPP4
EPHB2
FCGR3A
FCGR3B
SEC14L2
NUP62
HPGDS
GTF2H1
TBX21
KIR2DL2
LGALS9
MIP
NTSR1
DCTN4
GOLPH3
CNBP
CD163
PLIN3
TMED2
TPPP
CHI3L1
PLIN2
EMB
NRSN1
AHR
SLC29A1
FCN2
AAK1
FOXO1
IL27
GAK
LAMP3
PPP2R3B
ACACA
HLA-DRB3

KIR3DS1
LAG3
LSAMP
CD99
MT1B
GDE1
PI4KB
IL17D
WDR11
RNASEL
SDC1
SMOC1
NR0B2
BOC
CD9
BMS1
ERVK-9
CDSN
ATG7
ERVK-19
IFI44
AHSA1
MASP2
SUB1
BTG3
AP2M1
CYP2R1
CRK
PDIK1L
CUX1
CYP27B1
DDOST
ATN1
EIF4A1
EIF4A2
EPHA3
ABCD1
FOXO3
MCF2L
CABIN1
IFI6
RNF19A
POLDIP2
IL37
ABO
GP2
UTS2R
ANXA6
HLA-E
APOA2
IGHG3
APRT
IDO1
INSRR
IRF2
IRF5
KIR2DS1
KIR3DL2

MFAP1
NF2
OAS2
OSBP
NOX4
IL23A
PPIB
BACH1
PELI1
PTBP1
PTBP2
RAB5A
TRIM27
S100A9
SMYD3
TAP1
TAPBP
TLL1
TRIO
UBE3A
USF1
VTN
XRCC1
MBOAT7
AIMP2
MAP1LC3B
SLC14A2
FAM107B
UBASH3B
GBF1
PER2
SLC28A2
DCLK1
VAPA
PRDM6
GRAP2
NCR1
APOBEC3B
SART3
ZGLP1
ABCB6
ERVK-11
MYMX
NDRG1
NXF1
CREB3
SEMA4D
MRPL28
IGF2BP1
CES1
CCL27
HPSE
HCP5
WDHD1
DCTN3
PHB2
CKB
CISH

CNR1
ATF2
CREM
CRHR2
RMDN2
CYP2D7
DCC
HFM1
DCX
DECR1
DHCR7
METTL4
SARDH
DYNC1H1
SLC26A3
ARID2
EIF4EBP1
EIF4G1
TIGIT
ERN1
EXT1
FAP
FBN2
FGL1
ATG14
FLNA
FLNB
PMPCA
PPP1R13B
FOSB
FPR2
FTH1
NCR3
ASPM
ATRNL1
GC
B3GAT1
PDLIM3
GLI3
GLP1R
GPX1
GPX2
NXT1
RACGAP1
GSTT1
HINT1
HLA-DOB
HLA-DRB4
APCS
HPS1
IFNA6
IFNGR2
IL10RA
ILF3
IRAK1
ITGA2
ITGA2B
ITGAX

JUNB
JUND
CD82
ARF4
KLRB1
KLRD1
LBP
LGALS1
LIPE
ARNTL
TACSTD2
MAGEA3
MCM2
MCM7
MICB
MLH1
MNAT1
NCL
NHS
PCBD1
RMDN1
HERC5
PGF
XRN1
DDX56
PPP1R12C
MARCHF1
ATG16L1
PPP2CA
RMDN3
NAT10
KIR2DL5B
SYBU
AXL
PKN2
MYDGF
AKR1B10
KIDINS220
SEMA6A
BCHE
RNH1
CLIP1
RTN1
SAA1
SRL
CCL8
CCL14
SELENOP
SET
SRSF4
SLC25A1
FUNDC2
SPINT1
ST14
TJP1
TPT1
POTEF
TNFSF4

SUMO1
CLIP2
DHX58
TNFAIP8L2
EHMT1
CAMKMT
CALM1
ASRGL1
LPAL2
CALM2
CALM3
ARHGAP24
EIF2A
ULK1
POLDIP3
NTPCR
SARNP
NT5C1A
AP3B1
TSLP
PIAS1
USO1
OASL
MBTPS1
CDK5R1
ARHGEF7
HSPB3
CCNG1
CBFA2T2
MTG1
GDF15
EIF5B
DCAF1
CDC42
SYCE1L
CD24
ERVK-10
ERVK-21
ERVK-18
ERVK-25
ERVK-24
ENAM
OPTN
BCAP31
G3BP1
TNK2
DDX39A
KLRG1
RABEPK
IGSF6
CDK2AP2
SIGMAR1
CDKN2C
RTN3
LANCL1
CDKN2D
CRISP3
TLR6

TUBA1B
BTN3A3
PIAS3
NDC80
TOMM40
CIB1
ZNRD2
UPK3B
PDLIM5
ERLIN1
PDPN
RAD51AP1
SPINT2
CELF1
CXCR6
CD226
DCTN6
EBP
PTGES3
KDM5B
STARD3
TOMM34
RAB40B
EBNA1BP2
TMED10
RAB32
IMMT
SLC27A4
SLC27A2
IL24
GTF2A1L
STON1
PIM2
RPP14
BTN3A2
SLC7A9
NUDT3
RNF139
CAVIN3
PACSIN2
SNF8
SLCO2B1
CDCA5
CD300A
GABARAP
CARD16
PGLYRP2
OSBPL1A
CIRBP
CLCN3
ADH1A
ADH7
CNR2
TAF8
COMT
CD200R1
NCOA7
CLDN4

CPS1
CRABP1
CRABP2
CRY1
CRY2
CRYGD
CRYZ
VTI1A
CSNK1A1
CSNK1D
CSNK1G2
CSNK1G3
VCAN
NCAN
CD300LF
APCDD1
IL23R
CTSB
PLB1
BTLA
CTSS
CYBA
CYP2A7
CYP2C18
CYP17A1
CYP24A1
CYP27A1
DAPK1
DAPK3
DDX6
DHX9
LIX1
DFFA
SLC30A8
CLEC4C
AFM
DIAPH2
DNASE1
AGL
DUSP1
DUSP6
EDNRA
AHCY
EDNRB
EIF2D
EFNA4
EIF4E
AIF1
ELAVL1
ELF1
CRTC2
STX2
FABP2
ACSL3
FAT1
MED19
FCAR
FCGR1A

RBM24
XRN2
FKBP4
FKBP5
CARD8
MMRN1
RAB18
FOXC1
TPX2
CNOT1
KDM1A
SMG1
ERC1
KIF1B
AKR1B1
TBC1D9
ARHGEF9
LARP1
GPR161
QPRT
FPR1
CD2AP
PPP1R15A
OSBP2
FKBP8
IFIT5
FUT1
NEIL2
FUT8
XRCC6
CASC2
SHC2
GAP43
KRT23
LDLRAP1
GAS6
PHGDH
GALNT8
GBP1
HSPB8
GCHFR
GCKR
AGO1
IL36RN
GFRA1
HBP1
AMPD1
ATP2C1
SND1
NAAA
VPS4A
TNFRSF21
TNRC6A
UBE2S
SGSM3
GOT1
GOT2
OR10A4

IGHV4-59
IGHV1-69
IGHV1-3
IGKV3-20
LAMTOR2
MRPS18B
PYCARD
BRD7
PDIA3
GSTM2
GUCY1B1
HILPDA
IL19
GZMH
H1-5
ANXA1
HABP2
HARS1
HTT
NRG1
HLA-DMA
HLA-DOA
HLA-DQA2
HLF
HLA-F
MR1
HMGCS1
HMGCS2
HMOX2
SLC29A2
HNRNPC
HNRNPD
HNRNPL
HPCAL1
AGFG1
APEX1
HSPA1B
HSPB2
HTR6
IFNE
RAB7B
ICAM3
ARMH1
ID2
IFNA4
HSD17B13
IRGM
IFNGR1
APOC4
IGFALS
IGFBP4
IGFBP7
TICAM2
IL6ST
IL9
IL11
IL15RA
IL16

TNFRSF9
ILF2
IMPDH2
ING2
INPPL1
IRAK2
ABCC6
ITGAE
EIF6
ITPR3
ARF1
ACAT1
KIR2DS3
KIR2DS4
ARF5
KLRC2
KPNA1
TNPO1
KRT14
TBPL2
ACAT2
LAIR1
RPSA
LGALS4
LIF
SMIM10L2A
MYO18A
LMO1
LPA
CYP4F3
LTBP2
LUM
LY9
LY75
MXD1
SMAD1
SMAD6
MAGEA4
STS
MATK
MCM3
MCM6
MDM4
MFGE8
MAP3K11
MPG
PLIN5
MSH3
ERVK-7
MT2A
MYCN
GADD45B
MYLK
NAP1L1
RERE
NEK2
ATP12A
NFKBIB

NFKBIE
NFKBIL1
NPPB
NRDC
ACR
OAS3
ATP4A
SIX6
ALDH7A1
P2RX4
P2RX7
IL20
PAK2
PAK3
PAX5
TRAT1
TMED5
TMED7
HDDC2
CHCHD2
JPT1
GP6
GLTP
MARCHF2
PCYT1A
PLA1A
CRLF3
DDX41
UBE2J1
DCDC2
NCKIPSD
CINP
AZIN1
SF3B6
PPIL1
ENPP1
SLC9A3R1
CD244
ERAP1
PER1
PGGT1B
PHB
PIK3C2B
PIK3C3
ACP2
PITX1
PKM
PLA2G1B
ZFYVE1
ATP6V1G2
PLEK
PLIN1
PLXNB1
POLE3
TREM1
POLRMT
SMOX
POU2F1

PPARD
CRLS1
FEV
AHI1
DYM
SIDT1
NSUN2
PPM1A
RNF125
ODR4
UCKL1
TMEM132A
MED9
AVP
ARL8B
ADI1
FBXW7
DDX19A
LAPTM4B
IMPACT
PREP
PRIM2
PRKAA1
VAC14
ATF7IP
HHAT
TDP1
USE1
HDAC8
APOM
DNAJC3
CTNNBL1
PRSS3
MASP1
RELN
KLK10
BCCIP
ZC3HAV1
UGGT1
ARNTL2
CMC2
CHPT1
PSMD3
PSMD7
PSMD9
PSMD10
SALL4
KIR2DL5A
PTH
RPTOR
KLHL1
TNRC6C
PTPN2
TRIB3
PTPN6
PTPN7
ZNF410
ACTA1

PTX3
SCAF1
RAB1A
RAB6A
RAD21
RAD23B
RARA
RASGRF2
RBL1
REG1A
BCL2A1
REN
RENBP
RHEB
RNASE1
RNASE3
RNASE4
HPSE2
BCL9
ROBO1
RORA
RORC
ROS1
OPN1SW
RPL17
RPS4X
RPS6
ERVK-8
RPS6KA3
RRAS
S100A1
SAG
SERPINB4
CX3CL1
SDC2
SDC4
SDHC
SEL1L
SLC28A3
SMOC2
SFRP1
SFRP2
SRSF5
COP1
HHIP
BLVRA
SMIM10L2B
GORASP1
YTHDC2
PMEL
PINK1
ACD
BMP3
WNK1
SLC3A2
UBE2Z
PRB2
FAM72B

SLC6A12
SLC12A3
SLCO1A2
SLC22A1
BMPR2
SIGLEC1
SNRPD1
SOX2
SPRR2A
SRD5A2
SRY
TRIM21
ST13
STAU1
STX4
SULT2A1
SURF4
BST2
SYK
SYT1
ADAM17
TBP
BTN1A1
TERF2
TFF3
TGM2
THBS1
THOP1
TIMP4
SERPING1
TMSB4X
TNFAIP6
TOP2A
TOP3A
TPO
TPR
TRAF2
HSP90B2P
TRPC5
CAPN5
TULP1
TXN
FAM72A
UBA7
SLC35A2
VPS51
VLDLR
VRK1
WAS
WEE1
XRCC3
XRCC5
YWHAZ
CA2
ZNF185
DDR1
DNALI1
IL1R2

MAPKAPK3
CAD
GGCT
FTO
MAPKAP1
DDX39B
NELFE
EFL1
NEIL1
NLRX1
SUV39H2
ARHGEF5
LPCAT1
ZC3H12A
EDEM3
ULBP1
PDCD1LG2
MPIG6B
APOL3
SETD7
TRIM56
RAB1B
USP11
BAP1
FCRL4
RILP
BCL2L12
SESN2
MIXL1
KAT8
ASCC2
STX7
CAVIN2
DUSP11
DHX16
MAP1LC3A
ZGPAT
DGAT2
CREB3L3
SPSB2
OGT
ALG10
ITPRIP
DNAJC5B
MAPKAPK5
PLA2G4C
TP63
AOC3
RUNX3
NUMB
CBLB
PEA15
TNFRSF25
RIOK3
IL1RL2
CES2
NRP2
HDAC3

PER3
IER3
FUBP1
DDX18
TIMELESS
RAB29
WNT3A
CH25H
UNC5A
PIAS2
FCGR2C
CD1C
CD1D
HGS
ARHGEF2
CD5
MTDH
VAPB
IL32
SPECC1
AIMP1
CD8B
PDLIM7
GPR55
NEURL3
IGSF8
ZFYVE9
TGFBRAP1
NTN1
GGPS1
TBPL1
GOSR1
CLOCK
RBM39
PITPNM1
SEC24C
CD58
CD63
SDC3
EDEM1
TOX
RAPGEF5
CDA
SPATA2
TRIM14
CDK11B
FARP2
DDX46
MFN2
USP15
MVP
HNRNPDL
PLCH2
PLEKHG5
DNAJC11
C1QC
RHCE
NUDC

YARS1
RRAGC
UQCRH
COA7
FAM72C
POLR3C
OTCH2NLC
THEM4
CRCT1
MEX3A
CD1A
XCL2
XCL1
PIGC
MTARC2
MTARC1
LIN9
FAM110C
DNAJC27
DNAJC5G
DPY30
PRKD3
COX7A2L
COX5B
ATP5MC2
DNAJC10
PLCL1
CRYGC
CTDSP1
SCG2
SSUH2
CIDEC
IL17RC
NR2C2
PLCL2
CMC1
PLCD1
SEC22C
XCR1
ALS2CL
SMARCC1
UQCRC1
GMPPB
MST1R
PLCXD2
COX17
ZXDC
GP9
DNAJC13
PCOLCE2
PLCH1
SMC4
PRKCI
ABCC5
CTBP1
COX7B2
CWH43
COX18

CXCL6
CXCL3
CXCL2
LIN54
UNC5C
GSTCD
TRPC3
NR3C2
PDGFC
ANP32C
ATPSCKMT
NPR3
PLCXD3
CCL28
COX7C
MEF2C
PPIC
CXCL14
FAM53C
PCDHAC1
PCDHAC2
PCDHGC3
PCDHGC4
PCDHGC5
PACSIN1
SNRPC
UNC5CL
TBCC
ABCC10
POLR1C
EFHC1
COX7A2
CCNC
CITED2
SCAF8
FAM20C
COX19
NDUFA4
CRPPA
COA1
VWC2
DNAJC30
POM121C
CCL24
PCOLCE
DNAJC2
FAM3C
CLEC5A
ZBED6CL
POMK
CEBPD
ELOC
COX6C
GSDMC
PLGRKT
CCL19
IMPDH1
IFNA5

SSTR4
IFNW1
