# Supplementary material for: Network Pharmacology-Based Exploration on the Intervention of Qinghao Biejia Decoction on the Inflammation-Carcinoma Transformation Process of Chronic Liver Disease via MAPK and PI3k/AKT Pathway
Source: Biomed Res Int. 2022 Oct 14;2022:9202128. doi: 10.1155/2022/9202128 (PMC9586778; doi:10.1155/2022/9202128)
Supplement: Supplementary Materials — Supplementary data associated with this article can be found in the appendix. Supplementary file 1 shows the compounds and their associated targets for QBD and Supplementary files 2-8 show the associated targets for CLD. [file 9202128.f1.zip › LC target.pdf]

LC target（Results after deleting duplicate targets）

HFE
TNF
NR1H4
IL6
ABCB11
SERPINA1
ABCB4
INS
IL10
TGFB1
ALB
IL1B
HAMP
GPT
PPARG
ABCC2
ATP8B1
TP53
ATP7B
KRT18
FAS
PNPLA3
CP
FAH
F2
JAG1
CFTR
HLA-DRB1
GGT1
AKT1
TFR2
TF
CD40LG
HNF4A
SLC25A13
HJV
SLC17A5
FARSB
AFP
IFNG
MET
CTNNB1
RETN
APC
POLG
CTLA4
SLC40A1
DGUOK
RBP4
TERT
TLR2
TYMP
G6PC
KRT8
APOB
PKHD1
APOE

PIK3CA
PKD1
HSD3B7
OTC
IRS1
IGF2R
GPC3
SEC63
INSR
TM7SF2
MTTP
FECH
ASS1
CYP2E1
MARS1
AGL
CAV1
SMAD4
IL12RB1
NOTCH1
PHKA2
TTR
FOXP3
HNF1A
ENG
LIPA
APOA1
MPV17
EGFR
ICAM1
KRAS
F5
PHKG2
TJP2
ALDOB
MYC
GBE1
PRKCSH
BMP6
AXIN1
NFKB1
AKR1D1
FOS
FASLG
HADHA
IGF2
CDH1
TCF4
UTP4
CYP3A4
CASP8
TLR4
UROD
SLC11A2
MAT1A
BCS1L
HGF
IRS2

MBL2
JAK2
SLC30A10
CXCL8
IRF5
EGF
MTOR
SMPD1
ALAS2
HLA-DQB1
ADIPOQ
CCND1
HRAS
HBB
DLAT
GBA
GYS2
CYP2A6
PYGL
TALDO1
NFE2L2
IL12A
CPT2
CDKN2A
RELA
GNMT
CD81
LEP
PKLR
TNPO3
NOS2
NPHP3
TNFSF15
NPM1
TMEM67
KIT
PTEN
WRAP53
XIAP
SLC2A4
IFNL3
PKD2
SPIB
THPO
NPC1
NDUFS1
EPCAM
POU2AF1
IL7R
PDGFRA
RRM2B
ALG8
CYP7A1
LRP5
CDKN3
RPGRIP1L
ESR1
HLA-DQA1

HNF1B
TGFBR2
PDGFRL
LPL
NBAS
NR1H3
GANAB
ABCG8
CYP1A2
CLDN1
PPARA
SEPSECS
PDGFRB
TSC2
KRT19
UGT1A1
ERBB2
CYP7B1
TIMP1
CRP
TWNK
MMEL1
STK11
TSC1
SLC2A1
PTPN11
FTH1
CD36
PTPRC
CC2D2A
FLT1
POLG2
BMP2
ASL
AGPAT2
AIRE
ALDH2
HMOX1
MST1
MT-CYB
KRT7
SERPINC1
IGF1
NRAS
SRC
AKT2
SLC10A1
SLCO1B3
MAPK8
SREBF1
DLD
SERPINA6
GPR35
ACVRL1
LEPR
STAT3
CALR
DCDC2

SLC39A8
CDKN1C
GAA
PTCH1
FGFR1
NOS3
FABP1
ABCA1
DNAJB11
SERPINA3
BRAF
BSCL2
IL2
CASP3
ABCC3
MSH2
NOTCH2
VEGFA
BRCA1
TFAM
STAT1
AR
SERPINE1
LMNA
SLCO1B1
NPC2
GLRX5
ACADM
ACTB
TM6SF2
MPI
MTUS1
GNG13
SST
ALAD
NR1I2
IDH2
SIRT1
HP
SKIV2L
COG4
FASN
TCF7L2
WDR35
ACADVL
TLR5
CAT
ALG2
EIF2AK3
CCDC115
TTC37
ABCB7
GDF2
ATP6AP1
FGFR4
CCR5
NHP2
OFD1

PMM2
ALPP
GAPDH
IKZF3
SMARCB1
ATP7A
ALG9
CCL2
NOD2
SLC37A4
NHLRC1
TRMU
UROS
MAP2K1
FTL
F3
TFRC
TINF2
FXN
KLF6
ABCB1
IDUA
TLR3
DDIT3
MMP9
GSTM1
IFNA2
IL4
PEX1
CAVIN1
AHSG
SURF1
IL17A
ALMS1
STEAP3
SOCS3
PTPN22
ETFDH
ABHD5
AMACR
MLXIPL
IL1A
XBP1
LIN28B
IFT43
ARG1
EPO
HMBS
UCP2
SPRTN
PPARGC1A
EDN1
IGF2BP2
CCN2
G6PD
NGLY1
SOD2
CYP1A1

MMP2
AKR1A1
NPHP1
DKC1
NR1I3
MPO
NOP10
ABCG5
HLA-B
CPOX
TREX1
INVS
SLC4A2
JUN
NPHP4
PGM1
TTC21B
RBPJ
MAPK1
RBCK1
CCR6
IL1RN
GATA1
SLC7A7
CYCS
FGF21
MOGS
CD4
MMP1
ERCC6
EPM2A
MSR1
ADIPOR2
CD19
DARS2
DLL4
ADIPOR1
MT-ND4
CYP2C9
ADH7
RTEL1
CEP290
STXBP2
TNFRSF1A
GALT
VDR
CD80
NR0B2
SETX
FGF19
MTHFR
PIEZO1
DNAH8
COG6
MCL1
ATF4
ADA
TLR9

SCARB2
AP1S1
MYO5B
ADH1C
GOLM1
SAR1B
B9D1
IFT140
HADHB
DPP4
HMGCR
ABCC4
BGLAP
FBN1
CD79A
IL18
GFER
TMPRSS6
CDKN1A
AHCY
TNFRSF13B
PRTN3
INPP5E
GLUL
IGFBP3
SLC10A2
IFT80
SLPI
GSTP1
SLC25A15
MKS1
HLA-A
F7
AHI1
REN
ATP11A
TMEM199
ADAMTS13
ENO1
SPP1
HIF1A
CHIT1
TAT
SCT
PTGS2
CD34
LCAT
KIAA0586
UNC13D
CDKN1B
IFIH1
UQCRC2
BLVRA
COG7
FBP1
PDGFB
ACOX1
PEX5

GLS2
HPD
PEX13
IREB2
RAG2
MAP3K5
PPOX
PEX2
CTC1
TCTN2
GPC1
CD44
BAX
RXRA
CXCL12
NPPA
ATM
CEBPA
LDLR
PARS2
ELANE
NDUFS8
KDR
RAF1
TMEM216
FABP4
BHMT
BBS2
GLS
MT-ATP6
TNFSF10
PAPOLG
B9D2
IFNB1
NFKBIA
IFNAR2
CXCL10
ACE
SCO1
CPT1A
PDHX
LCN2
TGFBR1
NARS2
ACACA
UQCRB
PEX10
SREBF2
IKBKB
CSPP1
HSD17B4
GALE
PEX6
JAK1
ITGB1
IL2RA
SCD
CEACAM5

FGF2
CFC1
GSK3B
SCO2
CDH17
ITCH
PHKB
CETP
PSMB8
SOCS1
CXCR4
ELN
PEX19
PRKAA1
IL5
ABCD3
MPL
IL6R
BCL2
PEX16
BIRC5
YAP1
PIK3R1
IL2RB
DMD
PEX12
CXCR3
CCL5
MMP7
PROM1
CYP2D6
RNF31
TGFA
TGFB2
SMAD3
PDCD1
MDM2
SP100
CDKN2B
MYD88
MAPK14
MUC5B
FCGR2A
SFTPC
ABCC1
FOXM1
MKI67
TIMP2
PEX26
BCL2L1
IL18BP
NUP210
IGF1R
BBS4
BBS1
SERPINF2
CFH
PRKAB1

UGT1A7
RSF1
SLC51A
ZIC3
SQSTM1
HSPA4
CALCA
ADAR
UQCRQ
GJB1
COG2
NAT2
B2M
TET2
CD27
TTC19
SH2D1A
GCKR
PEX14
EIF2S1
ASAH1
OGDH
FGA
FGB
GZMB
ADM
PEX3
CXCL9
PON1
ARL13B
CA2
DBT
SEC23B
LBP
PLAU
TMEM231
DLC1
PIGA
RB1
LECT2
APOC3
PLAUR
GRB2
IQCB1
HSPA5
MAPK3
CDK4
GALK1
RARB
HLA-DPB1
GPD1
CD274
RASSF1
STAT4
RHCE
EPHX1
NQO1
CYC1

GGT2
GSTZ1
GSTA1
MME
HSPD1
EZH2
CDH2
ACO1
UBE2O
CCL20
ANGPT2
LTA
MGMT
GATA2
VIM
SCARB1
NR1H2
HSP90AA1
NR5A2
PNPLA2
DPYD
GPBAR1
ABCG2
AREG
SMAD2
CDAN1
SUN2
IFI27
GCG
PCSK9
ENPP2
DNAL1
PTK2
SLC4A1
RNASEH2C
CASP9
KIF7
ITGAL
ENPP4
VWF
HGD
IL21R
GGTLC3
MT-CO2
CCNA2
GDF15
SULT2A1
CES2
ASGR1
AQP2
CDIN1
ERBB3
SDCCAG8
ASGR2
PLIN2
AGK
CD8A
SERPINB3

CDK2
AZIN1
AGT
MUC1
NAMPT
TIMP3
ONECUT1
ARHGAP31
DNMT3B
SNAI1
CYP2C19
HEPH
PCNA
PFKFB1
CDK1
TGFB3
SSTR2
MAN1B1
MMAA
NOTCH4
NPY
THBD
EGR1
MGST1
E2F1
POU5F1
TRMT6
AQP1
SMAD7
CHUK
IGFBP1
AVP
RNASEH2A
TNFSF11
HSPB1
PIK3CG
F10
DOCK6
ANGPTL8
RAG1
BSG
MMP14
RNASEH2B
SP1
ANGPT1
TNFAIP3
SLC51B
PPIG
ORMDL3
STN1
PKM
NME1
CLPX
TNFRSF6B
CXCR5
PEX11B
SAMHD1
ANGPTL3

PRMT7
SMARCA4
GCK
HLA-C
DPM2
GALC
WNT5A
NAGS
LTF
STAT6
ZMPSTE24
AXIN2
BBS12
LYRM7
HDAC1
SLC39A14
ANXA5
CCNB1
EPAS1
ANXA2
VKORC1
AKT3
CD86
IL12RB2
CFLAR
DKK1
TNFRSF10B
PRKCA
HSP90B1
TP73
RPGR
SLC25A37
TPMT
SERAC1
AGTR1
CD58
ARID1B
VEGFC
KLRK1
CYP27A1
PLCG1
ETS1
CCNE1
KLKB1
CEACAM1
SPARC
MDK
BBS5
CD1D
AURKA
SOS1
PSMD10
CDC25A
APOH
CREBBP
UBD
USP53
VIPAS39

CPQ
TTPA
SLC25A47
SHBG
CXCR1
ATF6
PRKN
EZR
RHOA
AIP
RPS6KB1
AKR1B10
FGL1
AURKB
PIK3C2A
CBL
HDGF
CPS1
HK2
PCCA
HPSE
ARID1A
RAC1
CDK6
TNFRSF10A
YARS2
PHF20
HADH
MAGEA4
UTS2
SFRP1
MX1
BECN1
RUNX3
SKP2
KEAP1
PINX1
ITGA5
WNT1
STMN1
PRKCB
PML
IRF3
LIG4
SMARCA2
NANOG
FLT3
IFNAR1
WNT3A
NR3C1
MGAT5
F13A1
M6PR
HPX
ALDH3A1
PNKD
TK2
IKBKG

PIGR
PRDM2
BBS7
CLEC4M
TWIST1
GLIS3
CLEC16A
BTNL2
USB1
GLYAT
PIK3CB
ERFE
UCHL1
CTCF
CYBRD1
CEP104
DPM1
TMEM176A
EIF2AK2
SHC1
CENPB
CTTN
STX1A
ATP11C
PXN
TMEM138
GPX3
DIABLO
KLF1
CREB3L3
GSTO1
DEPDC5
MAGEA3
PIK3R2
ICOSLG
IGF2-AS
ACTG1
RBP5
ZPBP2
MAGEA1
TCTN1
TMEM237
ZEB2
BAD
BIRC3
LEF1
DNAI2
HTATIP2
PLAT
TYK2
PSAP
ARSH
TFDP1
ACTC1
DCTN4
FN1
CASP7
TCTN3

GALM
SIRT3
C2CD3
LOX
WNT2
COPS5
MMP12
GAST
RECK
ACSL4
DRD4
STT3B
ATOX1
SRD5A3
HINT2
CSNK1A1
ASPH
CNDP2
GSTM3
TNFRSF11A
BBS10
PEG10
SERPINB1
SP140
YY1AP1
ERN1
GC
DDX58
IRF7
CLCA4
RUNX1
LGALS3BP
CD14
VTN
COL11A1
FGF7
FGFR2
LUM
CNR1
CNR2
COL8A2
AHR
F2R
HSD11B2
LOXL1
THBS1
SERPING1
RGN
SLC17A2
PEMT
CHRM3
CYP2R1
ADRA1A
COMMD1
CYBA
DHCR7
A2M
ANKS6

BAMBI
SLC13A4
HDAC2
IGFBP2
JUND
LAMC2
LGALS1
ARNT
ARRB1
PCOLCE
PLOD2
TRPM7
ACTA2
C1QB
TNFRSF1B
UNC93B1
TAGLN2
SERPINH1
CD9
SLC22A8
SPINT1
CYGB
PDIA3
PTN
RAD50
GCLC
MUC2
RGS2
PDGFD
AKAP12
MERTK
RNF7
FBL
FDFT1
EHBP1L1
CCL4
GLT8D2
CRACR2A
DDX60L
PAEP
CST3
RBM45
SLC25A1
MMRN1
GABPA
RSC1A1
MBOAT7
GGTLC1
CHI3L1
COX8A
ADRB2
VIPR1
CASP1
ZNF267
ZNRD2
DCTN6
CLTC
CYBB

CBLL2
EDNRA
EDNRB
FUT1
MSTN
GEM
GHR
GSTT1
TICAM2
KIR3DL1
MMP3
MMP8
MMP13
NGF
TMED7
PIK3CD
PSMD9
BCHE
RARRES2
OPN1SW
BMP7
SPINK1
ADAM17
TRIO
VIP
MUL1
HAVCR2
PCSK7
ZGLP1
SPRY2
KLF2
KHDRBS1
TNFSF13B
LYVE1
AZIN2
CKS2
CLU
MTFMT
SLCO6A1
CPE
CR1
CSF3
CYP2B6
DBP
DCN
DDX5
DDX53
BMPER
ATN1
EP300
EPHB2
EXTL3
F8
FCGR3A
FCGR3B
SYNM
METTL4
SMUG1

NUP62
IL27
GCA
GH1
NAAA
HPGDS
GLO1
GLP1R
UTS2R
BTBD8
GPX1
PYCARD
CXCL1
GTF2H1
PRMT1
HTR2A
TNC
GADL1
HSD17B13
GSTK1
SMIM1
LAIR1
LGALS3
STS
NR3C2
NAGLU
OSM
NOX4
IL22
SOST
GP6
TLR7
ISYNA1
GHRL
ARID4B
PECAM1
SERPINF1
PGF
PLA2G2A
AVPR2
PROX1
CHPT1
SLC12A9
BAGE
PTH
PTX3
BCR
SCN7A
CXCL11
SELP
SELENOP
SFRP2
XPO4
SOX9
TAP1
TFPI
POTEF
XRCC1

XRCC5
EHMT1
DHDDS
MINDY4
CAVIN2
SYVN1
AOC3
PDE5A
DCLK1
XPR1
KL
MUC16
RAPGEF5
TANK
SYCE1L
MICA
ABCB6
CDH11
ENAM
IFNL4
CDH13
FAM13A
CDK5
EIF1
CALCOCO2
SPRY1
DENND4A
CDK2AP2
MARCHF6
CDO1
ADARB1
PIAS3
IFITM3
GPNMB
FST
VAT1
CIB1
CXCR6
OGA
CEACAM3
PPP1R13L
CCL27
CEACAM7
CEACAM8
SUB1
RALBP1
SLC27A5
ADRM1
ESM1
RPP14
ZWINT
WIF1
AKAP13
CAVIN3
IL17F
NLRP3
CISH
NOSTRIN

CTHRC1
CKS1B
CLNK
DCD
SPPL3
RXFP2
OSCP1
ADH5
AADAC
COMP
CPB1
CLDN3
VPS37A
CRH
CSF1
OR2AG1
CSN3
CSTB
TMC4
RNF187
PLB1
CTSL
CX3CR1
ADRB3
CYP3A7
CYP4A11
CYP17A1
CYP19A1
CYP27B1
DGKB
DCC
DECR1
AFM
DMBT1
DMRT1
DNASE1
AGRP
SLC26A3
EIF2S3
ELAVL2
ETV3
ABCD1
FAP
FOLH1B
FBN2
MARCHF8
FCN1
FCN2
FDX1
FHL2
VASH1
MLXIP
SIRT2
PAXIP1
NT5C2
FOXC2
NCOA6
SETD1B

FOXO3
KIF1B
MCF2L2
TBC1D9
SULF1
FMOD
ANGPTL2
FOLH1
CABIN1
SRRM2
GSPT2
ALOX5
ALPI
BCL6B
HSPA12A
ASPM
WWTR1
SH2B1
CHMP2B
ATRNL1
CNTNAP2
GCDH
ADGRF1
GIP
USP21
NOX1
FOXP1
DKK3
GLB1
PDLIM3
RBMS3
GLI1
GLI2
HTRA2
GNRH1
ABO
LAMTOR2
GRIN2A
GRIN2B
MYLIP
CXCL2
GSN
GSTM2
TRPM5
ICOS
RMC1
NENF
HABP2
HARS1
HDLBP
HEXB
CFHR1
HGFAC
SOCS7
HIC1
UBE2K
HK3
HLA-DMA

HLA-DRB3
HMGB3
HMGCS2
AOX1
HOXA10
APEX1
HSF4
HSPA8
HSPA9
HSPG2
NDST1
HTR7
IAPP
ABCB5
NANOS1
NANOS3
MACC1
JCHAIN
APRT
IL1R1
IL9
IL10RA
TNFRSF9
IDO1
INSRR
IRF1
ISG20
ITGA2B
ITGAM
ITK
ITPA
ZNF699
ENHO
KCNJ5
KIR2DS4
KNG1
KIF11
ERPINA13P
LAG3
LALBA
LIF
LMNB1
CYP4F3
MIR17HG
MARCKS
SMAD1
ARRB2
MAS1
MAT2A
MATN2
MCAM
MCC
MCM2
MEF2A
MFAP1
MFGE8
MIF
ATXN3

MLN
ACHE
MSMB
MSX2
MTAP
MTR
NCAM1
ATIC
NELL1
NFATC1
NFE2
NGFR
NOS1
NOTCH3
CCN3
NPPB
NT5E
NUP98
OAS1
TNFRSF11B
OPRD1
PC
DERL2
GMNN
ANGPTL4
JPT1
MARCHF2
THEM6
PLA1A
TRPV2
PDE4A
EVL
PDE1B
SF3B6
CD244
PHB
PHEX
PIN1
PLA2G1B
PLA2G4A
IL17D
PLG
PLXNA2
PRRX1
NANS
POMC
ATG16L1
PIWIL2
PPP2CA
NAT10
NUDT15
HES6
UBE2Q1
PRKAA2
TENM3
ALLC
PAG1
MYDGF

PRL
PRLR
PROC
PROS1
AZGP1
CCL28
KLK10
AZU1
PSG2
PNO1
TWSG1
PSMC5
PDSS2
RTN4
PTBP1
PTGDS
AICDA
MAVS
SEMA6A
KLHL1
CIP2A
QSOX1
ZNF410
CXCL16
SCAF1
RAB27A
IL21
RASGRF2
RBP1
BCL3
RNH1
RPS27A
RRM2
BDNF
S100A9
SAA1
SAA2
CFB
TSPAN31
CCL14
CXCL5
SDC1
SH2D4A
PRDM1
GOLPH3
VSIR
SFRP5
SRSF3
SRSF5
HHIP
GORASP1
SMURF2
ST6GAL1
BMP1
WNK1
SLC6A3
SFTPA1
SLC11A1

SMARCA1
SIGLEC1
SNCA
SNCG
SOX1
BPI
SPTBN1
SRY
ST14
SYK
SYT1
KLF9
TAP2
TAZ
MSMP
TDGF1
TDGF1P3
TEK
TEP1
TGFBI
TGM2
THBS4
THY1
KLF10
TPO
TRAF6
TRH
CCR2
TYR
UMOD
VPS51
UQCRFS1
USF2
VCAM1
TRPV1
YWHAZ
ZKSCAN1
CA9
ZNF148
DDR1
MANF
ST8SIA4
FSD1
BIRC7
TNFAIP8L2
DENND2D
NR4A3
ASRGL1
CPEB4
WNT10A
ADAM12
PDCD1LG2
SHARPIN
ARHGAP24
NUF2
BCL2L12
FSD1L
IMMP2L

PLA2G6
TMPRSS13
MAGT1
SARNP
MAK16
CASR
SMARCA5
TRIM63
GPT2
DGKE
NUMB
GPAA1
RIPK1
IQGAP1
NRP2
NRP1
APLN
SPHK1
EIF2S2
CCNA1
P4HA2
LMLN
HSPB3
UNC5A
ARTN
ARHGEF1
HGS
FIBP
BOC
CD5L
CYTH2
CD163
SLIT2
LHX2
SFXN1
TNFRSF8
AIM2
GGPS1
ENTPD1
CIR1
[ACROH2A1
CD47
KLK4
CD63
PPIP5K1
CD68
CD69
HERPUD1
ACYP2
MFN2
NR1D2
ALPL
FMO2
AGXT
LEAP2
LARS1
COX7A2
RINT1

RNASE2
PFKL
SLC25A6
P4HA1
