# Supplementary material for: Network Pharmacology-Based Exploration on the Intervention of Qinghao Biejia Decoction on the Inflammation-Carcinoma Transformation Process of Chronic Liver Disease via MAPK and PI3k/AKT Pathway
Source: Biomed Res Int. 2022 Oct 14;2022:9202128. doi: 10.1155/2022/9202128 (PMC9586778; doi:10.1155/2022/9202128)
Supplement: Supplementary Materials — Supplementary data associated with this article can be found in the appendix. Supplementary file 1 shows the compounds and their associated targets for QBD and Supplementary files 2-8 show the associated targets for CLD. [file 9202128.f1.zip › Supplement 1-Compoud.pdf]

ID	PubChem Name
AAH1	Isobornyl acetate
AAH2	isoquercetin
AAH3	(-)-alpha-Terpineol
AAH4	1,8-Cineole
AAH5	(-)-Isoborneol
AAH6	salicylic acid
AAH7	(-)-Bornyl acetate
AAH8	(-)-Linalool
AAH9	Scoparone
AAH10	(-)-cis-Carveol
AAH11	(+)-Isoborneol
AAH12	Eupatin
AAH13	Rhamnocitrin
AAH14	3-O-Methylquercetin
AAH15	l-alpha-Fenchone
AAH16	isorhamnetin
AAH17	Esculetin
AAH18	Benzyl isovalerate
AAH19	scopoletin
AAH20	Tamarixetin
AAH21	Patuletin
AAH22	coumarin
AAH23	Stigmasterol
AAH24	Arcapillin
AAH25	(-)-trans-Carveol
AAH26	(-)-alpha-terpinyl acetate
AAH27	Artemetin
AAH28	Phytodolor
AAH29	Artemisia ketone
AAH30	Rhamnetin
AAH31	Luteolin
AAH32	(+)-Terpinen-4-ol
AAH33	Chlorogenic acid
AAH34	(-)-Camphor
AAH35	β-terpineol
AAH36	Skrofulein
AAH37	Artemisitene
AAH38	Dihydro-epi-deoxyarteannuin B
AAH39	Deoxyarteannuin B
AAH40	Chrysosplenol D
AAH41	7-methylidene-2,3,4,4a,5,6,8,8a-octahydro-1H-naphthalene-1-carboxylic acid
AAH42	methyl-2-methylidenebicyclo[3.1.1]heptan-3-one
AAH43	Cirsiliol
AAH44	ıenyl-3-(1 -nonen-3,5 ,7-triynyl)oxirane
AAH45	Vitexin
AAH46	Cirsilineol
AAH47	Umckalin
AAH48	,3'-Tetrahydroxy-3,4'-dimethoxy flavone
AAH49	4'-methylpenduletin
AAH50	Artemisinin B
AAH51	Artemisiaketon
AAH52	DMQT
AAH53	mido-3-phenylpropanoyl]amino]-3-phenylpropyl] acetate
AAH54	Penduletin
AAH55	)ihydroxy-6,7,3',4'-tetramethoxyflavone
AAH56	gamma-Terpineol
AAH57	Quercetagetin 3,4'-dimethyl ether

AAH58	artemisinin
AAH59	Dihydroartemisinin
AAH60	Deoxyartemisinin
AAH61	bornyl isovalerate
AAH62	Artemisinic acid
AAH63	-3-methylidenedecahydronaphtho[1,8-bc]pyran-2(3H)-one
AAH64	Quinghaosu I
AAH65	dimethyl-1,2,3,4,4a,5,6,8a-octahydronaphthalen-1-yl]prop-2-enoate
AAH66	deoxyisoartemisinin b
AAH67	Artesunate
AAH68	cid, 3-methylene-, 1,7,7-trimethylbicyclo[2.2.1]hept-2-yl ester
AAH69	methyl-1,2,3,4,4a,5,6,8a-octahydronaphthalen-1-yl]propanoic acid
AAH70	isoaromadendrene,epoxide
AAH71	trans-Pinocarveyl acetate
AAH72	Apigenin
AAH73	Hyperin
AAH74	sitosterol
AAH75	kaempferol
AAH76	quercetin
TC1	Valine
TC2	tyrosine
TC3	Proline
TC4	Phenylalanine
TC5	pentose
TC6	Methionine
TC7	Mannose
TC8	L-Threonine
TC9	L-lysine
TC10	Leucine
TC11	Isoleucine
TC12	hydroxyproline
TC13	glutamic acid
TC14	Glucuronic acid
TC15	glucose
TC16	Galactose
TC17	D-GALACTURONIC ACID
TC18	D-Galactosamine
TC19	Aspartic acid
TC20	Arginine
TC21	Alanine
TC22	2-amino-2-deoxy-D-glucose
ARR1	(-)-alpha-Terpineol
ARR2	asperglaucide
ARR3	Methyl gallate
ARR4	(+)-Borneol
ARR5	Tingenone
ARR6	Nyasol
ARR7	Sarsasapogenin
ARR8	Smilagenin
ARR9	nicotinic acid
ARR10	Anhydroicaritin
ARR11	Neogitogenin
ARR12	Pantothenic acid
ARR13	Tigogenin
ARR14	Ginkgoic acid
ARR15	Hippeastrine
ARR16	12-O-Nicotinoylisolineolone

ARR17	(+)-Nicotine
ARR18	Smilagenone
ARR19	Timosaponin A III
ARR20	Timosaponin A-I
ARR21	markogenin
ARR22	Icariin I
ARR23	Anemarrhenasaponin-Ia
ARR24	n-cis-Feruloyltyramine
ARR25	(-)-Caryophyllene oxide
ARR26	Diosgenin
ARR27	coumaroyltyramine
ARR28	hexanal
ARR29	Nonadienal
ARR30	sitosterol
ARR31	kaempferol
ARR32	Stigmasterol
RR1	s-p-hydroxy cinnamic acid methyl ester
RR2	salidroside
RR3	rehmapicrogenin
RR4	3-indolecarboxylic acid
RR5	rehmaglutin d
RR6	rehmaglutin c
RR7	rehmaglutin b
RR8	rehmaglutin a
RR9	pterolactam
RR10	gamma-aminobutyric acid
RR11	ferulic acid methyl ester
RR12	echinacoside
RR13	diincarvilone A
RR14	daucosterol
RR15	coniferin
RR16	catalpol
RR17	acteoside
RR18	5-hydroxy-2-pyridinemethanol
RR19	10-de-oxyeucommiol
RR20	sitosterol
MC1	PHB
MC2	vanillic acid
MC3	1S,5R)-6,6-Dimethylnorpinan-2-one
MC4	Trochol
MC5	paeoniflorin
MC6	hexanoic acid
MC7	Diethyl phthalate
MC8	Betulinic acid
MC9	Benzoic acid
MC10	Oleanolic acid
MC11	caffeic acid
MC12	eugenol
MC13	Acetovanillone
MC14	sitosterol
MC15	6-Hydroxycoumarin
MC16	kaempferol
MC17	Gallic acid
MC18	2',4'-Dihydroxyacetophenone
MC19	methyl salicylate
MC20	R)-3,4,5-trihydroxy-6-(hydroxymethyl)oxan-2-yl]oxyphenyl]ethanone
MC21	3-Hydroxy-4-methoxyacetophenone
MC22	3-dihydroxy-4-methoxyphenyl)ethanone

MC23	[illegible]henyl)-furan-2-ylmethylene]-pyrimidine-2,4,6-trione
MC24	3-acetyl-4-hydroxybenzoic acid
MC25	paeonol
MC26	Schottenol
MC27	quercetin

881Target of compound

ACHE
VDR
CDC25A
PTPN1
HMGCR
CYP19A1
UGT2B7
ADRA2A
ADRA2C
ADRA2B
ADRA1A
TBXAS1
CYP11B1
CYP11B2
HSD11B1
POLA1
TARR1
CTRB1
PABPC1
LIPE
CA1
TYMS
CHRNA4
KCNK2
HTR1A
KCNH2
CA2
AKR1B1
CA7
CA12
CA4
NOX4
NQO2
RPS6KA3
NMUR2
PTGS2
CD38
PDE5A
TNF
IL2
ADORA1
XDH
ALOX5
SLC29A1
TERT
AR
TRPM8
NR1H3
CHRM2
NR1I3

BCHE
SLC6A4
SREBF2
NPC1L1
SQLE
ESR1
SLC6A2
DRD2
PTPRF
PTPN2
PLA2G1B
ACP1
AKR1B10
ESR2
CYP51A1
ATP12A
PTPN6
CYP17A1
CYP2C19
FABP4
PPARG
PPARA
FABP3
FABP5
PPARD
FABP1
RORA
NR3C2
CD81
SIGMAR1
NR3C1
G6PD
SCD
SHH
CDC25B
NR1H4
GPBAR1
SHBG
GABBR1
GABRA2
POLB
CA9
CA14
CA6
CA3
CA5B
CA5A
CA13
LDHA
LDHB
ERN1
AKR1C2
AKR1C1
HDAC6
HDAC8
DPP4
FUT7
DAO

ALB
MCL1
TRPV3
PGR
HSD17B2
HMOX1
IDO1
SLC6A3
CHRM4
OPRM1
OPRD1
OPRK1
KCNA5
SCN5A
SCN9A
PTAFR
PARP1
JAK1
JAK2
LRRK2
MAPK8
LTA4H
AKR1C3
TNNC1
UTS2R
EGFR
SRD5A1
CBR1
SRC
MAOA
MAOB
GSK3B
KCNA3
IGF1R
KDR
AURKA
ERBB2
PDE3A
PDE3B
GPR35
FGR
LYN
ADAMTS5
BACE1
PARP2
MPO
AURKB
CDK2
TYMP
GRM4
CDC7
IKBKB
KCNN1
KCNN3
KCNN2
CTSK
CTSS
CTSL

PDGFRB
FLT4
INSR
TEK
EPHB4
CES1
MAPK10
CES2
EPHB3
PLK1
MAP2K3
BTK
SYK
MAPK14
ROCK2
CLK4
RPS6KA5
RPS6KB1
PIM2
PBK
PIM3
NTRK3
PRKX
MAP4K4
BRAF
PTPRC
MAP3K14
TYK2
DYRK1B
MET
ICAM1
SELE
TGM2
PTPN13
KDM4C
METAP1
APEX1
NUAK1
MAPKAPK2
MPI
GRM5
TOP1
CYP1B1
PLG
MAPT
KDM4E
AVPR2
TOP2A
DRD4
GLO1
PIK3R1
DAPK1
PYGL
ABCC1
PKN1
CSNK2A1
NEK2
CAMK2B

ALK
AKT1
NEK6
AKR1C4
AKR1A1
ABCG2
CCNB3
CDK6
ARG1
ADORA3
APP
MMP3
MMP2
PIK3CG
TYR
AHR
ESRRA
FLT3
ADORA2A
PTK2
MMP13
MMP9
ALOX12
ST6GAL1
HSD17B1
PTPRS
MPG
SLC22A12
AXL
ABCB1
ODC1
PFKFB3
F2
TNKS2
TNKS
MYLK
ALOX15
PIM1
CXCR1
PLA2G2A
CDK1
CDK5R1
TTR
CFTR
AMY1A
GRK6
NAE1
MMP12
SERPINA6
HSD17B3
CCND1
PLK4
MAP3K8
HSPA1A
GSR
CCNE1
PTK2B
MIF

SNCA
ALDH2
PRSS1
PRKCD
C1R
PDE10A
PDGFRA
CTSH
FKBP1A
PSMB1
GABRB3
PLA2G7
CHRM3
CACNA1C
P2RX7
TSPO
GRM2
ALDH3A1
FADS1
PIK3CD
CRHR1
PIK3CB
PIK3CA
CXCR2
TGFBR1
IDH1
DHFR
NPEPPS
GLP1R
TRPA1
CCR4
EGLN3
FAAH
KCNJ5
PLA2G6
ADORA2B
HTR6
TMIGD3
HTR2B
DRD1
HTR2A
TACR2
DRD3
ADRB3
CYP2D6
CYP2C9
CYP3A4
CTSG
FNTA
CHRM5
TBXA2R
QPCT
CYP1A2
NAT1
MB
KCNMA1
COMT
CDK9

ALPG
PLAA
CHRM1
AOC3
CISD1
KIT
NFKB1
NQO1
RORC
PTGER1
PTGER2
NR1H2
PTGES
DHCR7
FDFT1
NOS2
CTRC
CTSB
EPHX1
LSS
TAS2R31
ELANE
XPO1
CYP2A6
HSD11B2
EPAS1
CEL
NLRP3
HIF1A
PRKDC
NOS1
NOS3
GPR55
HTT
ADRA1D
RET
GSTA1
NISCH
ALPL
HDAC1
ADH1A
ADH1C
HTR7
SLC37A4
PRKCA
PDE4D
PDE9A
PDE1B
EDNRA
PTGS1
SAE1
LCK
CALM1
MTNR1A
MTNR1B
MAPK9
IRAK4
GUSB

PRKCG
JAK3
PDE4B
GABRA1
GABRA5
MALT1
CPT1A
IRAK1
KDM1A
GSK3A
IL1B
CDC25C
BCL2L1
JUN
TTL
NR1I2
PCSK7
F2RL1
ITGAL
PREP
CNR2
STAT3
PRKCE
PRKCH
PTPN11
HCAR2
CPA1
TRPV1
MAPK1
NPY5R
CYP26A1
PLEC
CSNK1A1
CSNK1D
HSP90AA1
HDAC2
HDAC5
HDAC7
HDAC4
PTGFR
PTGER3
PTGDR
PTGIR
LTB4R
PTGER4
KMT5A
CTNNB1
MME
HAO2
SLC22A6
ACE
FBP1
PTGDR2
MGLL
CCR3
F10
HLA-DRB1
MMP1

PSMB5
CBX7
ITGA2B
PSENEN
SOAT1
GHSR
CTSV
CCKBR
FLT1
FGFR1
CMA1
CAPN1
S1PR3
PDPK1
MDM4
PDE4C
TBK1
MAP2K1
CDK8
DYRK1A
PDE2A
RHOA
TGM1
F13A1
PAM
GPR183
CBX8
CBX4
SLC7A11
TYRO3
VDAC2
NTSR1
MMP8
TSHR
ALOX5AP
SFRP1
SLC27A1
SLC8A1
WNT3
NAMPT
BRD4
PTK6
PPIA
GSTP1
TLR9
CASP1
BRS3
ACPP
CASP7
EDNRB
ABCC9
OXTR
LGMN
CASP8
PSEN2
PSMB2
SLC5A1
MAPK3

GLRA1
GLRA2
ASAH1
CSF1R
DUSP3
KCNJ11
GRK2
ADAM17
KCNE1
ICMT
P2RX3
EWS-Fli1
CREBBP
SRD5A2
FFAR1
CNR1
SLC2A1
ADCY1
LIPA
STK3
STK26
EPHX2
PER2
IL6ST
RASGRP1
MDM2
DNTT
CCR5
CTSC
CTSF
ATP4B
CASP3
PTGIS
CASP9
CASP6
PDE6D
IMPDH1
IMPDH2
GZMB
DGAT1
HNF4A
THRB
EP300
ITGAV
ITGB1
CPT1B
PRKAG1
PRKAA1
THRA
CPT2
DDR2
EGLN1
STS
SERPINE1
STAT6
HCRTR2
HCRTR1
CHRNA3

MCHR1
P2RY1
CTSD
CCR2
NUDT1
PGGT1B
HTR2C
GABBR2
SLC6A1
GABRA3
GABRR1
OAT
SLC6A11
SLC6A13
HDAC3
CACNA2D1
GRM8
GRM7
FYN
TH
SLC7A5
PTPRA
KMO
PEPD
GRB2
CPB1
KIF11
REN
ANPEP
TACR1
CPB2
ENPEP
CPA3
SLC15A1
GRIA1
GBA
VEGFA
FGF1
FGF2
HPSE
RNPEP
KYNU
BHMT2
LGALS4
LGALS3
LGALS8
PYGB
ABAT
GRIK1
GRIK2
GRM1
GRM3
GRM6
SLC1A1
GRIK5
GRIA4
GRIK3
GRIA2

SLC1A2
SLC6A12
BBOX1
HTR1B
PPM1A
MGAM
SI
BRD9
SCN10A
BCL2
CYP1A1
CETP
NTRK1
MAPK11
MKNK2
SLC33A1
CCNA2
KCNJ6
PLAUR
TACR3
HSF1
CYP24A1
G6PC
CHEK1
WEE1
SPHK2
SPHK1
AVPR1A
GLI2
GLI1
FASN
F3
CYSLTR1
ESRRB
CLK1
MTOR
CHRNA7
F2R
SMO
COL4A3BP
HPGDS
HSD3B2
OPRL1
DDO
SIRT3
SIRT2
HSP90AB1
PDK1
HSP90B1
PLAU
CALCA
GPR84
GCGR
MELK
CNOT7
ADRB2
ADRB1
CCR1

OGA
ECE1
FOLH1
AMPD3
DNMT3B
KAT2B
KAT5
AGTR1
UBLCP1
UPP1
MTAP
FUCA1
PNP
MAN1B1
MAN2A1
HRH1
DPP7
HRH3
PRKCI
SLC18A3
PDE11A
DPP8
FAP
ACKR3
MMP7
PIK3C3
ACVRL1
TKT
LIMK2
CLK3
DYRK2
CCNC
GCK
CCNT1
CFD
EZH2
SORD
AKT2
CHEK2
TAB1
CDC42BPA
AKT3
PLK3
MAP3K7
PTPA
ROS1
CHRNB1
CHRNB4
CHRNB3
CHRNA2
SLC18A2
S1PR1
ABL1
ACACB
PRKCB
PPP1CC
PPP2CA
F7

EPHA2
YES1
BLK
CSK
EPHB2
BMX
EPHA5
EPHA4
TXK
EPHA6
EPHA3
COQ8B
EPHA1
PRKCZ
BMP1
HTR3A
MMP16
MMP14
DNM1
SF3B3
NCOR2
NCOR1
HDAC11
CDK4
VCP
NPY1R
NEK1
ROCK1
PRKACA
CALCRL
MMP15
MMP26
RASGRP3
MC1R
DCTPP1
MAP4K2
TAOK1
TAOK3
STK4
FOXO1
CCNE2
LNPEP
PITRM1
ADH1B
ADH4
SLC5A7
ALDH1A1
ADH7
NR4A1
TLR4
CBFB
QDPR
SLC16A1
RPS6KA2
ILK
MKNK1
ADA
ADK

CDA
HRAS
FTO
GSTM1
ATP2A1
IARS
PRKCQ
VAV1
TRPV4
PDCD4
PYGM
HEXA
HEXB
NFE2L2
ASF1A
DBF4
SIRT1
MERTK
PAK1
GYS1
MAP2K4
MAPK13
PPM1B
PPP2R5A
IGFBP3
SLC5A2
GAPDH
LGALS9
HSPA8
HK2
HK1
GRK1
AMY2A
TREH
HPRT1
LGALS1
AHCY
TPMT
KDM4A
KDM3A
KDM6B
KDM2A
TUBB1
SSTR5
SSTR2
SSTR4
SSTR1
SSTR3
SELP
FABP2
PHF8
KDM5C
HAO1
GSTK1
CDC45
NAAA
KLKB1
METAP2

TDO2
TLR7
PTPN22
ALDH5A1
RNASEH1
SLC5A4
SLC28A3
GAA
HSPA5
TK1
DTYMK
BAD
BDKRB1
SIRT5
PKM
ATIC
RARG
RARB
GPR17
