# Supplementary material for: Network Pharmacology-Based Exploration on the Intervention of Qinghao Biejia Decoction on the Inflammation-Carcinoma Transformation Process of Chronic Liver Disease via MAPK and PI3k/AKT Pathway
Source: Biomed Res Int. 2022 Oct 14;2022:9202128. doi: 10.1155/2022/9202128 (PMC9586778; doi:10.1155/2022/9202128)
Supplement: Supplementary Materials — Supplementary data associated with this article can be found in the appendix. Supplementary file 1 shows the compounds and their associated targets for QBD and Supplementary files 2-8 show the associated targets for CLD. [file 9202128.f1.zip › Supplement 2-HCC target.pdf]

HCC target（Results after deleting duplicate targets）

ECHS1
ADH1B
GPC3
ALB
BHMT
PLG
VIM
RGN
TF
FABP1
HPD
ACADSB
CAT
MTHFD1
RPSA
SLC22A1
TDO2
ADH4
CP
CYP2E1
PCK1
SPARC
ACSL1
ALDH1L1
ALDH4A1
ALDOB
CPS1
CYB5A
CYP2C9
FGB
FGG
GAPDH
HRSP12
HSD17B10
HSPA5
IGFBP3
PCK2
PDIA3
SERPINA1
TTR
AGXT
AKR1B10
AOX1
APOA1
ARG1
CCT3
CTSB
CYP2C8
FAH
FGA
FGL1
FTCD
GHR
HSPB1
IGF2
KRT8
NDRG1

S100A10
SCP2
SELENBP1
SULT2A1
UBD
ACAA1
ACAA2
AFP
ALDH2
ANXA2
BUB1
CLIC1
COL1A2
COMT
CYP2B6
DCXR
DNASE1L3
FEN1
FN1
GLUL
HMMR
HNRNPA2B1
HSP90B1
ITIH4
KHK
MT2A
NNMT
NPM1
PCSK6
PGK1
PPIB
PRDX6
RFC4
SLC2A2
SNRPE
STMN1
TOP2A
A2M
ACAT1
ACY1
ALDH1A1
APOC3
ARID3A
ASS1
C6
C9
CA2
CAP2
CCT5
CD14
CD24
CDK4
CDKN1A
CES1
CES2
CLU
COL1A1
CPB2

CTGF
CYP27A1
CYP2J2
CYP3A4
EFNA1
EPHX1
ETS2
FABP5
FH
GC
GNMT
HAAO
HDGF
HGD
HMGCS2
HSD17B4
HSPA1B
HSPA8
IGKC
ISG15
KNG1
LAMP2
MAT1A
MCM2
MMP9
NME1
PCNA
PEMT
PGRMC1
PKM2
PON3
QDPR
RBP1
RDBP
SCAMP3
SERPING1
SOD1
TST
TUBB
VTN
ACADVL
ACTB
ADH1C
ADH6
AKR1C2
ALAS1
ALDH1B1
ALDH3A2
ALDOA
B2M
BNIP3
C1R
C5orf13
C8A
CAPG
CBX1
CFH
CRHBP

CSTB
CTH
CXCL12
CYP1A2
CYP2A6
CYP2A7
CYR61
DDR1
DLGAP5
ENO1
FCGRT
FCN3
GLUD1
GRHPR
GYS2
HAMP
HAO1
HGFAC
HMGA1
HNRNPC
HPX
HSD17B6
IFI27
IFIT1
IGF2R
ILF2
ITIH2
ITPR2
KIF23
LPGAT1
LYZ
MAD2L1
MCM6
MDK
MT1F
NAMPT
NFKBIA
PC
PEG10
PGM1
PRDX1
PROZ
PTTG1
PUF60
PYGB
RBP4
RCAN1
RHOA
RHOB
RND3
RPLP0
RPS5
S100A6
SAA2
SERPINC1
SERPINF2
SGK1
SLC16A2

SLC7A2
SOD2
SPARCL1
SRGN
STAT1
TGM2
THBS1
TIMP1
TMED2
TPM2
UGT2B7
TP73
RUNX3
SFN
KHDRBS1
EIF3I
YBX1
CDKN2C
LEPR
IL12RB2
BCL10
GNAI3
RHOC
SYCP1
MCL1
PSMD4
S100A9
JTB
SHC1
MUC1
YY1AP1
COPA
NCSTN
ATF6
PTGS2
RGS1
KISS1
PARP1
MTR
ADAM17
DNMT3A
GPR75
RTN4
TGFA
REG1A
REG3A
MAT2A
IL18RAP
STEAP3
CD302
ITGA6
ERBB4
RAF1
XPC
TGFBR2
CTNNB1
CDC25A
COL7A1

SEMA3B
RASSF1
ZMYND10
FHIT
ROBO1
MME
TNFSF10
PIK3CA
TFRC
FGFR3
GPR78
PDGFRA
UGT2B4
AFM
CXCL2
HPSE
PTPN13
SPP1
ABCG2
EGF
CCNA2
IL2
FGF2
ANXA10
VEGFC
CASP3
KLKB1
TERT
APC
IRF1
HSPA4
NRG2
HBEGF
FGF1
PTTG2
DUSP1
STC2
BLOC1S5
EDN1
DEK
HFE
PGC
VEGFA
CCN2
ESR1
ACAT2
PRKN
HOXA13
IGFBP1
EGFR
CLDN4
GNAI1
HGF
ABCB1
MCM7
SERPINE1
DOCK4
MET

LEP
SMO
ZYX
EZH2
SHH
ANGPT2
DLC1
VPS37A
MTUS1
STC1
NRG1
SNAI2
ASPH
COPS5
STMN2
CA1
CA3
CDH17
LAPTM4B
ANGPT1
EBAG9
EIF3H
ENPP2
HAS2
ZHX2
MTSS1
MYC
PTK2
LY6E
GPAA1
RLN1
MTAP
CDKN2A
CDKN2B
TEK
GADD45G
PTCH1
NEK6
ENG
LCN2
KLF6
TRDMT1
ITGB1
RET
PLAU
NRG3
SNCG
FAS
TCF7L2
OAT
MKI67
MGMT
CD81
CDKN1C
SLC22A18
LYVE1
CD44
CD82

BAD
GSTP1
CCND1
FADD
FUT4
MMP7
MMP3
MMP12
CASP1
FKBP4
CD9
CD163
YBX3
CDKN1B
SLCO1B1
KRAS
LETMD1
ERBB3
GLI1
TSPAN8
LGR5
DCN
IGF1
GJB2
CRYL1
HSPH1
TNFSF11
RB1
EDNRB
SPRY2
DAD1
MMP14
SSTR1
RPL36AL
LGALS3
DACT1
SGPP1
SLC10A1
FOS
HSP90AA1
MTA1
SPRED1
SPINT1
PCLAF
MAP2K1
CIB2
AKAP13
ARRDC4
AXIN1
SSTR5
GFER
SOCS1
PLK1
TAOK2
MAPK3
ZNF689
PYDC1
SIAH1

RBL2
MMP2
CDH1
HP
WWOX
VPS53
DERL2
ACAP1
TP53
NOS2
ERBB2
RARA
KRT19
ETV4
GRN
DYNLL2
AXIN2
SSTR2
SLC9A3R1
BIRC5
PSMG2
SMAD2
SMAD4
DCC
SERPINB2
CDC34
KISS1R
GADD45B
MAP2K2
CREB3L3
SAFB
PIN1
DNMT1
ICAM1
ACP5
JUNB
PRDX2
CCNE1
SPINT2
LGALS4
AKT2
GSK3A
ETHE1
APOE
ZNF296
ERCC1
BAX
FTL
CDC25B
ID1
DNMT3B
MAPRE1
E2F1
SRC
MYBL2
CD40
SNAI1
AURKA

GNAS
TPTE
BAGE5
TIAM1
TFF3
CBS
UBE2L3
MAPK1
MIF
SSTR3
MLC1
TYMP
CD99
DDX3X
SSX5
SSX1
GAGE2D
AR
EFNB1
GJB1
PSMD10
LUZP4
F9
SPANXC
MAGEC2
MAGEA11
MAGEA8
FATE1
MAGEA4
GABRE
MAGEA10
MAGEA2
MAGEA3
MAGEA1
CTAG1A
RBMY1B
PPIA
ARMC10
PTEN
HEPACAM
HEPN1
ST13
SSX4
SSX2
TSPY1
