# Supplementary material for: Network Pharmacology-Based Exploration on the Intervention of Qinghao Biejia Decoction on the Inflammation-Carcinoma Transformation Process of Chronic Liver Disease via MAPK and PI3k/AKT Pathway
Source: Biomed Res Int. 2022 Oct 14;2022:9202128. doi: 10.1155/2022/9202128 (PMC9586778; doi:10.1155/2022/9202128)
Supplement: Supplementary Materials — Supplementary data associated with this article can be found in the appendix. Supplementary file 1 shows the compounds and their associated targets for QBD and Supplementary files 2-8 show the associated targets for CLD. [file 9202128.f1.zip › Supplement 3-NAFLD target.pdf]

NAFLD target（Results after deleting duplicate targets）
TNF
IL6
INS
PNPLA3
PPARG
IL1B
GGT1
TLR4
HFE
IL10
LEP
FAS
TGFB1
NR1H4
AKT1
SLC17A5
APOB
APOE
TP53
HADHA
ALB
CAT
IRS1
APOA1
MTTP
RETN
INSR
LEPR
MLXIPL
SERPINE1
RBP4
UCP2
DDIT3
LPL
NR1H3
GPT
ABCB11
CCL2
PPARGC1A
ADIPOQ
IGF1
HMOX1
PPARA
IRS2
TLR2
CD36
CRP
HNF4A
AHSG
PTEN
MTOR
CXCL8
ACACA
HGF
CYP2E1
F2
APOC3

SOD2
SOCS3
CPT2
IKBKB
IFNG
KRT18
FASN
EIF2AK3
SLC2A4
CASP3
SREBF1
AFP
IL1A
CEBPA
MAPK8
FABP1
SIRT1
VEGFA
CCN2
CTNNB1
SMPD1
CTLA4
SERPINA1
LMNA
STAT3
FABP4
ACE
PIK3CA
MFN2
RELA
ATP7B
GHRL
ABCA1
MTHFR
FASLG
TM6SF2
MSR1
EGFR
MARS1
XBP1
MMP9
ICAM1
CFTR
LIPA
TNFRSF1A
APP
ACOX1
CPT1A
NDUFS1
ABCB4
ADIPOR1
CAV1
TIMP1
IL18
ADIPOR2
STAT1
JUN
CASP8

CP
LCAT
MAP3K5
CDKN2A
MMP2
ATF4
CYCS
HMGCR
TTR
NOTCH1
TF
MPO
MYC
FGF21
LDLR
TERT
SREBF2
PON1
IL1RN
NFKB1
EGF
VWF
SMAD4
JAG1
SQSTM1
SLC25A13
PIK3R1
ESR1
CCND1
NOS2
BSCL2
AKT2
CETP
VCP
GANAB
G6PC
SPP1
SERPINC1
GCG
BAX
HAMP
ABCC2
HLA-DQB1
BDNF
VIM
CYP3A4
PTPN11
GCKR
ELANE
DPP4
MMP1
CDKN1A
NDUFS8
CXCL10
HSPB1
MT-CO1
PCSK9
POMC

LIPC
PRMT7
PRKAG2
CXCR4
TNFRSF1B
SLC2A1
NOD2
THBD
NAMPT
NLRP3
BMP6
PNPLA2
MT-CYB
PDGFRB
SDHB
IGF1R
NGF
CRYAA
ACTB
PKLR
HSPD1
SCD
BCL2
PTGS2
TLR9
SERPINA3
ERN1
C3
TLR1
TGFBR2
DRD4
TLR6
CFH
SLC6A3
TNFSF11
ENPP1
LIPE
UGT1A1
FGF19
AGPAT2
MDM2
CCL3
ABCB7
DNMT3B
DNAJB11
SDHC
CDK4
FLT1
ASS1
LPA
SLC6A4
FN1
PRKAB1
PRKAA1
CTSD
CDKN3
APOA5
IL17A

TARDBP
CYP7A1
GRN
AKT3
CASR
PLAU
GCK
KRT8
ANO5
TGFBR1
EZH2
IFIH1
MT-CO2
PC
XDH
ALMS1
MYD88
GSR
FABP2
GH1
CHUK
HBB
SMARCA4
CYP1A1
SIRT3
CCL4
FOS
FGF23
EP300
PIK3R2
VDR
LOX
CTSB
GSK3B
AKR1D1
IDH2
NR1H2
F3
RAC1
GARS1
COX5A
APOA2
MT-ND6
CDH2
HTR2A
PLIN1
CRAT
MT-CO3
PIK3CD
TNFAIP3
EIF2S1
CPOX
CDC42
SLC40A1
GSTM1
CYP1A2
GSTP1
HIF1A

SPINK1
CCL5
SUMF1
AGTR1
CHI3L1
CYBB
SMAD7
COX6B1
BGLAP
CHIT1
NFE2L2
DGAT1
KRT7
ELOVL6
SERPINA6
TFR2
CRH
RXRA
BMPR1A
ERBB4
ALPP
TNFRSF11B
CYP17A1
PLA2G4A
VCAM1
LRP6
SERPINF2
ETS1
HSD11B1
FGFR4
LCN2
ADH1C
ALPL
SHBG
MYF5
DSP
CA2
FABP3
KRT19
CD55
IFNA2
SELE
CNR1
MAPK10
HP
ERCC2
HUWE1
MAPK14
AGT
UQCRC2
CD44
IRF1
WNT3A
GLUD1
ALDH2
SELP
HJV
SMAD3

PEMT
IL6R
TNFSF10
ADH7
MT-ND3
CYP1B1
NPPB
AIFM1
MYH9
SLC27A1
CYC1
FADS2
SCARB1
CEL
UCP1
MMP13
ADA2
NR1I2
LRPPRC
HNRNPA1
PRKAA2
CPS1
TIMP2
PRSS1
CYBA
IGFBP3
CYP2A6
ADRB3
FLI1
SHH
HSP90AA1
PRL
PAPOLG
UQCRQ
COX8A
FGA
SLC27A2
IL15
LITAF
CSF1
IL1R1
GC
STAR
NPY
CES1
COX4I1
AHR
FABP12
IFNL3
CLU
DDX3X
DKK1
SLC10A1
CXCR3
CASP7
S100A9
CASP9
TET2

RARS1
BIRC5
HSPG2
XRCC4
CLCN2
PPIG
SP1
GHR
DNASE1
IGFBP1
GFER
TMPRSS6
ADRB2
FTL
DGAT2
HSPA5
SPARC
GAL
NR0B2
EPRS1
IL21
KL
PTPN1
MAOA
FGB
CCK
GDF15
EIF2AK4
GPX1
PIK3CB
ITCH
MST1
NQO1
HMGB1
COL11A1
LTBP3
PPARD
TPM2
PIK3R3
THBS1
AKR1A1
APPL1
RARRES2
SDCCAG8
AGER
TMPO
EPAS1
IRF8
NR5A2
SLC25A22
CD163
SHC1
UQCRB
EIF4G1
SI
RPS6KB1
PLAUR
PALLD

TPM1
COX6A1
USF1
PEPD
CD59
CLRN1
ABCG1
NAT2
P4HB
GOLM1
MAPK9
CFLAR
NPC1L1
LGALS3
CD14
IL17RA
NDUFAB1
AREG
FDFT1
DPM1
TPM3
GABRG2
DICER1
PLIN2
NLRC4
MERTK
KEAP1
ATP5F1E
FOXF1
RPS27A
TM7SF2
BIRC3
ATF6
SLC25A12
SLC25A17
HDAC1
UCP3
FOXC2
GSTT1
PLTP
OGG1
ALAD
GABRA2
SULT2A1
DYSF
KLF6
POSTN
IL1RAPL2
SDC1
SOST
PHB
TSPO
IRF3
E2F1
IFNAR1
PRKCQ
EPHX1
COX4I2

NR1I3
TFEB
GOT2
MLN
CD68
IL33
RPS6KA3
LPIN1
SFRP4
UQCRFS1
SERPINA7
TRAF3IP2
AXL
ANXA2
CCR1
RPL5
GGT2
BID
IL18BP
NTS
ACACB
GPC4
MAP3K11
SLC39A14
CYP2B6
CCL20
SLC39A4
FTO
TYSND1
GFPT1
CXCR5
ANGPTL8
CYP4A11
IARS1
ABCC3
EIF2AK2
DNAH11
COX7B
BCL2L11
SAMM50
ATP2A2
PARVB
PHGDH
DARS1
EGR1
ENPP2
UFM1
GLP1R
NDUFA4
TGM2
IL6ST
TBP
TRIB1
CNR2
CANX
MYH14
LOXL2
AOC3

SAR1A
ASCL1
ACSM2B
RPS17
CEBPB
PTX3
OPRM1
OLR1
DCLK1
DBH
POR
GSK3A
HDAC9
OGT
EPHX2
STING1
IGF2BP2
SERPINB3
NCAN
FFAR4
SPTAN1
MATN3
SRD5A2
ITGA8
RPS3A
SLC25A1
PTPRD
ALDH9A1
PDXK
TXNRD2
MATR3
TXN
SLC1A3
IL16
SF3B1
TRAF2
CPB2
ATP1A1
TKT
MBOAT7
GCLC
PDGFA
SCAP
PKM
KHK
RUNX3
ERLIN1
IMMT
A2M
ERLIN2
FST
ALOX12
RB1CC1
IRGM
CYP4F2
UBD
CASP2
SIK1

COX6A2
FGL1
ITPR2
UGT1A6
HSPA1A
PTPRG
PRKAG1
LBP
VTN
RDH5
PIN1
P2RX7
XRCC5
TRIM21
CXCL11
CCL19
PRKAB2
UGT1A9
DMGDH
UGT1A7
GPNMB
CCR2
SNTA1
JAZF1
CYP4F3
CD82
CUL3
ACSL3
TNFRSF4
CDH4
LECT2
UGT1A8
PRPF8
MLX
ZNF365
FNDC5
FYN
CYLD
HYOU1
EEA1
NOX4
FOXA1
SERPINF1
PRDX1
EFTUD2
YEATS2
LPCAT3
COX7A2
CCT5
CLDN2
NNMT
YWHAE
COX6C
COPA
TRAPPC9
COMMD1
HNRNPK
SERPINA12

GOT1
DISC1
NAT1
TRAF1
XRCC6
PPP2CA
CES2
UQCR11
CYP2J2
UQCRC1
TNC
CARS1
TRAP1
RTN4
PRSS2
UGT1A4
UBE2O
YY1
JCAD
SIRT6
PLIN5
SNRNP200
MGAM
CDH5
YBX1
TP53BP1
NCOA3
RPL10
UGT1A3
STUB1
SOD3
UBE2D2
ROCK1
CACNA2D1
SLC34A2
AKR1B10
LGALS3BP
SERPINH1
SLC25A11
IFNL4
IL36A
ACE2
TXNRD1
HPS1
KHDRBS3
CFD
ITLN1
AMPH
AZGP1
POMP
SLC38A8
MMP11
PEG10
PRKCE
COX8C
GSS
LCN1
IL18RAP

PRKAG3
DROSHA
HS3ST1
RPL6
UGT1A10
PCLO
CYP8B1
LCP1
SIPA1L2
PPP3CA
SIRT4
APLN
HIGD1A
MAP3K7
PDGFC
RORA
TPM4
COX5B
PRKDC
SLC4A4
TFAP2B
UQCR10
UQCRH
NFIC
PZP
CFL1
YIPF1
COX7A1
HNRNPU
CXCL16
AQP9
HSPA6
SIRT5
COX7C
ARRDC3
PTPRU
COX7A2L
LEMD2
HTR1B
AHNAK
CLTC
SLC9A9
ALPI
HSD17B13
HDAC8
RIPK3
STEAP4
EEF1A1
MACROD2
SEMA5A
ADH5
MAPK12
CMKLR1
IL34
ADH1A
CCAR2
CCT4
ESM1

PNPLA7
ELK1
EHBP1L1
KAT5
PSMC2
GRK2
RPL3
CD5L
TNFRSF12A
COX7B2
CRACR2A
RPS15
COX6B2
NOS1AP
SLC25A47
PRDX5
ATF3
TRAF3
USP7
BIRC2
IL1R2
FARP1
GPN1
NRG3
CISD2
PCSK5
UQCRHL
SDC2
EGFL6
SLC25A3
INHBA
FAM166B
ESRRG
DDX60L
AQP7
HNMT
ADAMTS9
DGCR8
RPTOR
IGHG1
CAPSL
EDA
MAP3K8
TAGLN
STX17
TSHB
PSMD2
TP53BP2
UGDH
LAPTM4A
NMT2
SOCS2
PLPPR4
CX3CL1
AGAP1
RPL7
MOGAT2
YWHAG

GPLD1
CCRL2
CTBP2
UGT1A5
ARNT
CDK8
RPN1
GLO1
ST8SIA1
CREB5
YBX3
FAF2
MLXIP
AQP3
CYGB
YWHAQ
SLC46A3
FRK
CCN4
FAP
OSBPL5
SVIL
AATF
ANKK1
CWF19L1
CYP2A13
HCFC1
CYP2F1
CALD1
HSP90AB1
ZFP90
MYO6
ZNF512
PDIA2
FETUB
BCAT1
ADRB1
PCNX3
SLC3A2
TNR
AEBP1
UBQLN4
SEL1L3
TTF2
FOXK1
ZP4
C2orf16
TEX36
PPIB
RPL7A
SORCS3
SORCS2
ATAD3A
CAD
SLC30A6
NDRG2
VIL1
DDX1

NUCB2
CHST2
GPT2
MCM7
NFE2
APOF
CCNC
MYO1B
FAM171A1
PTBP1
RPL12
NFE2L1
BBC3
MYH10
FAM107B
HSPH1
IAH1
SFRP5
TRIB3
COLGALT1
RND3
ARPC1B
NCL
KCNK7
[illegible]ACROH2A2
DDX5
TMBIM1
HNRNPM
SDK1
SLC9A4
ATXN2L
PPP1CA
PFKFB3
POLR3H
RPL27
APPL2
KLHL6
PSG1
NRG4
GRM4
RPL30
SMIM20
HNRNPC
MYO18A
SENP3
CTSS
KRTAP5-5
CNOT4
KIF16B
RPS4X
FOXN3
SERPINA4
AGR3
GGT3P
MYL6B
POLR2D
WWTR1
PDE7B

DDX60
DDX50
SLC6A9
APLNR
RUVBL2
C1QTNF3
AP2B1
LINC02694
MCM3
GCN1
MRTFA
SIRT7
YWHAB
TUBA1C
ONECUT2
GATAD2A
SRSF6
PWWP3A
MCM5
ATP5F1B
RPL13
GSDMD
CCT3
H1-4
MCF2L2
RPS2
TMOD3
RPLP2
MYO1C
NUDT4
IGF2BP1
ACTR5
YWHAZ
CCT2
SLC25A10
RPS15A
RPS23
CILP2
RPS11
RESF1
C16orf95
IP6K3
XPO4
LINC00322
RPL10A
DNAJA1
SAT1
ZNF101
QARS1
TCP1
SLC30A9
GRIK3
SP4
RPL4
NUDT21
MYO1D
STRA8
PLPP7

EIF3L
ARHGAP8
IL36B
HNRNPF
SRSF1
RAB37
TRMT44
RPS9
BECN2
INSIG2
RPL8
GSTK1
RPS18
TLL1
ZNF267
G0S2
HNRNPH1
C1QTNF1
LRRFIP2
ANXA9
CRTC1
SNRPD2
RPL24
STMP1
DHX9
PSME2
RBFOX1
THNSL2
AP2A2
TUBA8
INPP4B
PABPC1
NLRP2
PSAPL1
SLC23A2
ACTR2
EIF4A3
ACTR3
PHF21B
ATP6AP1L
SLC23A1
SRSF7
SERPINB4
HNRNPA3
USP4
TET1
AP2A1
RPL23A
RUVBL1
IBTK
AUP1
ILF3
ARPC2
RRBP1
RTRAF
EIF4A1
SRSF4
SUGP1

ENHO
AHSA1
RNF114
CTPS1
SEC16A
RPS8
TRIM33
TMSB4X
SRRM1
DUSP26
EIF3E
DDX17
CCT8
CAMKK2
EDC4
EIF3B
HKDC1
CKAP4
HNRNPUL1
MAPK13
EIF3F
RBM14
UBE2D3
FARSA
HSD17B11
PDIA4
PGAM5
FAM3A
SLC25A48
PKNOX1
TMEM97
PABPC4
SF3B3
C1QTNF9
CAND1
MYBBP1A
USP48
TMC4
TXNL1
HNRNPL
EIF4A2
LAMP3
DHX15
SUB1
TMBIM6
ILF2
SRRM2
FAM3B
RTCB
OPRD1
GLT8D2
EBNA1BP2
LATS2
GGT5
HMCES
SMOC2
PHF2
SNRNP40

FAT1
DDX46
PHF5A
KDM4B
ARPC4
NMBR
UBXN1
SLC2A8
RGS4
FAIM
USP10
CLGN
LYPLAL1
SH3BP5
STK24
IGHG4
DUSP14
SEC16B
DNAJC10
RANGAP1
H2BC18
HSP90AA2P
COP1
H2BC11
RAB32
PNLIPRP2
RAMP3
TRIM47
TRPC4AP
ZNF300
SDF2L1
BMP8B
FNDC3B
DNAJC11
FUNDC2
HIF3A
RUBCNL
TXNDC5
PHLPP2
SAP18
MAS1
CTDNEP1
YIPF6
CARD6
SUCNR1
PRMT3
HERC4
CR1L
FAM174A
TM6SF1
GNMT
JAK2
PRKCA
KLB
FOLR2
GSTA1
IL4
LIF

PRKCD
VLDLR
CSF2
AHCY
ALDH1A1
ALDH1B1
B3GAT1
EIF2AK1
LAMA1
IL3
SERPINB2
PRF1
PRKACA
RAG2
RDX
IKBKG
STC2
ALDH4A1
ZGLP1
FBL
PIK3CG
SLC5A2
GGTLC3
CNBP
EHMT1
TP63
XPR1
GABPA
REN
GGTLC1
DECR1
TXNIP
ABCD1
FOXO1
GOLGA6A
APRT
MFAP1
NR3C2
RCBTB1
SELENOP
PPP1R3B
SLC27A5
FLII
CHPT1
CASP1
PRRT2
GPBAR1
DLAT
SMUG1
GIP
HPGDS
OR10A4
ANPEP
ARNTL
MT1B
ACLY
BCO1
PRKCB

BCL2A1
SAV1
SLC10A2
BNIP3
BCO2
BMS1
YAP1
CORIN
CCL27
MTMR11
ECD
BHLHE23
GPR119
CYP2D6
ATN1
MLKL
ELAVL2
ESRRA
ACSL4
FCGR2B
SIRT2
FOXO3
MLYCD
ALOX5
ALOX15
ATRNL1
GCGR
PDLIM3
GLUL
SLC13A5
SETD2
NR3C1
TBK1
PDIA3
HSPA1B
IGFALS
AR
MC4R
MUC1
CCN3
IL22
CRNKL1
HSD17B7
GDE1
LUC7L3
ELOVL2
AVP
NAT10
PTPA
MAPK1
MYDGF
PRTN3
PTGS1
MOK
S100A8
CEACAM1
SCP2
SELPLG

ABCG5
IL25
SLC2A2
NCF1
SLPI
SPTBN1
STAT5A
STAT5B
TEK
TRPV1
DHRS11
CALCR
ASRGL1
ORAI1
SOCS1
CBR1
RIPK1
SELENBP1
CD24
OCLN
DNM1L
PPIF
PLIN3
FSTL3
CAP1
STK25
NCOA2
ATG7
PRDX4
SORBS1
CXCR6
TNFSF13B
EBP
YME1L1
PLK4
HPSE
PGRMC1
NMU
TMED2
LILRB4
ADAMTS13
TREH
DUSP12
CHRM3
VSIG4
MGLL
TXNRD3
CIDEA
TWIST2
IFTAP
CYP2R1
CPT1C
SLC31A1
ADM
PPARGC1B
NADK2
CPN1
CREB1

ATF2
CREBBP
CRK
CRMP1
FGFBP3
CSF3
CSF3R
CTF1
PDIK1L
CTNNA1
HORMAD2
CTRL
CTSG
SLC2A12
CYP2D7
CYP19A1
CYP27B1
CYP51A1
DBP
DDOST
TIMM8A
DHCR7
DIO3
DMD
DPYSL3
SLC26A3
DSPP
DUSP9
S1PR1
EIF4E
LINC01554
ENO3
EPHB2
EPHB6
EPO
ESRRB
F2RL1
FABP5
ACSL1
PTK2B
UNC5B
FBN1
MARCHF8
FCGR3A
FCGR3B
FGF1
FGR
ZHX2
ALDOB
AKR1B1
MPRIP
MCF2L
ANGPTL2
BRD4
MMD
FOSB
FOSL2
METTL4

DAPK2
MKRN1
TMEFF2
ZBTB38
GALR1
RNF19A
TIPARP
OSBPL3
POLDIP2
TOR1AIP1
PRPF31
AMD1
FGF20
GDNF
NOX1
SIGLEC7
INTU
NAAA
IL37
GPC3
GLB1
RBMS3
GLI2
MAT2B
GLRX
GNA12
GNG13
ABO
GPI
ANGPT1
BTBD8
ANGPT2
GPR31
FFAR2
GPS2
MYLIP
CTNNA3
GSTM2
REPIN1
CERS2
H2AX
HADHB
HADH
HLA-A
HLA-C
HLA-DQA1
HLA-DQB2
HLA-DRB1
AOX1
NR4A1
FOXA2
BPIFA4P
HOXD13
AIRE
HRG
PRMT1
HSPA4
XIAP

HSPA8
HCAR2
MOGAT3
IGF2
IGFBP2
IGFBP4
IGFBP7
KLK3
IL2
CXCR2
IRF2BP2
INPP5D
INSIG1
ITGAM
ITGB1
ITIH4
JAK1
JUNB
JUND
ACAT1
ARG2
RTL1
C1QL3
FADS1
LNPEP
LTF
LUM
ARRB1
MAT1A
DNAJB9
MEFV
MET
MIF
CXCL9
MPI
MPST
MRC1
ASNS
NBN
NEU1
NHS
NOS3
NRF1
P2RX5
P4HA1
DUOX2
IL20
PAPPA
PCK1
ABHD5
MBTPS2
ISYNA1
ARID4B
SERPINB6
PIGR
PITX3
PLCG1
PLG

PMCH
DUOX1
TREM1
CCHCR1
EGLN1
CROT
SLC52A1
PPP1R3C
PIWIL2
NUDT11
SLC47A1
FBXW7
CHDH
TRERF1
ALLC
PAG1
ACOT13
ERBIN
MAPK3
MAP2K7
PRLR
LGALS14
PNO1
BAAT
SMURF1
PTGFRN
LRRC7
SEMA6A
PTPN6
PTPRC
PGAP6
BCHE
RARA
TRPV4
OPN1LW
RLN2
BRD2
SORT1
S100A4
CCL17
CXCL5
CIDEC
SELL
SETMAR
ABCG8
SRSF3
NSD1
BLVRA
GORASP1
ARV1
ST3GAL4
WNK1
BMP4
SLC6A2
SLC13A1
AACS
SIGLEC1
SNAI1

SORD
SPRR2A
SQLE
STAT6
SULT1E1
STK11
SYT1
ADAM17
TAZ
TCF7L2
TFAM
TFF3
TG
TGFB1I1
THRSP
TIMP3
NR2E1
TPO
DNLZ
SLC35A2
UGT2B4
UROD
UTRN
VEGFC
VIP
BEST1
ZBTB16
APOO
PLA2G7
CALCA
AIMP2
NLRX1
KCTD17
DHDDS
STAM
CPEB4
COASY
FOSL1
SPX
SLC7A5
SESN2
ANGPTL6
PLA2G6
CHCHD6
MAK16
FAM83A
DENR
RUNX2
RTCA
MBTPS1
TNFSF14
DLK1
TRIM24
HDAC3
MBD2
TBL1Y
MYOM2
TIMD4

HACD4
NOG
GLP2R
GRAP2
MPRSS11D
GGPS1
ATG5
CLOCK
CDH1
PLEKHG5
PARK7
KIF1B
PINK1
ECE1
RHCE
YARS1
ELOVL1
FAAH
FOXD3
DNAJC6
IL23R
TNNI3K
ABCA4
AGL
DBT
OTCH2NLC
ECM1
ZNF687
SLC27A3
GBA
MPZ
FMO2
NCF2
INAVA
TLR5
PSEN2
NBAS
MPV17
HTRA2
ZAP70
HOXD10
PDE11A
BMPR2
FARSB
SP110
GIGYF2
ATG16L1
SAG
AGXT
PDCD1
CAV3
CX3CR1
GBE1
POGLUT1
SEC61A1
RAB7A
ATP2C1
DZIP1L

GYG1
MME
GNB4
HTT
PROM1
UCHL1
PHOX2B
PKD2
PPM1K
SNCA
FAT4
TRIM2
ELOVL7
HEXB
PDE8B
APC
LEAP2
SAR1B
HARS1
LARS1
SH3TC2
FAT2
FABP6
DHX16
HLA-B
HLA-DPB1
PKHD1
ELOVL5
ELOVL4
BCKDHB
SEC63
FIG4
FABP7
ARG1
PRKN
HNRNPA2B1
PGAM2
CHCHD2
ABCB1
PON2
RINT1
IRF5
GATA4
NEFL
JPH1
GDAP1
PMP2
CDH17
