# Supplementary material for: Network Pharmacology-Based Exploration on the Intervention of Qinghao Biejia Decoction on the Inflammation-Carcinoma Transformation Process of Chronic Liver Disease via MAPK and PI3k/AKT Pathway
Source: Biomed Res Int. 2022 Oct 14;2022:9202128. doi: 10.1155/2022/9202128 (PMC9586778; doi:10.1155/2022/9202128)
Supplement: Supplementary Materials — Supplementary data associated with this article can be found in the appendix. Supplementary file 1 shows the compounds and their associated targets for QBD and Supplementary files 2-8 show the associated targets for CLD. [file 9202128.f1.zip › Supplement 5-HB target.pdf]

HB target（Results after deleting duplicate targets）
TNF
CD40LG
TP53
IL10
PTPRC
IFNG
HLA-B
FAS
RAG1
MYC
AKT1
CD19
CD40
RAG2
BTK
NFKB1
CD79A
APOB
CD81
IL6
FASLG
BCL2
JAK3
HLA-DRB1
EGFR
STAT1
IKBKB
IKBKG
INS
PIK3CA
HFE
CTLA4
CARD11
BCL6
GPT
SMPD1
CCND1
KRAS
ATM
SP110
IL7R
CASP8
LIPC
XIAP
PIK3R1
ALB
MYD88
ADA
HNF4A
ERCC6
NFKB2
CDKN2A
LDLR
F9
MS4A1
FOS
HLA-A

PTEN
JAK2
IL4
BAX
CD79B
IKZF1
CR2
LPL
STAT3
BRAF
ZAP70
PAX5
NFKBIA
HLA-DQB1
IFNA2
ABCB4
APC
F2
EGF
BLNK
APOE
NR1H4
IGHM
CHUK
PIK3CD
CTNNB1
CYBA
SH2D1A
BIRC3
FOXP3
IFNL3
TNFRSF13C
CCR5
AICDA
PPARG
BCR
TLR4
CC2D2A
HRAS
SERPINA1
BCL10
SERPINC1
CXCL8
IL2
LEP
TCF3
TGFB1
NCF2
TNFRSF1A
AFP
CDK4
NOTCH1
IL1B
GLB1
KRT18
ESR1
B2M
TFRC

PRKCD
PTPN11
MTOR
HNF1A
MAPK1
KIT
HAVCR2
ABCB1
GATA2
CD27
RSF1
CCL2
RELA
PLCG2
CYP2D6
MET
GGT1
RBCK1
HLA-C
NAGLU
SLC17A5
CDKN2B
CAV1
ABCB11
IGF2R
CYP3A4
IL10RB
MAPK8
LMNA
CD55
CASP3
AIRE
TLR2
TNFRSF13B
TERT
THPO
ABL1
SMAD4
PSAP
IL2RG
KRT8
CD3D
CIITA
MTTP
ERBB2
SCARB1
CASP10
CPT2
LAMTOR5
BRCA1
NPM1
CDH1
NPC1
F8
IL13
CLDN1
JUN
MAT1A

ERCC3
HADHA
IL2RB
IFNB1
VEGFA
EP300
SLC10A1
HAMP
NRAS
PMM2
AKT2
HMOX1
IL21
IL2RA
POU2AF1
GBA
DNM2
BCL11A
CDKN1A
CYBB
ALDOB
FADD
TNFSF13B
TLR3
NPC2
ACTB
HLA-DQA1
DDX3X
ALK
TYMP
JAK1
OTC
INSR
CEP290
BCL2L1
CP
NCF4
IL12RB1
G6PC
CYP2E1
MT-CYB
SLC25A13
MALT1
DCLRE1C
RAF1
LEPR
GRB2
MAP2K1
CXCR4
SRC
IFIH1
BIRC5
EIF2AK2
IFNAR2
SYK
ICOS
TCF4
CREB1

CDK1
FOXP1
CD247
ZMPSTE24
RASGRP1
NPHP3
MBL2
CYP1A2
PKHD1
MMP9
APOBEC3G
APOA1
BRCA2
ITK
CDKN3
FECH
GZMB
PDGFRA
ABCB7
CD4
ICAM1
CSF3
PNP
DGUOK
TM7SF2
FLT1
TSC2
CD22
NOTCH2
DDX58
HLA-G
CCR6
NR5A2
ARSB
CXCL12
FGFR1
MGMT
IRF3
F5
F13B
PCSK9
NCF1
CYP1A1
ASGR2
CD274
STK11
FAH
MCL1
TLR9
CRP
DOCK8
ARSA
PC
GLI3
ENG
MAPK14
MME
HGF

CASP9
CXCL10
NFE2L2
DNMT3B
LIPA
CD44
IGLL1
CD34
UGT1A1
LYN
IL18BP
CYCS
PTCH1
CFB
PRKCB
JAG1
REL
SLC2A1
CYP2C9
TMEM216
LIG4
CD70
HJV
MX1
MDM2
RFXANK
F3
ASS1
PNPLA3
TNFAIP3
CD28
POLG
CDK2
BSCL2
CD80
LRBA
TGFBR1
CYP1B1
AXIN1
NHEJ1
HADHB
ADIPOQ
PKD1
ADAR
AURKB
CFLAR
CD5
IL7
PSMA7
FCER2
PTGS2
ASGR1
MAPK3
TYK2
MVK
AGPAT2
CDKN1B
MAVS

IL21R
MTHFR
ALG8
TNFSF10
PRF1
RB1
GNG13
TSC1
RET
IL18
CCNA2
RIPK1
SLCO1B3
FLT3
CXCR5
OFD1
IGF1
IL1A
IFNAR1
MPL
TRAF6
CPD
ERCC1
CCL5
CAVIN1
TICAM1
ATP8B1
GUSB
HAVCR1
IFI27
AKR1D1
TNFRSF8
XPO1
HSP90AA1
CD38
ALG2
NR3C1
TMEM231
HEXA
AIP
AHCY
PTMA
RNF31
FGA
RFX5
MOCS2
TTC7A
LRRC8A
ETFA
SOCS3
TBK1
ICOSLG
GYS2
PRKDC
BLK
IL5
PAPOLG
HIF1A

HLA-DPB1
GM2A
GBE1
CBS
CTSB
CD86
BBS2
E2F1
ATP7B
CYP2B6
MAN2B1
HEXB
RARB
SLC35C1
PRKAG2
KRT19
IFNL4
NOS3
ANXA5
TFR2
PPARA
SEPSECS
WDR19
SFTPB
BAK1
IRF4
EDNRB
ACADVL
RFXAP
MPO
PIK3CG
ABCC2
IL17A
IL3
HBB
PHKG2
CRYAB
ITGB2
COG2
IKZF3
BAD
ASAH1
CD3E
NBN
HEPACAM
SMAD3
PDCD1
FGF2
EZH2
POMC
RAC1
CREBBP
DDB1
CFTR
RPE65
IRF7
SON
CSF2

PRDM1
TRAF3
ERCC2
CDK6
GLI1
CD8A
NOS2
PTK2B
GSK3B
AKT3
VWF
MRC1
STEAP3
HLF
SPARC
HELLS
RPGRIP1L
SAR1B
MMP2
NBAS
SIRT1
LMNB2
CYP2C19
EPO
IL15
TF
CAT
IRF5
MAGT1
VIM
TIMP1
IGHE
PHKA2
CD3G
GATA1
MAP2K2
GALNS
TRMU
SP1
IL4R
GNMT
MOGS
ERCC8
EPM2A
SELL
LEF1
IFT122
TMEM67
BBS12
DMD
MTUS1
WNT1
CLN6
TG
BMP6
TNFRSF1B
IRS1
PBX1

LTA
STAT5B
CXCR3
EVC
RSAD2
FBN1
CHIT1
SREBF1
RNASEH2B
GSTP1
TMEM237
INPP5E
HSD3B7
TRIM22
NHLRC1
CXCL13
PDGFRB
SERPINE1
RAD51
VAV1
MSH2
XBP1
EVC2
HNF1B
TAP2
IKBKE
ERCC4
RPS6KB1
LIPE
MPI
LCK
CLEC4M
SCARB2
GAPDH
CCND2
IDH2
STAT5A
BIRC2
FBP1
RTEL1
IL10RA
PKD2
FCGR2B
GSTM1
IL1R1
WRN
SGSH
TJP2
DPM3
ABCC1
ABCG2
HLA-DRA
TNFRSF10A
EDN1
GFM1
COL11A1
RNASEH2C
FGL2

LDHB
SERPINA3
SPP1
PRKCA
RELB
C4A
CFI
XRCC4
TLR7
IGF1R
SPI1
ITGAL
RXRA
ADA2
HSPD1
CYP3A5
IL6R
MMP1
CARMIL2
IGF2
LMBRD1
YARS2
SLC7A7
ANGPTL3
VCAM1
CCL3
FTCD
PTPN22
ALAD
ACE
CETP
PIK3CB
CCND3
MYB
KRT7
CARS2
PLAU
SOD2
FASN
CSTB
IL1RN
LOX
TCL1A
SOS1
BCL2L11
STX11
CCNB1
HLA-DPA1
UNG
ITGB1
IDS
UNC13D
PLCG1
KLRK1
TTR
VANGL2
ALOX5
TRMT6

TGFBR2
CCR7
PRKCSH
TPMT
TMEM138
STXBP2
ETS1
AGL
KITLG
TNFRSF10B
RAC2
GOLM1
GTF2H5
PTK2
PDGFRL
KDR
ANPEP
CD69
MMP14
SST
CCN2
LAT
CLDN6
UGCG
G6PD
MAP3K7
NQO1
SOCS1
ALDH2
RHO
PDGFB
CYP19A1
ALG6
IRF9
ALPP
APOC3
PSMB8
VDR
ITGA4
HDAC9
GATA3
TLR5
F7
NDUFS8
CLDN9
COG7
MICB
ACHE
HSPB1
HP
MAOA
NCAM1
THBD
ALG1
S100B
VPREB1
BCL3
SLC4A1

CD46
PGM3
IRAK4
MKS1
ATG5
HMGB1
RYR1
DZIP1L
RAB27A
CFH
SEC63
DACT1
P2RX7
CASP7
MEN1
TRAF2
NPPB
TNFSF12
C1S
NME1
IFT43
TGFA
WRAP53
IFIT1
IL12A
RETN
AR
SPIB
HSPA4
MKI67
PIK3R2
CDKN1C
WDR35
PML
TNFSF11
MBP
SHC1
ABCG5
DDOST
CD24
YY1AP1
LRP5
FYN
SLC35A1
AQP1
EGR1
HMGCR
TGFB2
APOBEC1
EIF2S1
PLAT
F10
GANAB
SMAD2
TAT
CSF1
PMS2
CHEK2

CHGA
CCKBR
MST1
CIDEC
SKIV2L
SLC35A2
PSMB9
TULP1
PEG10
MUC1
CALR
PROM1
CD36
NDUFS1
PLAUR
CR1
ABCC3
BST2
CCK
ACVRL1
HSPA5
IFITM3
AURKA
ARG1
CD33
CCL4
SELE
CD59
CCNE1
NTRK2
PCNA
SKP2
TEC
RAB7A
HDAC1
ATF4
PGR
BMP2
CDK9
ATP6V1B1
NPB
GLUL
ADRB2
CASP1
DDIT3
FOXO1
RHOA
TSPO
KLF6
CEACAM5
KDM4C
SDHB
GCH1
ELK1
DNM1
PRMT7
IRF2BP2
PNKD

PHKB
ELANE
M6PR
KMT2A
CLPX
DIABLO
U2AF1
PIGY
TP73
DKC1
IRS2
NEK8
CLU
ITGAM
MICA
TIMP2
CYP2A6
HM13
MAN1B1
LYST
CDC25A
CCL19
GHV4-38-2
NTRK1
HADH
TNFRSF17
BGLAP
DPAGT1
GLA
NOG
NR1H3
NOD2
C3
TIA1
SDC1
TAP1
TRIM25
NOTCH4
PTPN1
IL6ST
PTPN3
TPO
KHK
FUT1
CALCA
FN1
LCAT
FCGR3A
SPRTN
NEU1
DNAJB11
CD72
SLCO1B1
EPHX1
SLAMF1
DCK
SLC10A2
MIR17HG

CXCL9
MSN
GCK
GPC3
IGFBP3
TEK
SMAD7
EZR
PPARGC1A
ENO1
AGK
UROD
CD14
GHR
FCGR3B
RASSF1
HAX1
SQSTM1
IL12B
DLAT
CCDC85B
MRPS7
GRIN2B
ANGPT2
MARS1
APOH
PIK3R3
TGFB3
LGALS1
ABCA1
SP140
CCL20
DES
TLR1
MECOM
HGSNAT
HLA-DMA
TNFSF15
URGCP
ERBB3
SNAI1
DLL4
CPOX
NR0B2
F13A1
SPN
FABP1
PPIG
SMARCA4
MT-ND4
ARSH
IFNGR1
CXCL2
BACH2
ITGA5
LPA
STMN1
ATF6

GABBR1
APBB1
TKT
RFX1
PLK1
WT1
ADK
FLNB
TOP1
LMO2
HLA-DQA2
CYP7A1
PARP9
CYP7B1
SMARCB1
BSG
MVP
GFAP
PIGA
SSB
SURF1
MMACHC
MAP2K7
AGTR1
PRL
IL18R1
GAB1
LDHA
DEPDC5
PPOX
ACADS
WNT5A
SLC11A2
NLRP1
GJB1
DPYD
PON1
IDUA
POT1
CYP17A1
ESR2
TYR
IREB2
TRB
STAT6
ACADM
GCG
GSTT1
SELP
COG4
E2F2
RBP4
VTN
POLG2
NAMPT
ETV6
MMAB
PRDM16

PHB
TNFSF13
TTC21B
HPRT1
AREG
IRF1
GSR
SF3B2
PIK3C2A
EXTL3
PECAM1
RRM2
PXN
DAG1
IL9
GPR35
TYMS
SERPINB3
P4HB
GPS2
GH1
KEL
NFKBIB
PRDM2
ONECUT1
CLN3
MMP3
HSP90B1
CEBPA
ANGPT1
SERPINB8
CXCR1
CBLB
MMP7
GAST
EPCAM
ITM2B
CCL11
VIPR1
NPHP1
IFT80
PLG
ZEB1
SAMD9L
ACADL
IDO1
PLEK
CSF3R
IRAK1
MTR
ENPP4
HMBS
VEGFC
CPQ
PLA2G6
RUNX1
ABCC4
MAPK10

DPP4
APOBEC3F
CTSD
TOP2A
BECN1
FOXM1
SLPI
NR1H2
CDH2
PPIA
ENTPD1
SLC22A2
BMI1
ZNF423
CD58
BMPR1A
GALC
ITGAX
IGFBP1
CBL
NFKBIL1
SLC40A1
ELN
ATRX
CPB2
YAP1
ERBB4
FURIN
CDC25C
PTHLH
RPS27A
LMNB1
MIF
WAS
ANXA2
DHFR
COG6
APOA4
DNAH8
TLR8
SLC6A4
GBA2
ITPA
ACP5
ABCA4
GLS
PTPA
DDB2
UGT1A7
MAP3K5
GADD45B
CXCL1
ACD
TRAF1
B4GALT1
MLXIPL
ZBTB24
MYH7

TNFRSF11B
CENPB
POMT1
SLC3A1
PIP5K1C
BHMT
FGFR3
URI1
CREB3L3
BRD4
TTC37
PTPN6
MT-ND1
APOC2
ISG15
XRCC5
TCOF1
ALAS2
TCTN2
POLR2L
CFP
CPT1A
CD84
AGXT
SREBF2
PRKN
MDK
CHEK1
ENPP2
FSCN1
SLC46A1
AGER
NR4A1
CD1A
SUOX
TWIST1
EDNRA
STING1
FOXA2
PPT1
REN
PPIB
ITGB3
DPM1
NPR2
RECK
NPHP4
SERPINB9
LIN28B
PHF20
FUZ
ANO5
CHKA
DKK1
CARD10
RPS26
CD9
VIPAS39

GDF2
STAT2
BID
SYP
LGALS3
FGFR4
ACP1
TRMT10C
SFTPC
VPS33B
EPAS1
IFITM1
TNFRSF11A
CEBPB
POU5F1
PKM
THBS1
FCGRT
TCF7L2
PF4
PRKD1
HK2
GRHPR
PTX3
SLC22A5
WFS1
NAT2
TIMP3
COL8A2
SH3KBP1
DGAT1
LCN2
CRH
SBDS
SLAMF7
EPHA2
WNT3A
CTSA
CREB3
CD209
TNFRSF4
LIPT1
IQCB1
C2
SNAI2
KEAP1
PIM2
AK2
ARID1A
BLOC1S1
UCHL1
HPX
B9D1
TPP1
MSH6
CLPB
CYP2C8
AHSG

MAP3K14
CTTN
HMGCL
POMGNT1
INVS
EIF2AK3
PRKAA1
IL11
AHI1
E2F3
PIGR
TLR10
PNLIP
UBD
APOBEC3B
NPPA
SCYL1
HSPA1A
DNAJC5
SLAMF6
SHH
RUNX3
ACTG1
TLR6
FGB
HCK
NPPC
TNPO3
CRAT
BAAT
COMT
GSTO1
SLC7A8
PIGW
BBS1
BDNF
RPGRIP1
MT-ND6
NUS1
CRYAA
SEC61A1
VPREB3
SCT
PALB2
FOXO3
HPSE
TNFRSF6B
ATR
RRM2B
ATP6AP1
MAP3K1
ADAMTS13
POLR2A
NDUFAF1
CACNA1C
B9D2
ARL13B
CCNT1

MMEL1
LTF
BTNL2
DICER1
COPS5
GLIS3
SMARCA2
MGAT5
GHRL
ALG12
HOXA13
SHBG
ITGAE
MTA1
ADARB1
JUNB
FCGR2A
CCL17
GPX1
PTH
PRODH
CEACAM1
TM6SF2
RPL5
RTN4
NR1I2
TIFA
AKR1B10
GSTM3
EHMT2
KIR3DL1
MAD2L1
SAFB
VHL
GSTA1
FKTN
DVL1
KRT1
HLA-DQB2
LAMP1
LOXL2
FKRP
APOA5
RPS28
CCL21
STT3B
SSTR2
APEX1
CUBN
CA5A
FZD7
CD248
SPTAN1
XPA
ARID2
CD82
NFATC1
ADIPOR2

NR1I3
FCN2
KIF1B
UBE2L3
DLC1
TRIM31
HLA-DOB
CCHCR1
TCF19
SH2B3
CDSN
HBS1L
CHRNE
RBM45
YDJC
CYP21A2
ERG
FBL
GABRG3
GCKR
GP1BA
HSPG2
ITPR3
SFTA2
GGNBP1
PAEP
IL22
CDK14
RBFOX1
RING1
BRD2
ATXN2
GPSM3
ERVK-6
DDR1
HSD17B8
BRAP
SMC5
OPN1SW
STAT4
SLCO6A1
GSTK1
KRT20
HPGDS
SLC26A5
ABO
ISG20
MECP2
ENOSF1
PLAAT4
RECQL4
ABCB6
CHP1
DNA2
IL27
IL37
LGALS3BP
IL23A

PIN1
SLC12A9
IL33
CAP1
SORBS1
PDLIM5
TMED2
TPPP
IL17F
NRSN1
CBLL2
VWCE
ARC
GEM
IFNL1
KIR2DL3
LNPEP
SERPINB6
IL17D
PRNP
ZC3HAV1
SRY
PRDX2
C4BPA
XRCC1
CARD14
MUL1
KMT2D
GPT2
HACD4
KMT2B
ERVK-11
NXF1
SUB1
NLRP3
IL34
CST3
IL23R
BTLA
AGT
SEC14L2
AGO2
GSTM2
ERVW-1
HLA-DOA
HLA-E
HSF4
IGFBP7
ITIH4
KLRC1
LGALS9
MOV10
OCA2
PELI1
SALL4
BCHE
S100A9
SERPINB4

ROBO3
SMYD3
SPINK1
YY1
USO1
XPR1
MTDH
PRDM6
AIM2
CDA
ERVK-9
EBI3
PSME3
KLRG1
CDKN2C
PRMT5
ERVK-19
BATF
MRPL28
GNLY
AHSA1
WDHD1
CISH
CTHRC1
ADH7
CRK
IFNLR1
DDX5
DHX15
DMBT1
AGRP
SLC26A3
ATN1
EIF5
APOBEC3A
ETS2
RBM24
SIRT2
FLNA
SUZ12
RNF19A
POLDIP2
FGF21
GNA12
MIR7-3HG
DLL1
SETD2
NXT1
HOXD13
HPN
HSPA8
DNAJB1
IFI16
INSRR
KIR2DS1
KIR3DL2
KIR3DS1
LAG3

LASP1
OAS1
OGG1
PLCE1
ISYNA1
PLA2G2A
LAPTM4B
PSMB6
SEMA6A
REG1A
S100A4
SATB1
CCL22
SET
SFRP1
SFRP5
SIAH1
SLC25A1
SMARCA1
SMARCE1
SRPK1
TRIM21
GGTLC3
POTEF
GGT2
DNLZ
TNFSF4
XRCC3
ZMYM2
MANF
TNFAIP8L2
AIMP2
TREML2
CALCR
BEX2
TNFSF9
HDAC3
PER2
ARTN
CD1D
IL32
GRAP2
PPIP5K1
SETD1A
FARP2
TANK
SRA1
KIR2DS2
CHAF1A
TRAP1
BCAP31
CDH17
ZNF197
MYMX
TRIM13
EIF1
CDK2AP2
PAK4

TNIP1
AKR1A1
CDO1
NDRG1
CDX1
KAT5
ZNRD2
UPK3B
HTATIP2
CENPA
KHDRBS1
DCTN6
EBP
DLL3
CFL1
KDM5B
PPP1R13L
SMR3B
MRPS30
JTB
UTS2
SPIN1
APOBEC2
PRSS21
PNRC1
ASCC3
SLC38A3
GTF2A1L
STON1
TENT4A
DNAJB4
RPP14
PTP4A3
CHI3L1
DCTN3
USP18
CDCA5
VSIG4
PHB2
PDAP1
TUSC2
KCTD12
FCRL1
UHRF2
MORN4
CYP2R1
CLTC
TPPP2
PLIN2
CNR1
SCLT1
PPARGC1B
COX8A
CRHBP
CRHR2
DOCK11
TRIM69
CRYZ

SLC38A6
VCAN
PDIK1L
RNF187
GPBAR1
CXADR
CYP27B1
AP1G1
APOBEC3H
DHX9
CLEC4C
PTCRA
AFM
DIO3
DNASE1
DYNC1H1
DPT
DTX1
EGR2
AHR
EGR3
EIF4E
EIF4EBP1
EIF4G2
ELAVL2
ELAVL1
ELK4
EPHA1
EPHA3
EPHB2
EPHB6
ERN1
EXT1
ABCD1
ALDH1A1
F11
FCER1A
FDX1
FEN1
ALDH3A2
FGF4
FHL1
ZHX2
MMRN1
RAB18
SPEN
TBC1D9
ZCCHC14
JMJD6
FLT4
USP22
PPP1R13B
SIRT3
TARDBP
FOSB
LILRA4
NUP62
LY96

KIF4A
FTL
FYB1
CADM2
TAC4
GABPA
GCA
BAMBI
AMBP
SAMHD1
CNTNAP2
GC
GCHFR
AGO1
HS6ST3
GFER
GFPT1
CBLIF
ABL2
NSG1
CACYBP
CIDEB
UBE2S
POLL
APOBEC3C
GPI
IFNL2
UTS2R
LYPD5
IGHV1-3
SCARA5
LAMTOR2
GRN
KLF15
GRIN2A
PDIA3
GTF2B
GTF2H1
GPSM2
HOOK2
GYPA
GYPB
IL19
GYPE
TBX21
ANXA1
H2AX
HBE1
SERPIND1
HDAC2
HINT1
HIVEP2
ACACA
ANXA13
HLA-F
MR1
HMMR
HOXA10

APCS
HRC
PRMT1
HSPA1B
APOA2
RAB7B
IFNA5
IFNA6
APOC4
IFNGR2
IGHA1
IGHG3
APRT
TICAM2
IL16
TNFRSF9
INPPL1
ITGA6
ITGA1
ABCC6
ITGAV
EIF6
IVL
JUND
KCNA5
KIF2A
KIR2DL2
KLK2
KLRB1
KLRD1
KRT14
KRT35
VHLL
LGALS4
ST20
SH2D5
LTB
LTBR
LTK
LY75
ARR3
ARRB1
MAFG
MAGEA3
MAT2A
MCC
MFAP1
MGAT3
MIA2
FOXO4
MMP8
MNAT1
ASL
MSR1
MSRA
MTRR
ATF3
RERE

NEDD4
ATP1B3
NRF1
NUCB1
NUP88
OAS3
SIX6
ORM1
PEBP1
PRDX1
PAK1
IL20
REG3A
ASAP1
TMED5
TMED7
DCTN4
PCYT1A
TNFRSF12A
ATRAID
PDE4A
POLK
SIRT7
TDP2
GDE1
SF3B6
CD244
ATP8A2
PGF
SLC25A3
SERPINE2
ATP6V1E1
PIN4
PLSCR1
NANS
ANLN
CPVL
POU2F1
UGT1A6
MARCHF5
TET2
KCTD9
SWT1
CASZ1
PINX1
ACP3
LAMTOR1
PPP1R1A
MED9
GPATCH2
PPP2CA
MSL2
INTS10
VPS53
FBXW7
SAGE1
SRGN
MAML3

GALNT10
UBE2Q1
PRKAA2
PRKAB1
TENM3
PRKAR1A
RCOR3
PAG1
USE1
APOM
MYDGF
PROX1
KLK10
PSMA1
PSMA3
EXOSC5
MEPE
PSMD8
PSMD9
PSMD10
MCOLN1
RAB22A
KIDINS220
PCDH10
PTN
SFMBT2
PTPN12
PTPRH
RAB5A
RAB5B
RAD52
RAP1A
BCKDHB
OPN1LW
RHEB
RNASE1
RNASE3
HPSE2
RORA
RPS15A
RPS19
CLIP1
S100A6
SALL1
TSPAN31
CCL8
CCL13
CCL14
SDC2
GOLPH3
SMOC1
XYLT2
PJA1
SFRP2
SRSF3
TENT4B
SRSF4
XPO4

CLEC7A
GORASP1
SHMT2
SLA
WNK1
MPPE1
SLC5A5
SLC6A2
SLC6A8
SLC12A3
SLC22A1
SMARCC1
SIGLEC1
SNCA
SOAT1
UAP1
SPG7
SPINT1
SRPK2
SSRP1
ST14
SUV39H1
SYT1
BTD
ADAM17
TBX1
TBP
TDG
TMBIM6
TERF1
NR2F1
TFPI
TGFBI
THRA
TIAM1
TNFAIP1
TNNI3
TP53BP2
TPP2
TUFM
ACOD1
UBE2I
UCP1
UQCRH
VBP1
VCP
CLIP2
ZFP36
CA12
DNALI1
CENPM
AHNAK
FTO
MBOAT7
VTCN1
STEAP4
LIN28A
MAFK

AGMAT
HDAC11
CALD1
STAM
PDCD1LG2
CD276
FOSL1
CUL5
MAP1LC3B
SLC14A2
TRIM7
MED25
NCOA3
CAPN2
TKTL1
RAB33B
SOX7
SESN2
HDAC10
SPARCL1
ING5
SARNP
CAVIN2
CASR
DGAT2
SMARCA5
HDGFL2
PYROXD2
TRIM52
RIOX2
MFSD2A
ZNF382
ATOH8
TRIM5
LGR5
PLPP1
NCOA1
CBFB
DYNLL1
EIF3A
EIF3H
TNFSF14
NRP1
SOCS2
KAT2B
CDK5R1
ASAP2
SPHK1
CCNA1
AP1M1
P4HA2
SELENBP1
NAT1
CCNG1
PAPSS1
PRMT9
ZNF479
RGN

NMI
CCNE2
PDCD5
EXO1
MYOM2
IL1RL1
DCLK1
MTA2
MSC
AIMP1
CD163
TCEAL1
SNAP29
FOXP2
TMPRSS11D
SFXN1
NCR1
MAP4K4
HOMER1
GAL3ST1
GDF15
EEF1E1
GOSR1
PTGES
CXCL14
CHD1L
CLOCK
RBM39
CREB5
RUBCN
RAPGEF5
MTSS1
SPATA2
ZEB2
SETDB1
SEC24D
CDC6
MFN2
HS3ST3B1
USP15
CDC42
CLCNKB
C1QB
ELOA
ATP6V0B
ZYG11B
ATP5MC2
SEC22B
FAM72B
BCL9
NOTCH2NLB
H2AC21
RHBG
CD48
FCRLA
FCRLB
SEC16B
FAM20B

APOBEC4
RNPEP
PPP2R5A
LEFTY1
ITPKB
H2BU1
ABCB10
RHOB
EFR3B
PPP3R1
INO80B
POLR1B
RALB
MZT2B
PLEKHB2
CRYGB
DNAJB2
FAM124B
RNPEPL1
TRNT1
GMPPB
CYB561D2
IL17RB
NFKBIZ
PHLDB2
DNAJB8
PPP2R3A
SPTSSB
FETUB
PPP2R2C
APBB2
RASL11B
POLR2B
DNAJB14
BANK1
SEC24B
ANK2
GATB
KLKB1
TAS2R1
RETREG1
NIPBL
BDP1
RGMB
TIFAB
FAM13B
MZB1
APBB3
PCDHGB1
PCDHGB2
PCDHGB3
PCDHGB4
PCDHGB5
PCDHGB6
PCDHGB7
PPP2R2B
EBF1
FAM193B

MGAT4B
HUS1B
FAM50B
PBX2
PPP2R5D
HSP90AB1
NFKBIE
NMBR
FAM120B
EIF3B
ABCB5
HOXA9
PURB
BCL7B
DNAJB9
ABCB8
BLACE
DNAJB6
EBF2
NPBWR1
FAM110B
ELOC
RMDN1
RAD54B
VPS13B
LRATD2
CYRIB
NFIB
DNAJB5
UNC13B
RORB
GABBR2
PPP3R2
RAD23B
PBX3
NIBAN2
DIPK1B
NELFB
UNC5B
DNAJB12
BTAF1
GOLGA7B
SEC31B
FAM53B
ABRAXAS2
EBF3
PPP2R2D
DHODH
