# Supplementary material for: Network Pharmacology-Based Exploration on the Intervention of Qinghao Biejia Decoction on the Inflammation-Carcinoma Transformation Process of Chronic Liver Disease via MAPK and PI3k/AKT Pathway
Source: Biomed Res Int. 2022 Oct 14;2022:9202128. doi: 10.1155/2022/9202128 (PMC9586778; doi:10.1155/2022/9202128)
Supplement: Supplementary Materials — Supplementary data associated with this article can be found in the appendix. Supplementary file 1 shows the compounds and their associated targets for QBD and Supplementary files 2-8 show the associated targets for CLD. [file 9202128.f1.zip › Supplement 7-HF target.pdf]

HF target（Results after deleting duplicate targets）
CFTR
TNF
TGFB1
NPHP3
IL10
IFNG
TERT
RTEL1
TMEM67
HFE
RPGRIP1L
CC2D2A
NPHP1
PKHD1
SERPINA1
NPHP4
TP53
CEP290
CAV1
IL6
CCN2
CD40LG
HLA-DRB1
ALB
PAPOLG
GPT
FASLG
NR1H4
MKS1
CTNNB1
TMEM216
TTC21B
SP110
TCTN2
FAS
CXCL8
EGF
ABCB4
B9D1
NEK8
SERPINE1
AHI1
SMAD4
ACE
WDR19
MBL2
OFD1
IQCB1
CCR6
GBE1
TMEM231
B9D2
INVS
KRT18
IL1B
BBS2
PTPRC

ABCB11
HMOX1
IL13
MUC1
IFT140
PKD1
HAMP
F2
IFNL3
IFT80
BBS1
ARL13B
AFP
STAT1
HNF1A
HNF4A
SLC25A13
SDCCAG8
TNFRSF1A
GGT1
PPARG
KIF7
IFT122
SFTPA2
CTLA4
CSPP1
KIAA0586
PIK3CA
CLDN1
HJV
TLR4
GYS2
STAT3
CP
SLC17A5
BBS4
LOX
SRC
CD81
ATP8B1
IL4
INS
TINF2
NHP2
ANKS6
NOP10
JAG1
INPP5E
WRAP53
HLA-A
HGF
MMP9
SERPINC1
CCR5
G6PC
WDR35
IFNA2
TCTN1

CPT2
HLA-DQB1
LMNA
FOXP3
TMEM237
ABCA3
LDLR
IFT172
SFTPA1
ALMS1
SLC40A1
TMEM138
CXCL10
TIMP1
TCTN3
ENG
TRAF3IP1
MAT1A
TLR2
MET
IFT43
CDKN3
CCL2
TLR3
HNF1B
STN1
PRTN3
APOE
VEGFA
KRAS
CEP164
DKC1
CRP
SMPD1
C2CD3
TYMP
FAH
SFTPB
JAK2
MAPK8
HADHA
PKD2
KRT8
IL17A
SPINK1
DYNC2H1
IKBKG
MMP2
CYP7A1
THPO
HLA-B
TGFBR1
CPLANE1
POLG
AKT1
BMP6
TFR2
FECH

APOB
TJP2
BBS7
NOTCH1
ATP11A
DGUOK
ACTC1
ABCB7
LPL
BBS5
PNPLA3
SPP1
PDGFRB
MTTP
GLIS2
ELANE
APC
CEP120
SMAD3
FGF2
IL18
IFIH1
UROD
AIRE
BBS10
CD4
MMP1
IFT52
HLA-DQA1
IRF5
IL12RB1
NFKB1
SFTPC
DCDC2
DYNC2I1
HRAS
ALOX5
PDGFRA
IFT27
ASS1
EVC
BBS12
CDH1
ELN
EDN1
TSC2
RBCK1
ASL
GLIS3
ATP7B
SLC10A1
EVC2
CCL5
CALR
PHKG2
HSD3B7
POLG2
MUC5B

OTC
SFTPD
LEP
TRPV4
TMEM107
FOS
NEK1
EGFR
INSR
IGF2R
SLC11A2
ADRB2
XIAP
TLR9
CDKN2A
HADHB
ALAS2
CD34
NBAS
DYNC2I2
RPGRIP1
IL5
PSMB8
SEC63
CYP2E1
IL1A
TCF4
REN
ALG8
MPO
UMOD
ABCB1
PRSS1
BBS9
PRKAG2
IL2
ACADVL
HIF1A
CEP83
FCGR2A
NPM1
HLA-DPB1
CCND1
PRKCSH
MAPK1
NFE2L2
MPV17
HADH
MPI
FADD
DYNC2LI1
CEP41
AKR1D1
SMAD7
JUN
BBIP1
BSCL2
AGL

ARL3
SLCO1B1
SMAD2
VCP
CXCL12
CASP8
HAVCR2
HAVCR1
KRT19
CXCR3
SEPSECS
IFT74
IGF1
FN1
CXCR4
TGFB2
RNF31
IL1RN
SLCO1B3
PMM2
COL11A1
JAK1
ADIPOQ
MPL
ABCD3
CCL3
PTCH1
IL1R1
MUC5AC
STEAP3
AGPAT2
PSMA7
TYR
RSF1
SOCS3
ABCG5
CD79A
HMGB1
ABCC2
HP
IFNB1
TGFB3
CXCR1
NHLRC1
ZCCHC8
MMP7
TM7SF2
THBD
BRAF
DNAJC5
EPO
TF
FBN1
RRM2B
PSMB4
TUBB3
EPM2A
AP3B1

CCL11
ZNF423
AXIN1
FARSB
DZIP1L
SKIV2L
GANAB
CD8A
IFNAR2
DLL4
SCYL1
WDPCP
PHKA2
TTC37
GPD1
MX1
FGFR1
MT-ATP6
CSF3
CYP3A4
GPR35
EIF2AK3
CSF2
PPARA
MST1
CCL4
ADAR
CXCL9
CASP3
NPC1
HPS1
ENPP2
PDGFB
CCL17
CHUK
PTPN11
DDX58
PTGS2
TSC1
ICAM1
CYP7B1
IL12A
LIPA
CEP104
ALG9
SPARC
BRCA1
IDH2
TWNK
ATM
ICOSLG
MARS1
SNAI1
CD36
F3
AGT
SLC25A15
ACTB

AKT2
ITGAM
CPS1
LYST
UGT1A1
AHCY
PTPN22
PYGL
APOA1
NRAS
ARHGAP31
MKKS
FLT1
ACOX1
FUZ
CHI3L1
CIDEC
MYC
ALDOB
CYP1A2
XPNPEP3
IRF3
KRT7
ABCC1
HSPA5
CAVIN1
IL2RA
CD27
GRB2
SP140
VCAM1
NFKBIA
VIM
ATF6
PLG
ANXA5
LOXL2
NEK9
EPHX1
CAT
AGTR1
IFT88
NOS2
GNMT
MT-CYB
GAPDH
SLPI
IFI27
CEP55
MAVS
CD19
COL8A2
GATA1
CXCL1
VEGFC
ACVRL1
SLC2A1
TNPO3

PCSK9
ACAD9
LARS1
ETFA
SCNN1A
PIK3R1
KIF3A
CYP1A1
BICC1
KATNIP
CCL18
GSN
SREBF1
ENPP4
CCR7
MTOR
IL9
HLA-DPA1
SLC7A7
CYCS
PDCD1
AGER
LGALS3
ALG2
GOLM1
GREM1
LTF
RINT1
TLR5
ALAD
EZR
SIRT1
NOS3
DOCK6
SST
ACP5
FTL
IFNAR1
PLIN1
SERPINA3
CHIT1
NOTCH2
TGFA
ACD
GSTM1
BMPR2
PGM1
TTR
STK11
PTRH2
RBPJ
POSTN
BCS1L
DOLK
RAB27A
LIPE
IFNL4
PDGFRL

RAF1
HBB
TNFAIP3
MYO5B
MMP12
MTUS1
TRMU
CDK4
ARG1
FBP1
NDUFS1
NOX4
BTK
IKBKB
FAM111B
TNFRSF13B
NR0B2
ATP7A
PAH
NHLRC2
SAR1B
LAMP2
PDGFA
SLC10A2
RETN
CDKN1A
TALDO1
AGTR2
ELMOD2
RNASE3
MT-ND4
PTEN
CCL22
CXCR2
DSP
TOLLIP
MDM2
SLC9A3R1
TNFSF15
S100A9
F5
RPL5
TSLP
FASN
SH2D1A
GNG13
POU2AF1
MMEL1
POMC
IRS1
BPI
LEPR
SOD2
GLA
CXCL5
UTP4
CCR4
CR1

MAPK3
BTNL2
TFRC
VDR
DMBT1
TNFRSF1B
CYP2D6
CYP2C9
NPC2
IFT20
ATP12A
CANX
RPGR
TNFSF10
DDIT3
RPE65
CREB1
ASPH
TM6SF2
KCNN4
IFIT1
RYR1
C8orf37
AGK
DNAJB11
CTSG
DPM3
NR1I2
MAPK14
SCARB1
NR5A2
GSTP1
ALPP
HLA-C
SPIB
GBA
GPC1
CD80
XBP1
ACOX2
GLRX5
NLRC4
PEX1
NR1I3
SCGB1A1
CLDN10
TMEM199
ADK
KDR
DLD
TBK1
ABCG8
RAG2
S100A4
PPARGC1A
ASGR2
ETFB
CD44

BGLAP
MUC6
MUC4
GZMB
HSPA4
IGFBP3
TPMT
TMEM17
MT-ND1
TPO
EIF2AK2
BCL2L1
MMP3
PIK3C2A
G6PD
ALDH2
F13A1
HSPA8
VIPAS39
VIP
NR1H3
SURF1
BAX
UBE2L3
MRPS7
DLAT
SERAC1
ARL6
IL10RB
SDHB
BCL2
SAMHD1
STAT4
HSP90AA1
ATP6AP1
TEK
DMD
SELP
CCDC115
IL6R
ZMPSTE24
HSPD1
DPP9
MRC1
GRP
ABCC3
TGFBR2
JAK3
SLC35A2
RBP4
VDAC1
CD274
WRN
SHH
DES
ENTPD1
SLC30A10
GDF2

HSPG2
VPS33B
PLAT
CLCN3
KIT
TIMP2
ATG5
FABP1
PRDM10
TXNDC15
MLXIPL
IL18BP
ENO1
IREB2
NCF2
ALG6
KIF1C
MIF
MCL1
CEACAM5
PRF1
YY1AP1
SPRTN
PLAU
SELE
ALG1
IGF2
RELA
SH2B3
TNNT2
LZTFL1
SCO2
LIG4
CPOX
CALCA
SOX9
GAA
ABHD5
LTA
APEX1
HSD17B4
EZH2
TNFRSF6B
RPS27A
GCK
COG6
ATF4
HLA-G
TTC8
UBC
IGHE
VWF
TTN
YAP1
MT-ND6
EHHADH
ATP4A
C1S

CCDC28B
E2F1
ICOS
STING1
ANGPT2
EIF2S1
SLC37A4
SCARB2
BIRC5
NOTCH4
COG7
ASAH1
PIBF1
ERBB2
IL15
PCK1
SOCS1
FAN1
SRD5A3
MTHFR
NDUFS8
CAMP
AP1S1
CEP97
RARB
EPCAM
AHSG
IL3
BMP2
SGK1
ADIPOR2
SP1
SBDS
ESR1
VCL
HSPB1
EOGT
CD28
CASR
ASGR1
PRKD1
COG4
TTC30B
PDE6D
IL7R
RXRA
PHF20
GC
NR3C1
CFLAR
CD40
NARS2
GPC3
ARMC9
TLR7
NUP107
IL12B
FGF21

CEP19
WT1
PMPCA
RAC1
PSMA3
CETP
CDK1
PRKCD
KIF17
PKHD1L1
PAX2
CTC1
GLUL
PIK3CG
MMP14
SERPINH1
SLC4A1
IFT46
APOC3
SKP2
HEPACAM
DPP4
YARS2
IFT22
NLRP3
GSK3B
PTK2
IGF1R
TNFRSF10A
SH3BP2
PRL
IL21R
CXCL2
CPT1A
TET2
DEPDC5
GGTLC3
PLAUR
PNKD
SYCN
GAST
AQP2
GALC
SHARPIN
GUSB
IL2RB
TK2
APOH
ADCY10
FLNC
TOP1
PSMD1
IFT57
KIAA0753
IRAK4
NPHS1
IARS1
SLC30A7

MAP3K5
EGR1
VIPR1
IRS2
FGA
PNLIP
GUCY2C
COG8
HMBS
CDC25A
TTC7A
FGF19
KRT13
PROM1
GLI3
DNMT3B
DPM2
CST3
TARS2
BRCA2
PEX5
NOD2
TRHR
MYBPC3
DCN
PIGM
REG3A
MORN1
ADA
MAP2K1
STAT5B
TRAPPC11
CD86
SQSTM1
RPL11
CSF1
BLOC1S1
RNASEH2C
MCM4
THBS1
LMF1
NAGS
TIMP3
DNAH8
TRIM21
PSMB5
COG5
ETS1
MOGS
EPAS1
ALG11
EDNRA
HPX
SLC9A3
KLRK1
AR
CCP110
PSMD14

CC2D2B
FOXM1
IL33
CASP9
CEP89
PTX3
GGT2
ITGB1
SREBF2
RNASEH2A
RHOA
TWIST1
ABCC4
PRKCA
KITLG
CYP2A6
LNX1
RIPK1
CLEC7A
SCLT1
VANGL2
WNT5A
DHDDS
VMA21
OTULIN
ADIPOR1
TNFSF13B
COG2
HMGCR
MYO5A
CDKN1B
APPL1
MT-CO3
BECN1
NR3C2
GSTT1
PSMD2
NPPB
TLR1
TNFRSF11B
CDK2
NOTCH3
TNFRSF10B
SMARCA4
TRIM25
MRPS22
MYH7
PTMA
ANXA2
ABCD1
PTPN3
CD209
CREBBP
SERPINB3
DPM1
HSP90B1
HDAC1
BSG

PIFO
DNAJC21
RAB3IP
CNTNAP2
CD63
ANGPT1
MRPL44
RAB8A
IL13RA2
SUFU
ZEB1
TUSC3
F8
PI4KA
CASP7
PEX13
GALNT9
GZMM
ZNF648
CLDN6
AREG
RRM2
MECOM
NUP133
COX10
PEX19
F2R
ERN1
BMP15
PRKAA1
RASSF1
RPS6KB1
TRIM32
SEC61B
RNASEH2B
MLN
ACO1
SLC6A4
GLI1
MAGEA4
GAMT
IL4R
CENPB
HPS4
CIITA
STAT5A
CASP10
FGF7
PEX6
RHO
NME1
CYBRD1
ENPP1
MICALL2
HSPA1A
CD33
NAT2
VPS13A

KIF24
GCG
LEF1
NQO1
HEPH
CCL20
CLCA4
RB1
CNTLN
CCNA2
FBL
MKI67
ABCG2
CROCC
NPPA
SETX
CD69
PNPLA6
NAGLU
TTBK2
USB1
GFI1B
PMS2
TCF7L2
DKK1
IGFBP1
ABCA1
STXBP2
SCN5A
PLXNC1
ADAMTS13
KLF6
SLC51A
SLC4A2
UROS
SLC25A4
UQCRB
AGXT
IRF7
MMP8
TMPRSS6
UCHL1
FABP4
UCP2
SLC25A37
ACSL4
NDUFAF1
CCR2
CDH2
P4HB
ISG15
SYP
CYS1
SERPINA7
WNT1
GSTA1
PEX10
MYH9

CEL
HLA-DRA
PEX3
DVL1
DDB1
MME
CYP27A1
SCD
NUP210
FIP1L1
ERBB3
HPSE
SSB
CDKN2B
AURKA
BCAR1
RO60
ACIN1
IL17F
GPBAR1
TMEM165
TUBB6
UNC13D
TULP3
FBF1
AKR1B10
DNASE1
DPYD
GPI
GPS2
MAGEA3
AVPR2
PHKB
LAMP1
NR0B1
CASP1
ELK1
PTK2B
TGM2
MAGT1
BMP7
GSR
C3
SSTR2
ACACA
GJB1
MT-CO1
ITGA5
GATAD1
HARS1
PEX11B
URGCP
LCAT
FKTN
FOXA2
MGMT
HYLS1
SLC11A1

ADCY6
LDB3
MAGEA1
GFAP
RASGRP1
BAK1
CFH
PRKCB
LRAT
CPQ
STMN1
SCT
HMGCL
BAD
LBP
COX15
PEX16
SLC51B
NKX2-5
SRY
SOS1
VTN
NFKB2
UGT1A7
GH1
WNT3A
U2AF1
CDKN1C
ACTG1
ITGAL
TUBB2B
SFRP1
WT1-AS
F9
CEP295
PRDM16
AURKB
ARID1B
SPART
TYK2
PEX2
THY1
MT-ND5
ARL13A
AXIN2
PEX14
PEX26
PEX12
MT-ND2
GSTM3
CLTRN
E2F2
ITCH
PIK3CB
EPX
FRZB
PLA2G6
FAT4

ANPEP
WNT11
ZEB2
IRF9
INF2
MDK
MYH6
LGALS3BP
KIF12
IL10RA
PODXL
AKT3
PKM
SERPINA6
ARSH
AZIN1
HSD3B1
CPA1
CYP8B1
PHB
ISG20
UBE2O
IGFBP5
HK1
IFNGR1
TUBB1
PUS1
PRODH
ABCC8
ADM
ATAD3A
RUNX3
TRAM2
CCR3
SULT1A3
OSTM1
LRBA
SELL
SNAI2
HOGA1
MAN1B1
ACAD11
KCNAB2
RMND1
FGFR4
TLR6
BHMT
TFAM
KEAP1
FTH1
IFT81
TUBB4A
BIRC3
TUBG1
DHX30
POC1B
PRRT2
GGTLC1

PHOX2A
KIF21A
LIPC
HLF
